# Phylogenetic analysis and development of molecular markers for five medicinal *Alpinia* species based on complete plastome sequences

**DOI:** 10.1186/s12870-021-03204-1

**Published:** 2021-09-22

**Authors:** Heyu Yang, Liqiang Wang, Haimei Chen, Mei Jiang, Wuwei Wu, Shengyu Liu, Jiehua Wang, Chang Liu

**Affiliations:** 1 School of Environmental Science and Engineering, Tianjin University, 300072 Tianjin, China; 2 Institute of Medicinal Plant Development, Chinese Academy of Medical Sciences and Peking Union Medical College, 100193 Beijing, People’s Republic of China; 3 College of Pharmacy, Heze University, Shandong Province 274015 Heze, People’s Republic of China; 4 Guangxi Botanical Garden of Medicinal Plants, 530023 Nanning, Guangxi China; 5 Department of Medical Data Sharing, Institute of Medical Information & Library, Chinese Academy of Medical Sciences & Peking Union Medical College, 100020 Beijing, China

**Keywords:** Plastome, *Alpinia*, Phylogenomic analysis, Species authentication

## Abstract

**Background:**

*Alpinia* species are widely used as medicinal herbs. To understand the taxonomic classification and plastome evolution of the medicinal *Alpinia* species and correctly identify medicinal products derived from *Alpinia* species, we systematically analyzed the plastome sequences from five *Alpinia* species. Four of the *Alpinia* species: *Alpinia galanga* (L.) Willd., *Alpinia hainanensis* K.Schum., *Alpinia officinarum* Hance, and *Alpinia oxyphylla* Miq., are listed in the Chinese pharmacopeia. The other one, *Alpinia nigra* (Gaertn.) Burtt, is well known for its medicinal values.

**Results:**

The four *Alpinia* species: *A. galanga*, *A. nigra*, *A. officinarum*, and *A. oxyphylla*, were sequenced using the Next-generation sequencing technology. The plastomes were assembled using Novoplasty and annotated using CPGAVAS2. The sizes of the four plastomes range from 160,590 bp for *A. galanga* to 164,294 bp for *A. nigra*, and display a conserved quadripartite structure. Each of the plastomes encodes a total of 111 unique genes, including 79 protein-coding, 28 tRNA, and four rRNA genes. In addition, 293–296 SSRs were detected in the four plastomes, of which the majority are mononucleotides Adenine/Thymine and are found in the noncoding regions. The long repeat analysis shows all types of repeats are contained in the plastomes, of which palindromic repeats occur most frequently. The comparative genomic analyses revealed that the pair of the inverted repeats were less divergent than the single-copy region. Analysis of sequence divergence on protein-coding genes showed that two genes (*acc*D and *ycf*1) had undergone positive selection. Phylogenetic analysis based on coding sequence of 77 shared plastome genes resolves the molecular phylogeny of 20 species from Zingiberaceae. In particular, molecular phylogeny of four sequenced *Alpinia* species (*A. galanga*, *A. nigra*, *A. officinarum*, and *A. oxyphylla*) based on the plastome and nuclear sequences showed congruency. Furthermore, a comparison of the four newly sequenced *Alpinia* plastomes and one previously reported *Alpinia* plastomes (accession number: NC_048461) reveals 59 highly divergent intergenic spacer regions. We developed and validated two molecular markers Alpp and Alpr, based on two regions: *pet*N-*psb*M and *psa*J-*rpl*33, respectively. The discrimination success rate was 100 % in validation experiments.

**Conclusions:**

The results from this study will be invaluable for ensuring the effective and safe uses of *Alpinia* medicinal products and for the exploration of novel *Alpinia* species to improve human health.

**Supplementary Information:**

The online version contains supplementary material available at 10.1186/s12870-021-03204-1.

## Background

Zingiberaceae is the largest plant family in the order Zingiberales [1]. It contains about 1,587 species and 52 genera (The Plant List; last accessed: February 2021). The family provides essential natural resources to humans, including many useful products for food, spices, medicines, dyes, perfume, and aesthetics [2, 3]. *Alpinia* Roxb. is the largest, most widely distributed, and most taxonomically complex genus in the Zingiberaceae, including 230 species occurring throughout tropical and subtropical Asia [4]. *Alpinia* comprises approximately 54 species in China. Many of the *Alpinia* species are well-known medicinal herbs. Other *Alpinia* species have been widely used for bioprospection of plant essential oils for medicinal uses [5].

The Chinese pharmacopeia (2020 version) contains 15 Zingiberaceae species belonging to five genera, and four of the 15 species belong to the genus *Alpinia*. These four species are *A. galanga*, *A. officinarum*, *A. oxyphylla*, and *A. hainanensis*. The first species, *A. galanga*, also called “Hong Dou Kou,“ has been used to manage dyspepsia, fever, urinary incontinence, halitosis, and hoarseness of voice in throat infections [6]. The second species, *A. officinarum*, has been used to relieve stomachache, treat colds, invigorate the circulatory system, and reduce swelling. Many chemical constituents have been isolated from this plant, including monoterpenes, diarylheptanoids, flavonoids, phenylpropanoids, and neolignans [7]. The third species, *A. oxyphylla*, also called “Yi Zhi,” is widely used to treat dyspepsia, diarrhea, abdominal pain, spermatorrhea, kidney asthenia, and poor memory [8]. The fourth species, *A. hainanensis* is native to the Hainan Island in Southern China. It has been used for its anti-emetic and stomachic mechanism of action [9]. Another species, *A. nigra* has been used traditionally to treat bronchitis, gastric ulcers, parasitic intestinal infections. However, it is not included in the Chinese pharmacopeia (2020 version) [10]. The morphological identification of these species is problematic. Misidentification will undermine the efficacy and safety of medicinal products developed from them [11, 12]. Additionally, the genetic divergence among these species and the complex evolutionary history of the genus are often poorly understood, making it difficult for the bioprospecting of medicinal *Alpinia* species.

Plastomes provide a robust framework that can be used to examine phylogenetic relationships among plants and provide new probes for species identification [13, 14]. Their comparatively conserved and well-defined genome structures allow the investigation of a wide range of crucial issues. Initially, genetic studies focused on understanding each plastid genome, particularly of the overview features, such as genome size, gene content, and sequence repetition [15]. Lately, the crucial role of the plastomes in the evolution and impact for speciation has become obvious demonstrated by the sequence divergence, large inversion, differences in coding and intergenic regions, and evolutionary analysis [16, 17].

To date, complete plastomes are available from more than 100 Zingiberaceae species, including four *Alpinia* species. Recently, a complete plastome of *A. oxyphylla* (NC_035895) was analyzed, and the plastome shared the highest sequence similarity of > 90 % to that of *A. zerumbet* [18]. Based on the single nucleotide polymorphism (SNP) matrix among 28 whole plastomes, including a plastome (NC_048461) of *A. hainanensis* and two plastomes (NC_035895, MK262729) of *A. oxyphylla*, a phylogenetic analysis showed that *Alpinia* and *Amomum* are closely related in the family Zingiberaceae [19]. Such results provided useful information to understand the *Alpinia* evolution. However, they have not focused on the species that are widely used for their medicinal values and there is no phylogenetic analysis using nuclear markers in *Alpinia* species.

In previous reports, phylogeny, biodiversity assessment within populations, and the authentication of *Alpinia* species have been studied using several molecular markers. Nuclear ribosomal DNA internal transcribed spacers (ITS) sequences have been used as markers to distinguish *A. galanga* from its adulterants (Zhao et al. 2001). Efficacy of DNA barcode internal transcribed spacer 2 (ITS2) was tested on species identification of *Alpinia* species from Peninsular Malaysia [20]. Also, the information of genetic relatedness was developed using seven plastid barcoding loci among wild *Alpinia nigra* (Gaertn.) B.L. Burtt populations [21].

Lately, chloroplast-derived DNA markers were developed to authenticate medicinal plants. One example is SNPs and insertion-deletion mutations (Indels) of the intergenic regions in the plastome of *Panax ginseng* species [22, 23]. However, there are no systematic studies to develop molecular markers for medicinal *Alpinia* species. Our short-term goal is to understand the taxonomic relationship of medicinal *Alpinia* species and develop molecular markers for their discrimination. And our long-term goal is to develop a method for ensuring the efficacy and safety of *Alpinia* medicinal products and identify new *Alpinia* species for medicinal uses. In this study, we reported and compared the four complete plastome sequences of *A. galanga*, *A. nigra*, *A. officinarum*, and *A. oxyphylla* sampled from Guangxi, China. The phylogenetic relationships of medicinal *Alpinia* species were studied based on plastome sequences and single-copy nuclear genes. Molecular markers based on plastomes were furtherly developed for the discrimination of the five *Alpinia* species and were validated successfully.

## Results

### Features of the *Alpinia* species plastomes

**Fig. 1:**
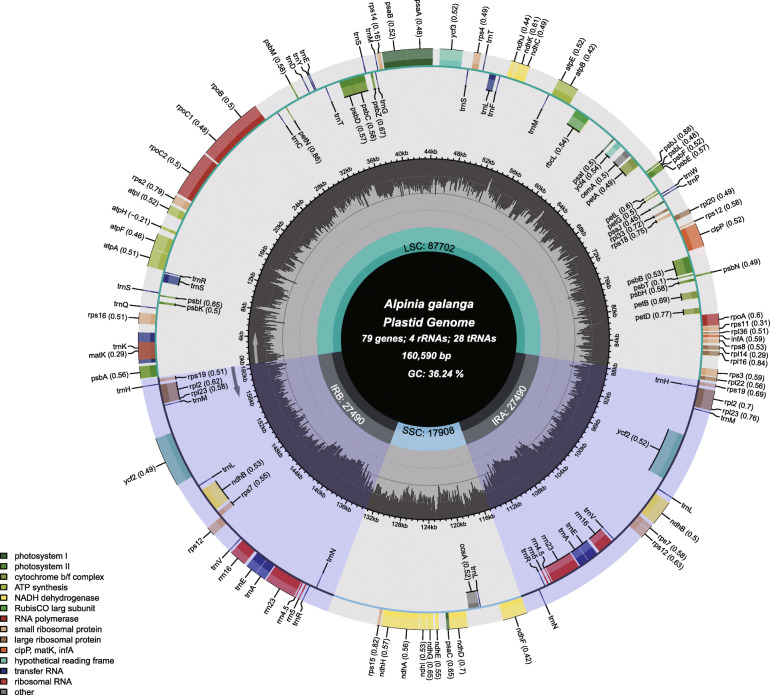
Gene map of the *Alpinia galanga* plastome. The first circle shows the species name and specific information regarding the genome (length, GC content, and the number of genes) from the center going outward. The optional GC content is depicted as the proportion of the shaded parts of each section and the length of the corresponding single short copy (SSC), inverted repeat (IRa and IRb), and large single-copy (LSC) regions are also given in this circle. The outer circle shows the gene names and their optional codon usage bias. The genes are colored based on their functional categories. Genes inside and outside of the circle are transcribed in clockwise and counterclockwise directions, represented with arrows. The optional shaded area stretching from the inner sphere toward the outer circle marks the IR regions

The plastomes are circular structures of 160,590 bp (*A. galanga*), 164,294 bp (*A. nigra*), 162,140 bp (*A. officinarum*), and 161,394 bp (*A. oxyphylla*) long. The schematic representation of the plastomes is shown in Fig. 1 and Figures S1, S2 and S3, respectively. The four plastomes display the typical quadripartite characters and show a high degree of conservation in organization and structure. They consist of a Large Single-Copy (LSC) region (87,267 − 88,970 bp) and a Small Single-Copy (SSC) region (15,349 − 17,908 bp), which were separated by two Inverted Repeat (IR) regions (27,490–29,951 bp) (Table 1). The overall GC contents of *A. galanga*, *A. nigra*, *A. officinarum*, and *A. oxyphylla* plastomes are 36.24 %, 35.98 %, 36.14 %, and 36.16 %, respectively. Whereas the GC contents of their coding sequences (CDS) regions are 37.13 %, 36.91 %, 36.88 %, and 36.95 %, respectively (Table S1), and are somewhat higher than those of the whole plastomes.

**Table 1:** General features of the four *Alpinia* plastomes

	*A. galanga*	*A. nigra*	*A. officinarum*	*A. oxyphylla*
GeneBank Accession number	MK940825	MK940826	MK940823	MK940824
Plastome Length (bp)	160,590	164,294	162,140	161,394
LSC^a^ Length (bp)	87,702	88,970	87,267	87,293
SSC^b^ Length (bp)	17,908	15,422	15,349	16,177
IR^c^ Length (bp)	27,490	29,951	29,762	28,962
Number of Genes	135	135	135	135

^a^*LSC* Large Single-Copy region

^b^*SSC* Small Single-Copy region

^c^*IR* Inverted Repeat region

All of the four *Alpinia* plastomes encode a set of 111 unique genes with identical gene order and gene clusters. Seventy-nine of these are protein-coding genes, 28 are tRNA genes, and four are rRNA genes (Tables S2, S3, S4 and S5). Fourteen genes (*atp*F, *ndh*A, *ndh*B, *pet*B, *pet*D, *rpl*2, *rpo*C1, *rps*16, *trn*A-UGC, *trn*C-ACA, *trn*E-UUC, *trn*K-UUU, *trn*L-UAA, *trn*S-CGA) contain one intron, while three genes, *clp*P, *ycf*3 and *rps*12, possess two introns (Tables S6, S7, S8 and S9, Figures S4, S5, S6 and S7). In particular, the *rps*12 is generated by trans-splicing and has three exons (Figures S4, S5, S6 and S7, lower panels). We also detected 2-262 heteroplasmic sites with minor allele frequency (MAF) of 0.6-1 % in four sequenced *Alpinia* species (Figures S8, S9, S10 and S11).

### Sequence repetition in the *Alpinia* plastomes

Comparative analysis of sequence repetition between all four plastomes found that the overall distribution, types, and numbers of repeats are highly similar among the plastomes. Simple sequence repeats (SSRs) are sequences composed of repeats with motifs from 1 to 6 bp in length. They are widespread in plastomes and widely utilized for species identification, genetic linkage construction, and molecular breeding [24]. A total of 293–296 SSRs were found in the *Alpinia* plastomes (Table 2). The most abundant mononucleotide SSRs are polyadenine or polythymine repeat types. Interestingly, hexanucleotide SSRs were not found in the plastomes of *A. galanga* and *A. oxyphylla* but were detected in the other two *Alpinia* plastomes. Further analysis of the size and location of the different SSR units and comparison revealed that the composite SSR was variable among the four species, while the dinucleotide repeat of AT was conserved (Tables S10, S11, S12, S13 and S14).

**Table 2:** Type and number of Simple-Sequence Repeat (SSRs) found in the four *Alpinia* plastomes

Type	Repeat Unit	Numbers of Repeats			
		*A. galanga*	*A. nigra*	*A. officinarum*	*A. oxyphylla*
Mono-	A/T	172	179	178	177
	C/G	7	6	7	7
Di-	AT/AT	62	65	64	64
	AC/GT	2	1	1	1
	AG/CT	21	21	22	0
Tri-	AAT/ATT	1	1	0	0
	AAG/CTT	3	3	3	3
	AG C/CTG	0	0	0	0
	AG G/CCT	1	0	1	1
	ACT/AGT	1	1	0	0
Tetra-	AAAC/GTTT	1	0	1	1
	AAAG/CTTT	3	4	1	3
	AAAT/ATTT	10	6	9	9
	AACT/AGTT	1	1	1	1
	AATG/ATTC	1	2	2	1
	AATT/AATT	3	1	1	1
	ACAT/ATGT	1	1	1	1
Penta-	AAAAT/ATTTT	0	0	0	0
	AAATC/ATTTG	0	0	0	0
	AAATT/AATTT	0	0	1	1
	AACCC/GGGTT	0	0	0	0
	AATAT/ATATT	3	1	0	0
Hexa-	AATATT/AATATT	0	0	0	0
	AAATAT/ATATTT	0	0	0	0
	AAGAGG/CCTCTT	0	0	0	0
	ACTATC/AGTGAT	0	0	0	0
	AAATTT/AAATTT	0	0	1	0
	AAAATT/AATTTT	0	1	0	0
Total No.	--	293	294	296	293

Long repeat analyses of four sequenced plastomes showed that 45–49 dispersed repeats were detected, which belong to forward, reverse, complementary and palindromic repeats (Table 3). Forward (direct) and palindrome (inverted) repeats were considerably higher in number than reverse and complement repeats. The majority of these repeats with the repeat length range from 30 to 49 bp were located in intergenic spacer (IGS) regions (Tables S15, S16, S17 and S18). We found the dispersed repeats within those genes were mostly located in the exons but not in the introns. They can potentially facilitate structural rearrangements and develop variability among plastomes in a population [25].

**Table 3:** Dispersed repeat sequences identified in the four *Alpinia* plastomes. REPuter was used to recognize repeat sequences with length ≥ 30 bp and identity ≥ 90%. *F* forward, *P* palindromic, *R* reverse, and *C* complement

Type	Size (bp)	*A. galanga*	*A. nigra*	*A. officinarum*	*A. oxyphylla*
F	30–39	2	5	4	5
	40–49	12	11	7	7
	50–59	1	1	2	2
	60–69	0	0	2	2
	≥70	3	5	0	0
P	30–39	8	3	18	14
	40–49	13	14	8	8
	50–59	1	1	3	3
	60–69	0	0	2	2
	≥70	4	3	0	0
R	30–39	1	1	0	4
	40–49	0	1	0	0
	50–59	0	0	0	0
	60–69	0	0	0	0
	≥70	0	0	0	0
C	30–39	0	0	2	2
	40–49	0	0	0	0
	50–59	0	0	0	0
	60–69	0	0	0	0
	≥70	0	0	0	0
Total	--	45	46	49	49

On average, the numbers of detected tandem repeats range from 28 in *A. officinarum* up to 33 in *A. oxyphylla*. The copy numbers of these repeats range from 1.9 to 5.3 copies per tandem repeat, and the repeat sizes range from 30 to 158 bp per copy (Tables S19, S20, S21 and S22). The tandem repeats were found extensively in the IGS regions.

### Expansion of the IR regions in *Alpinia* plastomes

**Fig. 2:**
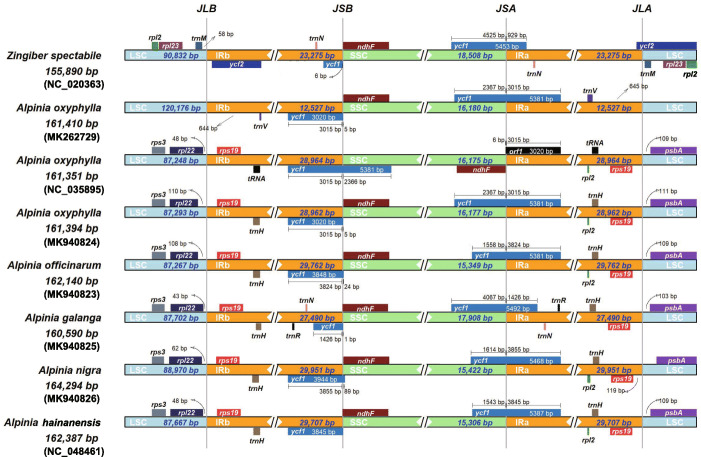
Analysis of the Contraction and Expansion of the IR regions. Schematic representation of the boundary areas of LSC (light blue), IRa (yellow), SSC (green), and IRb (yellow) regions for *Zingiber spectabile*, *A. galanga*, *A. hainanensis*, *A. nigra*, *A. officinarum*, and three *A. oxyphylla* accessions. The species names are shown to the left. The long horizontal blocks represent the plastomes. The name and length of each region are shown. The junction sites between LSC, IRa, SSC, and IRb are marked with the perpendicular dashed line. The genes *rpl*22 (dark blue), *rps*19 (light red), *ycf*1 (blue), *ndh*F (red), and *psb*A (light purple) are shown above the plastomes. The numbers above the gene features denote the distance between the gene borders, either the start or end of genes and the junction sites

The variations in the single-copy and IR regions’ sizes and boundaries commonly cause evolutionary events such as contraction and expansion in the plastome architecture [26]. We compared the IR and single-copy region boundaries among six species, including one *Zingiber* species and the five *Alpinia* plastomes, the four *Alpinia* sequenced in our research, and *A. hainanensis*. Two *A. oxyphylla* genomes sequences previously were included in the analysis. Some divergences were identified among the plastomes of four *Alpinia* species and *Z. spectabile* (Fig. 2). Particularly, IR expansions were found in the LSC/IRa boundary of the four *Alpinia* species, which included the complete *rps*19 gene in the IRs of these species.

In contrast, the *rps*19 gene is located in the LSC region of *Z. spectabile.* The distances between the border of *rps*19 and the IR/LSC junction were 13, 160, 119, 129, and 129 bps in the plastomes of *Z. spectabile*, *A. galanga*, *A. nigra*, *A. officinarum*, and *A. oxyphylla*, respectively. Another interesting observation is that the *ycf*1 gene is localized in the IRb region. The *ycf*1 gene sequence is significantly longer in the *Alpinia* species, 3944 bp for *A. nigra*, 1428 bp for *A. galanga*, and 3944 bp for *A. nigra*, compared with that of *Z. spectabile* (924 bp) (Fig. 2).

### Hypervariable regions

**Fig. 3:**
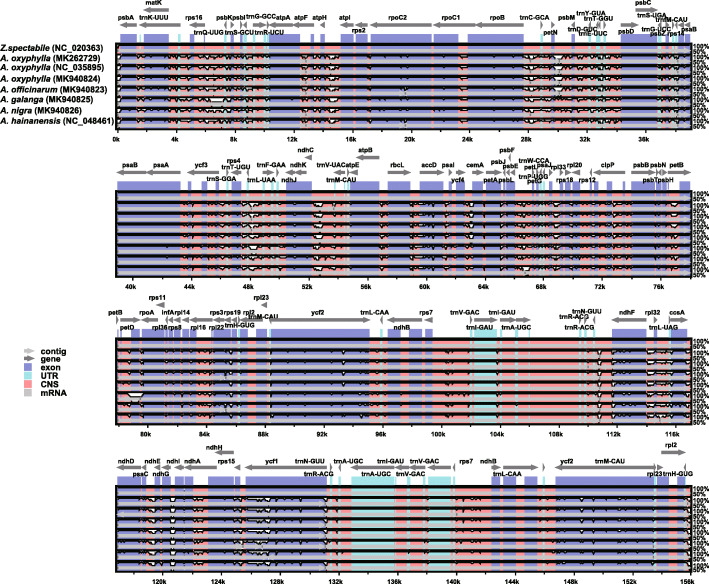
Sequence identity plot of the seven *Alpinia* plastomes with *Zingiber spectabile* as a reference by mVISTA. The species names are shown to the left. The grey arrows above the alignment indicate the transcription direction of genes. In the alignment box, the blue color box indicates protein-coding, the pink color box shows the conserved noncoding sequence, and the light green box indicates tRNAs and rRNAs. The x-axis represents the positions in the cp. genome, and the Y-scale represents the percent identity ranging from 50–100 %

We compared the plastome sequences of five *Alpinia* species, among them, *A. oxyphylla* with three accessions, and *Zingiber* species to determine the overall variations among the *Alpinia* and *Zingiber* species. As shown in Fig. 3, the plastomes are highly conserved among these species. The IR regions were less divergent than the LSC and SSC regions. The coding regions were more conserved than the noncoding regions. However, *ndh*A, *pet*B, *ycf*1, and *ycf*2 genes showed a relatively high degree of sequence divergence. In contrast, the IGS regions were highly diverse, particularly in the following regions: *rps*16-*trn*Q, *pet*N-*psb*M, *psa*C-*ndh*E, *acc*D-*psa*I, *psa*J-*rpl*33, *mat*K-*rps*16, *psb*H-*pet*B (Fig. 3).

**Fig. 4:**
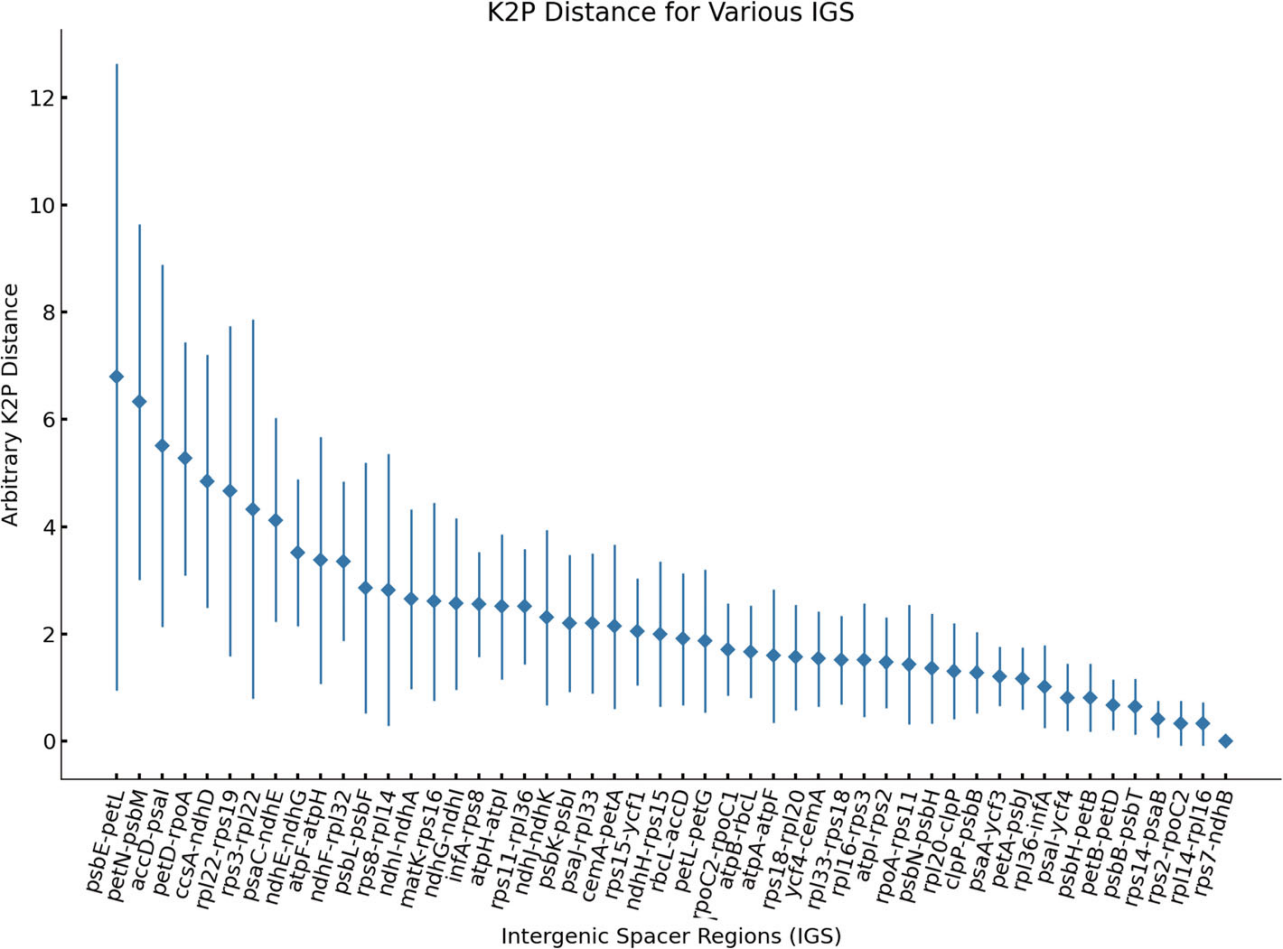
Comparison of the variability of IGS regions among the plastomes of *Alpinia galanga*, *A. hainanensis*, *A. nigra*, *A. officinarum*, and *A. oxyphylla*. The X-axis indicates the IGS regions, and the Y-axis shows the range of K2p distances between different pairs of species. The diamond shows the average K2p distance of the IGS region, respectively

Hypervariable regions can be used to resolve phylogenies and to discriminate closely related plant species [27]. The pairwise comparison of intergenic spacer regions was conducted to identify divergence hotspot regions among the five *Alpinia* species using the Kimura 2-parameter (K2p) model. The average K2p distance ranged from 0.00 to 6.793 among 59 IGSs extracted from these species. Among them, the IGS regions *psb*E-*pet*L, *pet*N-*psb*M, *acc*D-*psa*I, *pet*D-*rpo*A showed the largest distances of 6.79, 6.32, 5.51, and 5.27, respectively (Fig. 4, Table S23).

### Phylogenomic analyses based on plastome data

**Fig. 5:**
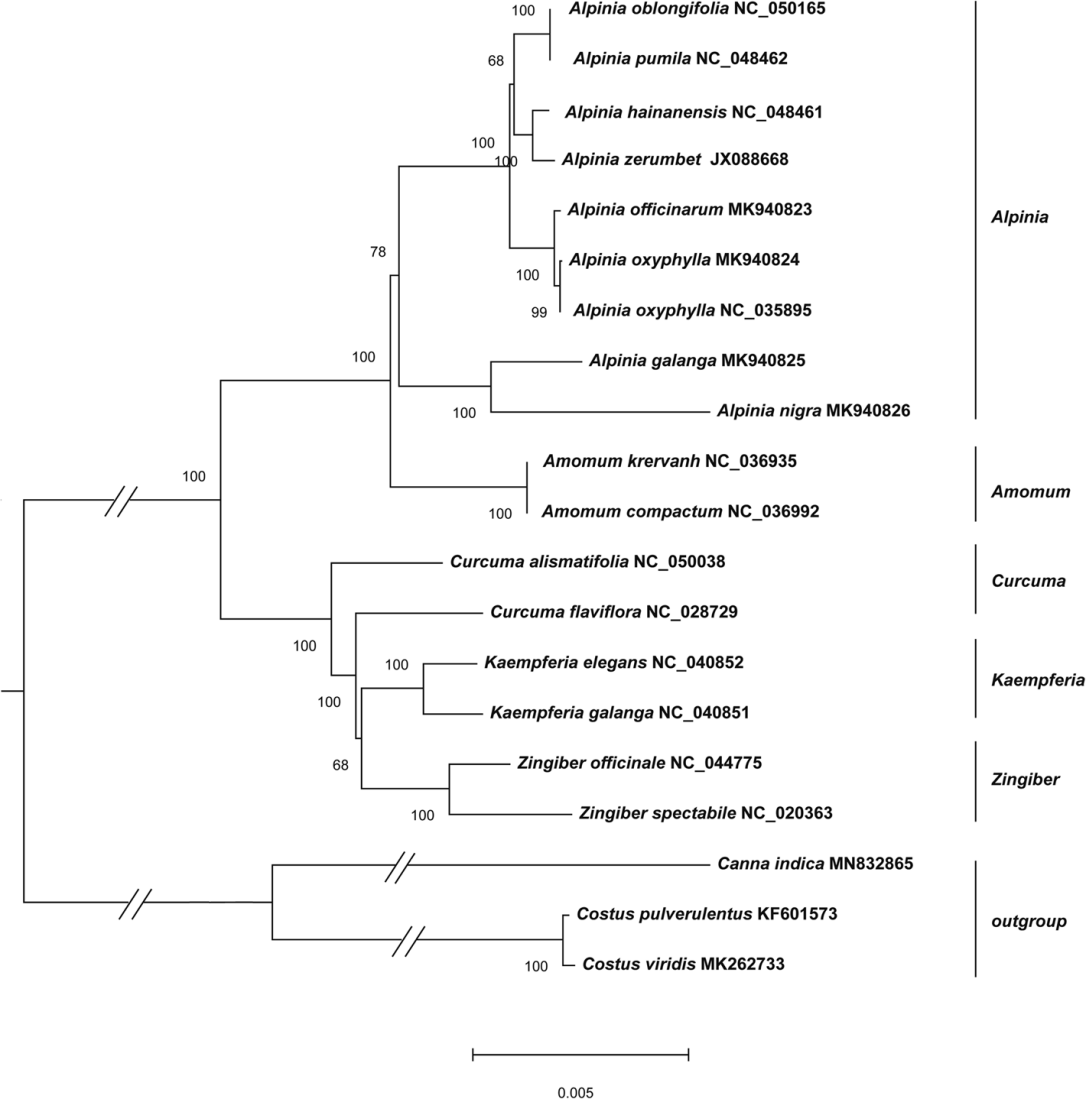
Molecular phylogenetic tree based on common plastid genes. The phylogenetic tree was constructed with 77 shared genes present in 20 species using the maximum likelihood method implemented in Phylosuite. *Costus pulverulentus*, *Costus viridis* and *Canna*, were used as outgroups. Tribes to which each species belongs were shown to the right side of the tree. Bootstrap values were calculated with 1000 replicates

The availability of more complete plastome sequences of *Alpinia* species allows us to conduct phylogenomic analyses with higher resolution in Zingiberaceae (Fig. 5). We performed a phylogenetic analysis using the Maximum likelihood (ML) method based on DNA sequences of 77 genes shared among 20 species from Zingiberaceae, including the four *Alpinia* species sequenced in the study (Table S24). The sister genus of *Alpinia* is *Amomum* with a Bootstrap score (BS) of 100. The species of *Alpinia* are distributed in two main clades. The first clade (BS: 100) is formed by *A. galanga* and *A. nigra*, both medicinal species. The second clade (BS: 100) contains most of the species sampled to date. These species are from the tropical and subtropical geographic regions and many species are of medicinal value. Two accessions of *A. oxyphylla* are clustered together (BS: 99), which are subsequently clustered together with *A. officinarum* (Fig. 5). Also, the phylogenetic positions of *A. galanga* and *A. nigra* were reported for the first time based on the plastomes. The bootstrap scores are high for all branches indicating the high degree of reliability of the phylogenetic tree.

### Phylogenetic analysis based on nuclear markers

**Fig. 6:**
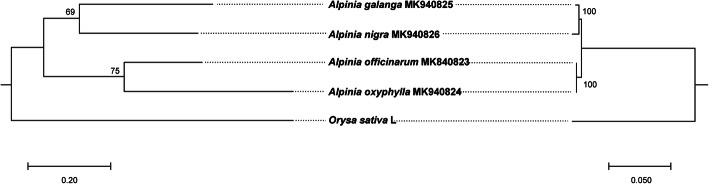
Phylogenetic trees based on common genes identified using HybPiper pipeline and the shared DNA sequences of 77 protein-coding genes in the plastomes for the same five species. The phylogenetic tree on the left panel was constructed with the sequences of 4 shared contigs for nuclear genes present in 4 *Alpinia* species found by the HybPiper pipeline using the maximum likelihood method implemented in Phylosuite. The *Oryza sativa* L. was used as the outgroup. Bootstrap support scores were calculated from 1000 replicates. And the phylogenetic tree on the right panel was constructed with the shared DNA sequences of 77 protein-coding genes in the plastomes of the same five species in the nuclear tree using the same methods in phylogenomic analysis

The low-coverage sequence data generated from this study allowed us to perform phylogenetic analysis using additional nuclear markers. We extracted nuclear genes from sequence data among the Angiosperms-mega 353 gene set [28]. Among these genes, 352, 353, 353, 352 genes had mapped reads, and the reads mapped to 173, 28, 93, 59 genes were assembled into contigs for *A. galanga*, *A. nigra*, *A. officinarum*, and *A*. *oxyphylla*, respectively. Among these assembled contigs, only four genes (AT4G04780, AT3G53760, AT5G53800, AT1G06240) were shared among the four species. These four genes were used to construct a phylogenetic tree using the same method as that for the complete plastome sequences. The reconstructed ML tree with these four genes was well resolved overall. And two of the nodes were supported with bootstrap values of 75 and 69 % (Fig. 6). Among the four *Alpinia* species, *A. galanga* was sister to *A. nigra*, and *A. officinarum* was sister to *A*. *oxyphylla*. To compare if the relationships in both the nuclear and plastome trees are consistent, the phylogenetic analysis of plastomes with the same taxon sampling as the nuclear tree was conducted. The relevant result was consistent with the results of phylogenetic inferences obtained with nuclear markers (Fig. 6). This approach enabled us to define further the phylogenetic relationship between the four *Alpinia* species using nuclear genes.

### Variation and evolutionary selection of protein-coding genes

Purifying/positive selection analyses of 77 protein-coding genes in the *Alpinia* plastomes showed that most genes exhibited ω values less than 0.5. Five genes (*psb*I, *pet*N, *psb*M, *pet*L, and *psb*T) had the lowest ω ratios close to 0. In contrast, the ω values of *ycf*2, *acc*D, *rpl*23, *rps*7, and *ycf*1 were more than 1.00, respectively (Table S25). The results showed that the genes *acc*D and *ycf*1 were under positive selection. The likelihood ratio test identified three and five amino acid sites in *acc*D and *ycf*1 that were positively selected (under posterior probability > 0.95), respectively (Table 4). These sites are also highly polymorphic in the two genes.

**Table 4:** Likelihood ratio tests to identify positively selected sites within the *accD* and *ycf1* genes across 21 *Alpinia* plastomes

Gene	Model compared	Df^a^	-2d*ln*L^b^	*p*-values of LRT^c^	Positively selected sites^d^
*accD*	M1a (neutral) vs. M2a (selection)	2	17.060598	0.000197396	
	M7 (beta) vs. M8 (beta & ω >1)	2	17.143904	0.000189343	1 F 0.995**, 24 I 0.997**, 181 N 0.977*
	M8a (ω=1) vs. M8 (selection)	1	17.051707	0.000036376	
*ycf1*	M1a (neutral) vs. M2a (selection)	2	17.751095	0.000139765	
	M7 (beta) vs. M8 (beta & ω >1)	2	15.933493	0.000346805	3 F 0.964*, 141 F 0.969*, 259 E 0.968*, 303 Y 0.965*, 326 L 0.990**
	M8a (ω=1) vs. M8 (selection)	1	15.839639	0.000068943	

^a^Degree of freedom

^b^Difference between the log likelihood values

^c^*LRT* Likelihood Ratio Test

^d^Sites potentially under positive selection, indicated by the high Empirical Bayes values (‘*’: > 0.95; ‘**’: > 0.99)

### Molecular marker development based on *Alpinia* plastomes

**Fig. 7:**
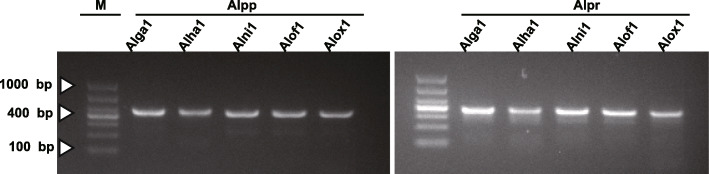
The gel electrophoresis results of the amplification of DNA barcodes using designed primers. Lane M was the marker of DL1000. The lanes from left to right corresponded to products amplificated from the first individual of *A. galanga*, *A. hainanensis, A. nigra*, *A. officinarum*, and *A. oxyphylla* by primer Alpp and Alpr, respectively. The original uncropped image is shown in Figure S12

**Fig. 8:**
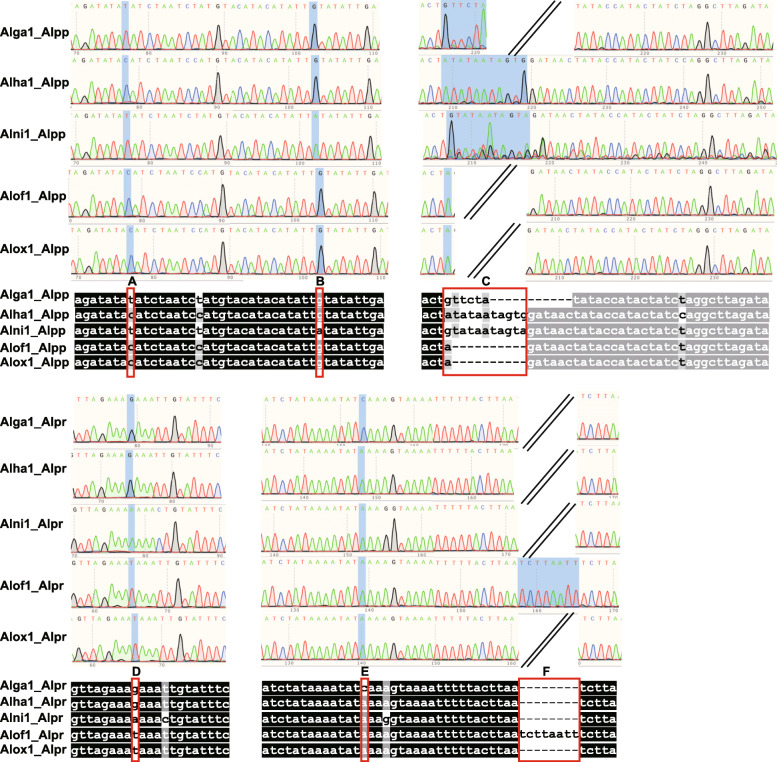
The alignment of the sequencing chromatogram of the PCR products amplified using the primers of Alpp and Alpr. Letters **A-F** represents the SNP and Indel regions, which can distinguish the five *Alpinia* species and are highlighted with red squares. The nucleotides identical across all plastomes are shaded in black, whereas those conserved in 60 % of the sequences are shaded in gray. Alga: *A. galanga*; Alha: *A. hainanensis*; Alni: *A. nigra*; Alof: *Alpinia officinarum*; Alox: *A. oxyphylla*

To discriminate the five medicinal *Alpinia* species, we selected two hypervariable IGS regions, *pet*N-*psb*M, and *psa*J-*rpl*33, to develop two DNA markers named Alpp and Alpr, respectively. The PCR primers used to amplify these two markers are shown in Table S26. PCR amplification of total DNAs from all five medicinal species samples resulted in products having expected size (Fig. 7, Figure S12, Table S27). The DNA fragments were extracted from each band and then subjected to Sanger sequencing. The sequencing results were identical to the expected sequences (Figures S13 and S14). Marker Alpp, derived from the *pet*N-*psb*M IGS region, has two specific SNP loci and one Indel loci. These three variable loci can be used to differentiate three of the five *Alpinia* species, except *A. officinarum* and *A. oxyphylla*. The marker Alpr, derived from the *psa*J-*rpl*33 IGS region. It has two SNP loci and one Indel loci. When using the SNP and Indel loci from both Alpp and Alpr, all the five species can be differentiated successfully (Fig. 8). We also have tested the new primers on all ten available *Alpinia* plastomes obtained from NCBI and this study in silico. These markers can discriminate all eight species based on the SNP and Indel loci from both Alpp and Alpr (Figures S15 and S16).

## Discussion

Here, we studied five medicinal *Alpinia* species, *Alpinia galanga*, *A. hainanensis*, *A. officinarum*, *A. oxyphylla*, and *A. nigra.* We sequenced the four plastomes of these five species. Three of them belonging to *Alpinia galanga*, *A. officinarum*, and *A. nigra* were reported for the first time. Two plastomes of *A. oxyphylla* were released during the study period. We carried out a detailed analysis of the genome features, performed the phylogenetic analysis with plastid proteomes and nuclear makers. Lastly, we developed a set of two primers that can distinguish these five medicinal species.

Compared to the plastomes of previously published *Alpinia* species, all the plastomes presented in this study exhibited consistent genomic structure, gene order, and content. And there are no significant structural rearrangements, such as inversions or gene relocations (Fig. 1, Table S1). The size of the *A*. *oxyphylla* plastomes (MK940824) in this study is almost identical to the other two reported plastomes, which were 161,394 bp (MK940824), 161,410 bp (MK262729) [19], and 161,351 bp (NC_035895) [18]. We found that the most abundant mononucleotide SSRs are of polyadenine or polythymine repeat types in the four *Alpinia* species, consistent with those reported previously [29]. Plastomes are well-arranged, except for the expansion of the IR regions in the *Alpinia* species. Judged from comparative analysis with the plastome of *Z. spectabile* as a reference, the IR lengths of all the four *Alpinia* species plastomes were all increased to ≥ 160 kbp. Also, one evidence supporting this expansion is that the *rps19* gene has moved to the IR regions. In other species of *Alpinia*, plastomes reported so far [18, 19], the entire *rps19* gene is also localized in the IR region, which is consistent with our findings. The analysis revealed that the four *Alpinia* species sequenced in our study have heteroplasmy sites in their plastomes. However, the positions of these detected heteroplasmic sites and two developed molecular markers did not overlap.

Classifications and phylogenetic analysis among Zingiberaceae were previously reported based on morphological features and DNA sequences of the nuclear internal transcribed spacer (ITS) and plastid *mat*K regions [1, 30–32]. We use four new plastome sequences to define the position of four *Alpinia* species in Zingiberaceae. The new accession of *A. oxyphylla* sequenced (MK 940,824) in this study was most closely related to the other one *A. oxyphylla* plastomes reported previously (NC_035895) [19]. To date, the phylogenetic inference of *Alpinia* species has mainly relied on plastid markers [18, 19] and few multi-copy nuclear ribosomal regions such as ITS [33]. Our phylogenetic analysis results create reliable phylogenies of the four *Alpinia* species sequenced by us using the nuclear markers for the first time. In addition, phylogenetic analysis using plastome and nuclear sequences revealed the identical phylogenetic relationships for the four *Alpinia* species.

Because of the lack of mobility, plants must deal with the challenge of abiotic stresses, such as soil salinity, drought, and extreme temperature. Many genes from plastomes, such as *clp*P [34], *rbc*L [35], and *mat*K [36], *ycf*1 and *ycf*2 [37], have been positively selected. The positive selection of the plastome genes may serve as an adaptive evolution for adjusting to environmental changes. In the selective pressure analysis, five genes were positively selected, and their selection might reflect the adaptive evolution of these *Alpinia* species. The results are consistent with the reports that *acc*D and *ycf*1 evolved under positive selection in the *Zingiber* plastomes [37]. Particular amino acids were identified to have been positively selected in two genes, *acc*D, and *ycf*1. For example, the plastid *acc*D is an essential gene required for leaf development [38], and the *ycf*1 is crucial for plant viability [39]. In the current research, all four *Alpinia* species studied distributed in tropical and subtropical areas. Their living environment’s high temperature and humidity may be the reason for the positive selection of the *acc*D and *ycf*1 genes.

One of our goals is to develop markers that can distinguish the five medicinal *Alpinia* species. DNA markers derived from the plastomes have been widely used and are considered highly discriminatory for species identification such as *Panax* and *Cruciata*, including SNPs and InDels [22, 40]. So far, these plastome-derived DNA markers are usually used to analyze intraspecies level diversity and phylogenetic analysis in *Alpinia* [20, 21]. The most variable regions of the complete plastome can be used for DNA barcoding of closely related plant species [27]. Therefore, we developed the specific markers for discriminating *Alpinia* species based on the plastomes’ hypervariable regions. The hypervariable regions identified in our study, such as *pet*N-*psb*M, *psa*C-*ndh*E, *acc*D-*psa*I, were similar to those reported previously [19]. We found two markers derived from the *pet*N-*psb*M and *psa*J-*rpl*33 IGS regions that successfully distinguished the five *Alpinia* species. The marker Alpp1 can’t discriminate *between A. officinarum* and *A. oxyphylla*, because they are more closely related than with the other studied species. It has to be used combined with the marker Alpr1 for successful discrimination of the five *Alpinia* species.

Only a handful of *Alpinia* plastomes are sequenced and available in databases. Because the genus includes more than 200 spp., the information on the phylogeny of the genus is still rather limited. The complete *Alpinia* plastome sequences provided in this study expanded the taxonomic sampling and subsequently formulated new hypotheses about new potential relationships among *Alpinia* taxa [41]. From this point forward, additional plastomes of *Alpinia* species should be sequenced, which allow us to take a broad view of the evolutionary relationship and evolutionary processes of *Alpinia* species, lay the foundation for the further usage of these plants for the benefit of human lives. In this study, we developed molecular markers for the five *Alpinia* species that are of economic importance. With the identification of additional economically important *Alpinia* species, the same methodology can be used to identify their corresponding differentiating markers.

## Conclusions

The complete plastomes of *A. galanga*, *A. nigra*, and *A. officinarum* are reported for the first time in this study. In addition, two molecular markers were developed from the hypervariable regions that can distinguish these five medicinal *Alpinia* species. The results obtained from these studies will contribute to our understanding of *Alpinia* classification, plastome evolution, and the discrimination of medicinal products derived from *Alpinia* species.

## Methods

### Plant materials and total DNA preparation

Fresh leaves were collected from plants grown in the Guangxi Medicinal Plant Garden in Nanning, Guangxi, China (108°19’ E, 22°51’ N, 530,023), for the four species: *A. galanga*, *A. nigra*, *A. officinarum*, and *A. oxyphylla.* We collected these samples from five individual plants with different genotypes for each species for sequencing. The samples were silica-dried and stored at the Herbarium of the Institute of Medicinal Plant Development (voucher numbers: Implad201910413, Implad201910414, Implad20180327, and Implad20180362). To develop molecular markers of *Alpinia* species, we collected fresh leaves of another group from Guangxi Medicinal Plant Garden in Nanning, Guangxi, China, and the ginger garden of South China Botanical Garden, China (113°36’ E, 23°18’ N, 510,650). All samples were collected with permission from the Garden authorities. Detailed information is shown in Table S27. A plant genomic DNA kit (Tiangen Biotech, Beijing, Co., Ltd.) was used to extract total DNAs. The purity of total DNA was evaluated using electrophoresis on 1.0 % agarose gels. And the concentration was measured using a Nanodrop spectrophotometer 2000 (Thermo Fisher Scientific Inc., Waltham, MA, USA). This study complies with relevant institutional, national, and international guidelines and legislation.

### Plastome sequencing, assembly, and annotation

The sequencing libraries of total DNA from each species were prepared using the TruSeq DNA Sample Prep Kit (Illumina, Inc., San Diego, CA, USA) following the manufacturer’s instructions. The total DNA was sheared into fragments at approximately 500 bp long for paired-end library construction. The libraries were sequenced on an Illumina HiSeq 3000 platform (Illumina Inc., San Diego, CA, USA). After obtaining the paired-end reads (2 × 250 bp), we downloaded the plastid genomes from the GenBank database (https://www.ncbi.nlm.nih.gov/genome/organelle/). These plastome sequences were used to search against Illumina paired-end reads using BLASTn with an E-value cutoff of 1e-5. The filtered reads were considered plastome-related and used for the downstream genome assembly. SPAdes (v. 3.10.1) [42] and CLC Genomics Workbench (v. 7, QIAGEN, Aarhus, Denmark) were used for *de novo* assembly. The dot plot of the contigs and reference genome were constructed and visualized for evaluating the assembly quality. The contigs were subjected to reassembly using the Seqman module of Lasergene (v. 11.0, Madison, Wisconsin). Only one contig was obtained for each of the Alpinia species.

We used the CpGAVAS2 web server [43] was used to annotate the four genomes. Cutoffs for the E-values of BLASTn and BLASTx were set to 1e-10. After the pre-filtering step, the number of top hits for annotation included in the reference gene sets was set to 10. Manual corrections were performed to determine the positions of the start and stop codons and the intron/exon boundaries. Codon usage frequency and GC content (i.e., the relative content of guanines and cytosines) were calculated using custom scripts. Their circular gene maps were drawn by the cpgview web server (http://www.herbalgenomics.org/cpgview/). The raw data and the annotated plastomes have been submitted to GenBank. The accession numbers of raw data were SRR9072115 (*A. galanga*), SRR9072120 (*A. nigra*), SRR9080445 (*A. officinarum*), and SRR9080447 (*A. oxyphylla*). The accession numbers of annotated plastomes were MK940825 (*A. galanga*), MK940826 (*A. nigra*), MK940823 (*A. officinarum*), and MK940824 (*A. oxyphylla*). We tested heteroplasmy patterns used NOVOPlasty in the four sequenced *Alpinia* species.

### Repeat sequence analysis

SSRs were detected using the MISA Perl Script (http://pgrc.ipk-gatersleben.de/misa/). The minimal numbers of repeat units are eight for mononucleotide repeats, four for di- and trinucleotide repeats, and three for tetra-, penta-, and hexanucleotide repeats. Long repeat sequences with a minimal length of 30 bp and hamming distance = 3 were predicted using REPuter [44]. Tandem repeat structures were scanned with Tandem Repeats Finder [45]. We set the parameters to 2 for matches and 7 for mismatches and Indels. In contrast, we set the minimum alignment score and maximum period size to 50 and 500, respectively. The minimum repeat size was 30 bp, and the cutoff for similarities among the repeat units was 90 %. All of the identified repeat structures were verified manually. Nested or redundant repeats were removed.

### Phylogenomic analysis

For phylogenetic analyses, the DNA sequences of 77 protein-coding genes from 21 species of the Zingiberaceae family were extracted from the whole plastome sequences and aligned using MAFFT v.7 [46]. The 77 genes included *acc*D, *atp*A, *atp*B, *atp*E, *atp*F, *atp*H, *atp*I, *ccs*A, *cem*A, *clp*P, *inf*A, *mat*K, *ndh*A, *ndh*B, *ndh*C, *ndh*D, *ndh*E, *ndh*F, *ndh*G, *ndh*H, *ndh*I, *ndh*J, *ndh*K, *pet*A, *pet*B, *pet*D, *pet*G, *pet*L, *pet*N, *psa*A, *psa*B, *psa*C, *psa*I, *psa*J, *psb*A, *psb*C, *psb*D, *psb*E, *psb*F, *psb*H, *psb*I, *psb*J, *psb*K, *psb*L, *psb*M, *psb*N, *psb*T, *rbc*L, *rpl*14, *rpl*16, *rpl*2, *rpl*20, *rpl*22, *rpl*23, *rpl*32, *rpl*33, *rpl*36, *rpo*A, *rpo*B, *rpo*C1, *rpo*C2, *rps*11, *rps*12, *rps*14, *rps*15, *rps*16, *rps*18, *rps*19, *rps*2, *rps*3, *rps*4, *rps*7, *rps*8, *ycf*1, *ycf*2, *ycf*3, and *ycf*4. All aligned gene sequences were concatenated, and the best-fit evolutionary model (JTT + F + I + G4) was selected following the Bayesian information criterion (BIC) scores computed by ModelFinder [47]. The maximum likelihood (ML) tree was constructed by IQTREE v1.6.10 [48] with 1000 non-parametric bootstrap replications, and *Costus pulverulentus*, *Costus viridis*, and *Canna indica* as the outgroup taxa. Finally, the consensus tree was visualized using the MEGA X software [49].

### Identification of nuclear markers for phylogenetic analysis

To explore the phylogenetic relationship implied by single-copy nuclear markers, we used the HybPiper v1.2 [50] to identify nuclear markers among the Angiosperms-mega 353 gene set [28] from our sequencing reads for the four *Alpinia* species and then used them for phylogenetic analysis. The command line is “./reads_first.py -b mega353.fasta -r sample_001.fastq sample_002.fastq --prefix sample_result –bwa”. The HybPiper package contains an internal reference set of 353 genes. It can identify genes from high-throughput sequencing results that are homologous to these 353 genes and extract them for phylogenetic analysis. In particular, the expanded Angiosperms353 target file [28], which is a drop-in replacement for the original Angiosperms353 file [51] in the HybPiper analyses, was used to capture loci in our sequence reads. We identified the potential genes for phylogenetic analysis as follows. Firstly, we used the retrieval script in HybPiper to identify contigs matching each probe (https://github.com/mossmatters/HybPiper). This was done using the reads_first.py script. Secondly, the common genes among the four species were selected. Finally, the contigs of these genes were used to create a phylogeny. Briefly, a phylogenetic tree was constructed with the contigs of these nuclear genes as above by IQTREE v1.6.10 [48] with 1000 non-parametric bootstrap replications, except the best-fit model is HKY + F and the outgroup is *Oryza sativa* L. The phylogenetic analyses based on these nuclear sequences were conducted for the sample taxa as those based on the shared coding sequences of 77 protein-coding genes in the plastomes.

### Selective pressure analysis

The levels of selective pressure for a protein-coding gene are measured by the ratio of nonsynonymous to synonymous substitutions (ω) [15]. To detect the *Alpinia* plastid genes that were under positive selection, we extracted 77 protein-coding genes common to the 21 Zingiberaceae plastomes, performed multiple sequence alignment using MAFFT, and constructed a maximum likelihood (ML) tree using IQTREE v1.6.10 [48]. Then we calculated the ratio of nonsynonymous (dN), synonymous (dS) and ω (dN/dS) values were using CodeML in PAML Version 4.9 [52] with a One-ratio model (model = 0, seqtype = 1, NSsites = 0). If the ω value is > 1, the Bayes empirical Bayes (BEB) method implemented in the program EasyCodeML which is called site models (seqtype = 1, model = 0, NSsites = 0, 1, 2, 3, 7, 8) [53] were used to identify positively selected sites.

### Identification of the hypervariable regions

We conducted a comparative genome analysis for the complete *Alpinia* plastomes using the software mVISTA (http://genome.lbl.gov/vista/mvista/submit.shtml) in the Shuffle-LAGAN mode. The annotated *Z. spectabile* plastome (NC_020363) was used as the reference in the analysis. To identify the most divergent regions, we wrote a custom script to extract the start and end of the IGS regions from the GenBank files for the five plastomes, together with the plastome of *A. hainanensis*. A total of 59 IGSs shared by the five *Alpinia* plastomes were identified. The sequences were extracted and aligned using the ClustalW2 (v. 2.0.12) program with options “-type = DNA -gapopen = 10 -gapext = 2” [54]. Pairwise distances were calculated using the K2p evolution model implemented in the distmat program from the EMBOSS package (v. 6.3.1) [55].

### Identification and validation of molecular markers for species discrimination

We used variable intergenic regions to discriminate the five medicinal *Alpinia* species as a template to develop molecular markers. Primers were designed using the Primer3 program (http://bioinfo.ut.ee/primer3-0.4.0/). PCR amplifications were performed in a final volume of 25 µL with 12.5 µL 2×Taq PCR Master Mix, 0.4 µM of each primer, 2 µL template DNA, and 10.1 µL ddH2O. All amplifications were carried out in a Pro-Flex PCR system (Applied Biosystems, Waltham, MA, USA) under the following conditions: denaturation at 94 ^o^C for 2 min, followed by 35 cycles of 94 ^o^C for 30 s, at specific annealing temperature (Tm) for 30 s, 72 ^o^C for 60 s and 72 ^o^C for 2 min as the final extension. PCR amplicons were visualized on 1.5 % agarose gels and then subjected to Sanger sequencing on an ABI 3730 x l instrument (Applied Biosystems, USA) using the same set of primers used for PCR amplification.

## Supplementary Information

**Additional file 1: Figure S1.** Schematic representation of the *A. nigra *plastome features. **Figure S2.** Schematic representation of the *A. officinarum *plastome features. **Figure S3. **Schematic representation of the *A. oxyphylla *plastome features. **Figure S4.** The schematic diagram of position and length of introns and exons for the splitting genes in the plastome of *A. galanga*. The gene *rps*12 was a trans-splicing gene. **Figure S5.** The schematic diagram of position and length of introns and exons for the splitting genes in the plastome of *A. nigra*. The gene *rps*12 was a trans-splicing gene. **Figure S6. **The schematic diagram of position and length of introns and exons for the splitting genes in the plastome of *A. officinarum*. The gene *rps*12 was a trans-splicing gene. **Figure S7.** The schematic diagram of position and length of introns and exons for the splitting genes in the plastome of *A. oxyphylla*. The gene *rps*12 was a trans-splicing gene. **Figure S8. **The VCF output for the *A. officinarum*. **Figure S9.** The VCF output for the *A. oxyphylla*. **Figure S10.** The VCF output for the *A. oxyphylla*. **Figure S11.** The VCF output for the *A. nigra*. **Figure S12.** The original and full-length gel electrophoresis results of the amplification of DNA barcodes using designed primers. **Figure S13.** The alignment of amplicons produced by designed Alpp primers. **Figure S14. **The alignment of amplicons produced by designed Alpr primers. **Figure S15. **The alignment of amplicons in 10 *Alpinia *plastomes produced by designed Alpp primers in silico. **Figure S16.** The alignment of amplicons in 10 *Alpini*a plastomes produced by designed Alpp primers in silico.

**Additional file 2: Table S1.** Base composition in the plastomes of four Alpinia species. **Table S2.** List of genes annotated in the plastome of A. galanga. Numbers in parentheses represented the repetition of genes. Superscript T: trans-splicing gene. **Table S3.** List of genes annotated in the plastome of A. nigra. Numbers in parentheses represented the repetition of genes. Superscript T: trans-splicing gene. **Table S4.** List of genes annotated in the plastome of A. officinarum. Numbers in parentheses represented the repetition of genes. Superscript T: trans-splicing gene. **Table S5.** List of genes annotated in the plastome of A. oxyphylla. Numbers in parentheses represented the repetition of genes. Superscript T: trans-splicing gene. **Table S6.** The length of introns and exons for the splitting genes in the plastome of A. galanga. The gene rps12 was a trans-splicing gene. **Table S7. **The length of introns and exons for the splitting genes in the plastome of A. nigra. The gene rps12 was a trans-splicing gene. **Table S8. **The length of introns and exons for the splitting genes in the plastome of A. officinarum. The gene rps12 was a trans-splicing gene. **Table S9.** The length of introns and exons for the splitting genes in the plastome of A. oxyphylla. The gene rps12 was a trans-splicing gene. **Table S10.** SSR identified in the plastome of A.galanga. P1 = Mononucleotide; P2 = Di nucleotide; P3 = Tri nucleotide; P4 = Tetra nucleotide; P5 = Penta nucleotide; 6 = Hexa nucleotide repeats and c = Compound repeat microsatellites. **Table S11.** SSR identified in the plastome of A.nigra. P1 = Mononucleotide; P2 = Di nucleotide; P3 = Tri nucleotide; P4 = Tetra nucleotide; P5 = Penta nucleotide; 6 = Hexa nucleotide repeats and c = Compound repeat microsatellites. **Table S12.** SSR identified in the plastome of A. officinarum. P1 = Mononucleotide; P2 = Di nucleotide; P3 = Tri nucleotide; P4 = Tetra nucleotide; P5 = Penta nucleotide; 6 = Hexa nucleotide repeats and c = Compound repeat microsatellites. **Table S13. **SSR identified in the plastome of A. oxyphylla. P1 = Mononucleotide; P2 = Di nucleotide; P3 = Tri nucleotide; P4 = Tetra nucleotide; P5 = Penta nucleotide; 6 = Hexa nucleotide repeats and c = Compound repeat microsatellites. **Table S14. **Comparison of SSR markers found among four Alpinia species and one outgroup species of Zingiber spectabile. Zisp: Zingiber spectabile; Alga: Alpinia galanga; Alni: Alpinia nigra; Alof: Alpinia officinarum; Alox: Alpinia oxyphylla. **Table S15.** Dispersed repeat sequences in the plastome of A. galanga. **Table S16. ** Dispersed repeat sequences in the plastome of A.nigra. **Table S17.** Dispersed repeat sequences in the plastome of A. officinarum. **Table S18.** Dispersed repeat sequences in the plastome of A. oxyphylla. **Table S19.** Tandem repeat sequences identified in the plastome of A. galanga. **Table S20.** Tandem repeat sequences identified in the plastome of A. nigra. **Table S21.** Tandem repeat sequences identified in the plastome of A.officinarum. **Table S22.** Tandem repeat sequences identified in the plastome of A.oxyphylla. a: coding sequences; b: intergenic spacers. **Table S23.** The distances among the shared intergenic spacer (IGS) regions from the five Alpinia plastomes. Alga: Alpinia galanga; Alha: Alpinia hainanensis; Alni: Alpinia nigra; Alof: Alpinia officinarum; Alox: Alpinia oxyphylla. **Table S24.** The list of accession numbers of the plastome sequences used in the phylogenetic analyses of the Zingiberaceae. **Table S25.** The dN, dS and dN/dS (ω) value of 77 commom protein-coding genes from plastomes of 21 Alpinia species. **Table S26.** The two pairs of primers for the ampilification of DNA barcodes. **Table S27.** The list of sample numbers of the samples used in the species discrimination analyses of the Alpinia.

## Data Availability

The datasets generated during the current study are available in the GenBank: MK940823 and https://www.ncbi.nlm.nih.gov/nuccore/MK940823.1 for *Alpinia officinarum*, MK940824 and https://www.ncbi.nlm.nih.gov/nuccore/MK940824.1 for *A. oxyphylla*, MK940825 and https://www.ncbi.nlm.nih.gov/nuccore/MK940825.1 for *A. galanga*, MK940826 and https://www.ncbi.nlm.nih.gov/nuccore/MK940826.1 for *A. nigra*, respectively. Raw sequence data for this study also can be found in GenBank. The associated BioProject, SRA, and Bio-Sample numbers and the associated links are PRJNA543348, SRS4779543, SAMN11664063, and https://www.ncbi.nlm.nih.gov/bioproject/543348 for *Alpinia officinarum*; PRJNA543352, SRS4779545, SAMN11664207, and https://www.ncbi.nlm.nih.gov/bioproject/543352 for *A. oxyphylla*; PRJNA543223, SRS4772960, SAMN11658378, and https://www.ncbi.nlm.nih.gov/bioproject/543223 for *A. galanga*; PRJNA543223, SRS4772960, SAMN11658378, and https://www.ncbi.nlm.nih.gov/bioproject/543239 for *A. nigra*, respectively.

## Abbreviations

SSRs: Simple sequence repeats
IR: Inverted repeat
LSC: Long Single Copy
SSC: Short Single Copy
IGS: Intergenic spacer
dN: The ratio of nonsynonymous
dS: The ratio of synonymous
ω: dN/dS
SNP: Single nucleotide polymorphism
Indel: Insertion-deletion mutations
ITS: Internal transcribed spacer
tRNA: Transfer RNA
rRNA: Ribosomal RNA
