# Supplementary material for: Phylogenetic analysis and development of molecular markers for five medicinal Alpinia species based on complete plastome sequences
Source: BMC Plant Biol. 2021 Sep 22;21:431. doi: 10.1186/s12870-021-03204-1 (PMC8456601; doi:10.1186/s12870-021-03204-1)
Supplement: Supplementary file 1 — Additional file 1: Figure S1. Schematic representation of the A. nigra plastome features. Figure S2. Schematic representation of the A. officinarum plastome features. Figure S3. Schematic representation of the A. oxyphylla plastome features. Figure S4. The schematic diagram of position and length of introns and exons for the splitting genes in the plastome of A. galanga. The gene rps12 was a trans-splicing gene. Figure S5. The schematic diagram of position and length of introns and exons for the splitting genes in the plastome of A. nigra. The gene rps12 was a trans-splicing gene. Figure S6. The schematic diagram of position and length of introns and exons for the splitting genes in the plastome of A. officinarum. The gene rps12 was a trans-splicing gene. Figure S7. The schematic diagram of position and length of introns and exons for the splitting genes in the plastome of A. oxyphylla. The gene rps12 was a trans-splicing gene. Figure S8. The VCF output for the A. officinarum. Figure S9. The VCF output for the A. oxyphylla. Figure S10. The VCF output for the A. oxyphylla. Figure S11. The VCF output for the A. nigra. Figure S12. The original and full-length gel electrophoresis results of the amplification of DNA barcodes using designed primers. Figure S13. The alignment of amplicons produced by designed Alpp primers. Figure S14. The alignment of amplicons produced by designed Alpr primers. Figure S15. The alignment of amplicons in 10 Alpinia plastomes produced by designed Alpp primers in silico. Figure S16. The alignment of amplicons in 10 Alpinia plastomes produced by designed Alpp primers in silico. [file 12870_2021_3204_MOESM1_ESM.pdf]

TITLE

Phylogenetic analysis and development of molecular markers for five medicinal *Alpinia* species based on complete plastome sequences

AUTHOR LIST

Heyu Yang[1,3#], Liqiang Wang[2,3#], Haimei Chen[3], Mei Jiang[3], Wuwei Wu[4], Shengyu Liu[5], Jiehua Wang[1]* and Chang Liu[3]*

[1]School of Environmental Science and Engineering, Tianjin University, Tianjin 300072, China.

[2]College of Pharmacy, Heze University, Heze 274015, Shandong Province, P.R. China.

[3]Institute of Medicinal Plant Development, Chinese Academy of Medical Sciences and Peking Union Medical College, Beijing 100193, PR China.

[4]Guangxi Botanical Garden of Medicinal Plants, Nanning, Guangxi 530023, China.

[5]Institute of Medical Information & Library, Chinese Academy of Medical Sciences & Peking Union Medical College, Beijing 100193, china.

*Correspondence: Chang Liu: cliu6688@yahoo.com ; Tel: +86-10-57833111; Fax: +86-10-62899715；Jiehua Wang: jiehuawang@tju.edu.cn

#Heyu Yang and Liqiang Wang contributed equally to this work.

E-mails:

HYY: 18700944808@163.com
LW: lys832000@163.com
HC: hmchen@implad.ac.cn
MJ: mjiang0502@163.com
WW: wuweiwu2013@163.com
SYL: shengyuliu558@163.com
JHW: jiehuawang@tju.edu.cn
CL: cliu6688@yahoo.com

Supplementary tables

Table S1. Base composition in the plastomes of the four *Alpinia* species.

Table S2. List of genes annotated in the plastome of *A. galanga*. Numbers in parentheses represented the repetition of genes. Superscript T: trans-splicing gene.

Table S3. List of genes annotated in the plastome of *A. nigra*. Numbers in parentheses represented the copy number of genes. Superscript T: trans-splicing gene.

Table S4. List of genes annotated in the plastome of *A. officinarum*. Numbers in parentheses represented the repetition of genes. Superscript T: trans-splicing gene.

Table S5. List of genes annotated in the plastome of *A. oxyphylla*. Numbers in parentheses represented the repetition of genes. Superscript T: trans-splicing gene.

Table S6. The length of introns and exons for the splitting genes in the plastome of *A. galanga*. The gene *rps*12 was a trans-splicing gene.

Table S7. The length of introns and exons for the splitting genes in the plastome of *A. nigra*. The gene *rps*12 was a trans-splicing gene.

Table S8. The length of introns and exons for the splitting genes in the plastome of *A. officinarum*. The gene *rps*12 was a trans-splicing gene.

Table S9. The length of introns and exons for the splitting genes in the plastome of *A. oxyphylla*. The gene *rps*12 was a trans-splicing gene.

Table S10. SSR identified in the plastome of *A. galanga*. P1 = Mononucleotide; P2 = Di nucleotide; P3 = Tri nucleotide; P4 = Tetra nucleotide; P5 = Penta nucleotide; 6 = Hexa nucleotide repeats and c = Compound repeat microsatellites.

Table S11. SSR identified in the plastome of *A. nigra*. P1 = Mononucleotide; P2 = Di nucleotide; P3 = Tri nucleotide; P4 = Tetra nucleotide; P5 = Penta nucleotide; 6 = Hexa nucleotide repeats and c = Compound repeat microsatellites.

Table S12. SSR identified in the plastome of *A. officinarum*. P1 = Mononucleotide; P2 = Di nucleotide; P3 = Tri nucleotide; P4 = Tetra nucleotide; P5 = Penta nucleotide; 6 = Hexa nucleotide repeats and c = Compound repeat microsatellites.

Table S13. SSR identified in the plastome of *A. oxyphylla*. P1 = Mononucleotide; P2 = Di nucleotide; P3 = Tri nucleotide; P4 = Tetra nucleotide; P5 = Penta nucleotide; 6 = Hexa nucleotide repeats and c = Compound repeat microsatellites.

Table S14. Comparison of SSR markers found among four *Alpinia* species and one outgroup species of *Zingiber spectabile*. Zisp: *Zingiber spectabile*; Alga: *Alpinia galanga*; Alni: *Alpinia nigra*; Alof: *Alpinia officinarum*; Alox: *Alpinia oxyphylla*.

Table S15. Dispersed repeat sequences in the plastome of *A. galanga*.

Table S16. Dispersed repeat sequences in the plastome of *A. nigra*.

Table S17. Dispersed repeat sequences in the plastome of *A. officinarum*.

Table S18. Dispersed repeat sequences in the plastome of *A. oxyphylla*.

Table S19. Tandem repeat sequences identified in the plastome of *A. galanga*. a: coding sequences; b: intergenic spacers.

Table S20. Tandem repeat sequences identified in the plastome of *A. nigra*. a: coding sequences; b: intergenic spacers.

Table S21. Tandem repeat sequences identified in the plastome of *A. officinarum*. a: coding sequences; b: intergenic spacers.

Table S22. Tandem repeat sequences identified in the plastome of *A. oxyphylla*. a: coding sequences; b: intergenic spacers.

Table S23. The distances among the shared intergenic spacer (IGS) regions from the five Alpinia plastomes. Alga: *Alpinia galanga*; Alha: *Alpinia hainanensis*; Alni: *Alpinia nigra*; Alof: *Alpinia officinarum*; Alox: *Alpinia oxyphylla*.

Table S24. The list of accession numbers of the plastome sequences used in the phylogenetic analyses of the Zingiberaceae.

Table S25. The dN, dS, and dN/dS (ω) value of 77 common protein-coding genes from plastomes of 21 *Alpinia* species.

Table S26. The two pairs of primers for the amplification of DNA barcodes.

Table S27. The list of sample numbers of the samples used in the species discrimination analyses of the *Alpinia*.

Figure S1. Schematic representation of the *A. nigra* plastome features.

Figure S2. Schematic representation of the *A. officinarum* plastome features.

Figure S3. Schematic representation of the *A. oxyphylla* plastome features.

Figure S4. The schematic diagram of position and length of introns and exons for the splitting genes in the plastome of *A. galanga*. The gene *rps*12 was a trans-splicing gene.

Figure S5. The schematic diagram of position and length of introns and exons for the splitting genes in the plastome of *A. nigra*. The gene *rps*12 was a trans-splicing gene.

Figure S6. The schematic diagram of position and length of introns and exons for the splitting genes in the plastome of *A. officinarum*. The gene *rps*12 was a trans-splicing gene.

Figure S7. The schematic diagram of position and length of introns and exons for the splitting genes in the plastome of *A. oxyphylla*. The gene *rps*12 was a trans-splicing gene.

Figure S8. The VCF output for the *A. officinarum*.

Figure S9. The VCF output for the *A. oxyphylla*.

Figure S10. The VCF output for the *A. oxyphylla*.

Figure S11. The VCF output for the *A. nigra*.

Figure S12. The original and full-length gel electrophoresis results of the amplification of DNA barcodes using designed primers.

Figure S13. The alignment of amplicons produced by designed Alpp primers.

Figure S14. The alignment of amplicons produced by designed Alpr primers.

Figure S15. The alignment of amplicons in 10 *Alpinia* plastomes produced by designed Alpp primers in silico.

Figure S16. The alignment of amplicons in 10 *Alpini*a plastomes produced by designed Alpp primers in silico.

Figure S1. Schematic representation of the *A. nigra plas*tome features.
From the center going outward, the first circle shows the species name and specific information regarding the genome (length, GC content, and the number of genes). The optional GC content is depicted as the proportion of the shaded parts of each section and the length of the corresponding single short copy (SSC), inverted repeat (IRa and IRb), and large single-copy (LSC) regions are also given in this circle. The outer circle shows the gene names and their optional codon usage bias. The genes are colored based on their functional categories. Genes inside and outside of the circle are transcribed in clockwise and counterclockwise directions, represented with arrows. The optional shaded area stretching from the inner sphere toward the outer circle marks the IR regions.

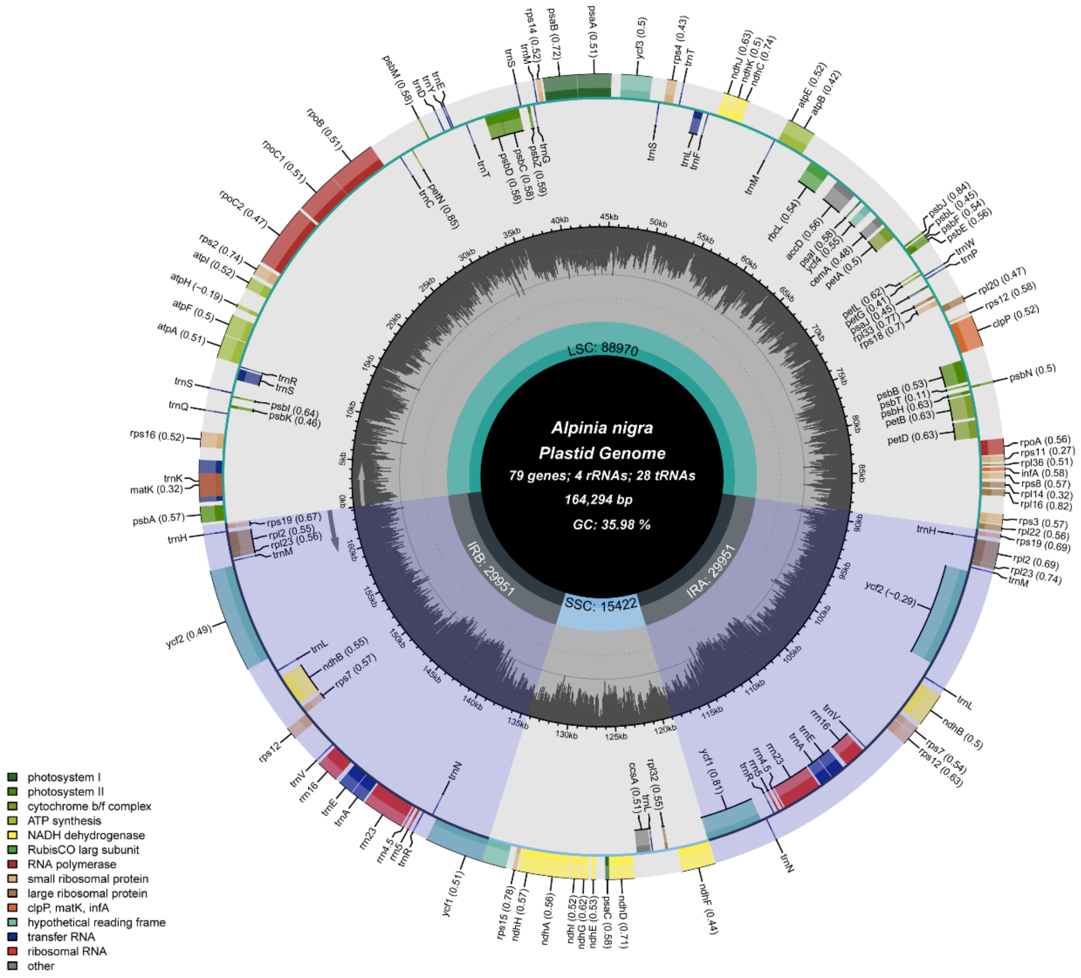

Figure S2. Schematic representation of the *A. officinarum plas*tome features. From the center going outward, the first circle shows the species name and specific information regarding the genome (length, GC content, and the number of genes). The optional GC content is depicted as the proportion of the shaded parts of each section and the length of the corresponding single short copy (SSC), inverted repeat (IRa and IRb), and large single-copy (LSC) regions are also given in this circle. The outer circle shows the gene names and their optional codon usage bias. The genes are colored based on their functional categories. Genes inside and outside of the circle are transcribed in clockwise and counterclockwise directions, represented with arrows. The optional shaded area stretching from the inner sphere toward the outer circle marks the IR regions.

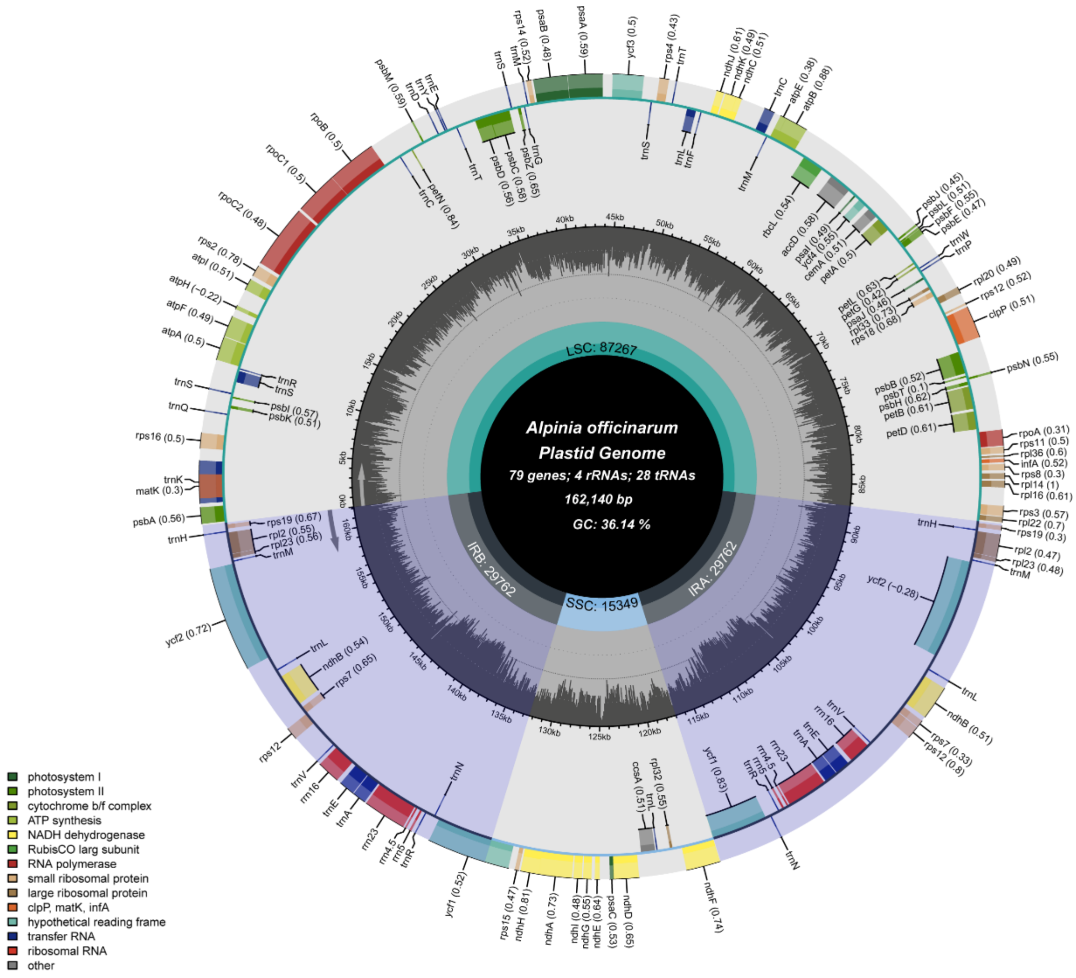

Figure S3. Schematic representation of the *A. oxyphylla* plastome features. From the center going outward, the first circle shows the species name and specific information regarding the genome (length, GC content, and the number of genes). The optional GC content is depicted as the proportion of the shaded parts of each section and the length of the corresponding single short copy (SSC), inverted repeat (IRa and IRb), and large single-copy (LSC) regions are also given in this circle. The outer circle shows the gene names and their optional codon usage bias. The genes are colored based on their functional categories. Genes inside and outside of the circle are transcribed in clockwise and counterclockwise directions, represented with arrows. The optional shaded area stretching from the inner sphere toward the outer circle marks the IR regions.

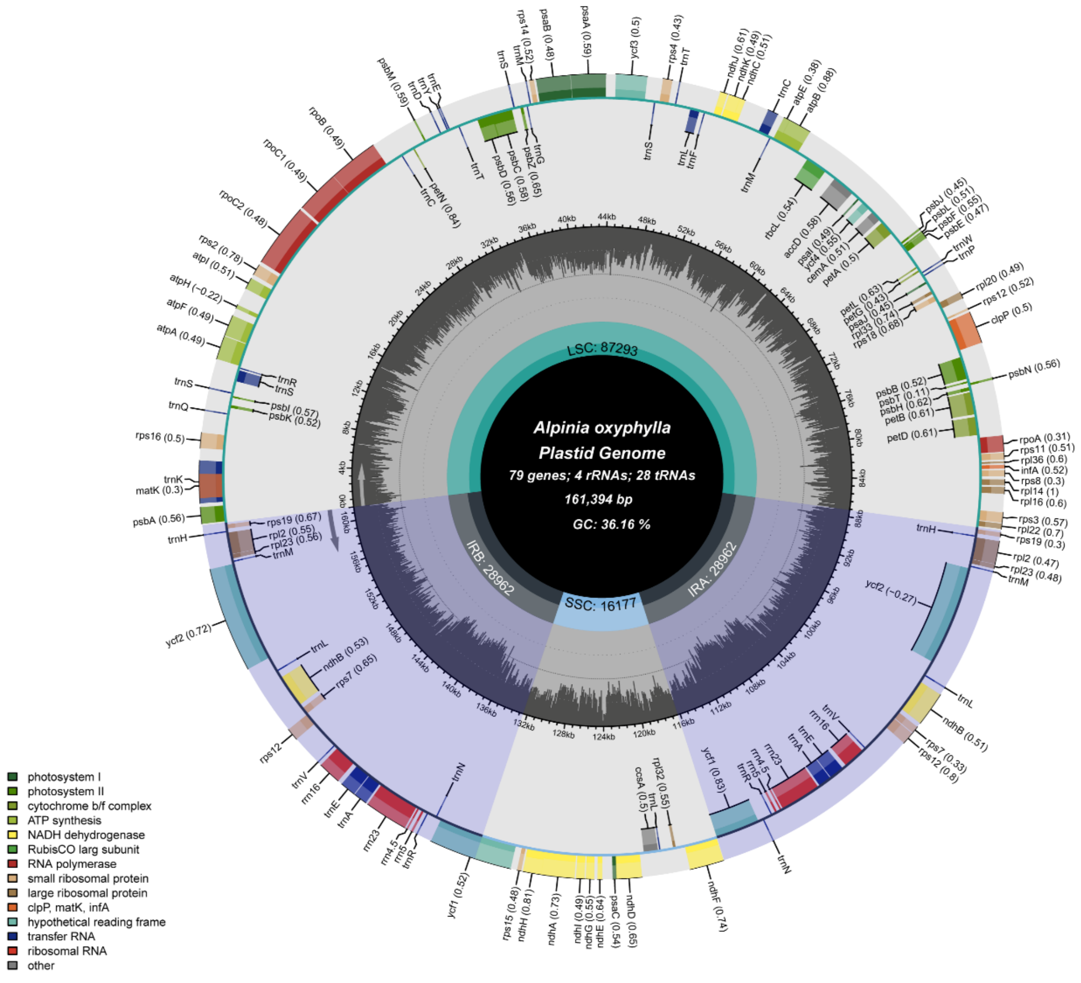

Figure S4. The schematic diagram of position and length of introns and exons for the splitting genes in the plastome of *A. galanga*. The gene *rps*12 was a trans-splicing gene.

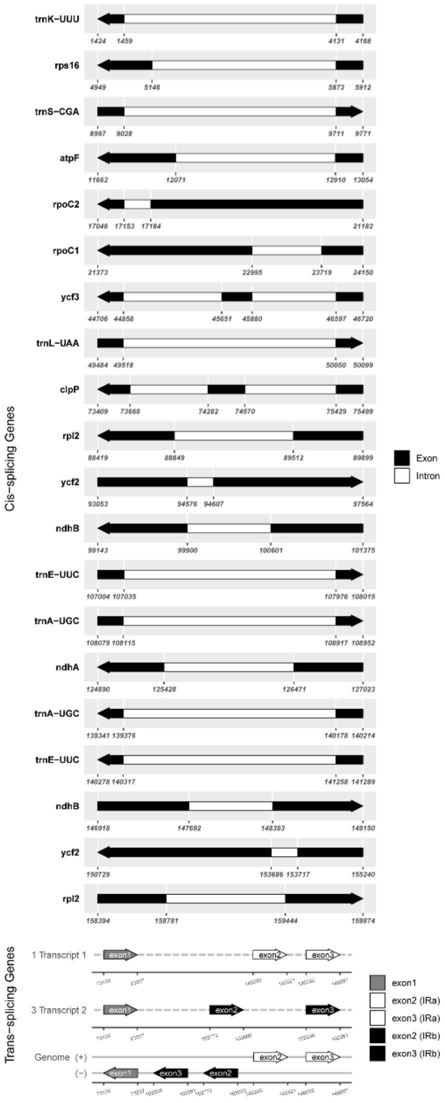

FigureS5. The schematic diagram of position and length of introns and exons for the splitting genes in the plastome of *A. nigra*. The gene *rps*12 was a trans-splicing gene.

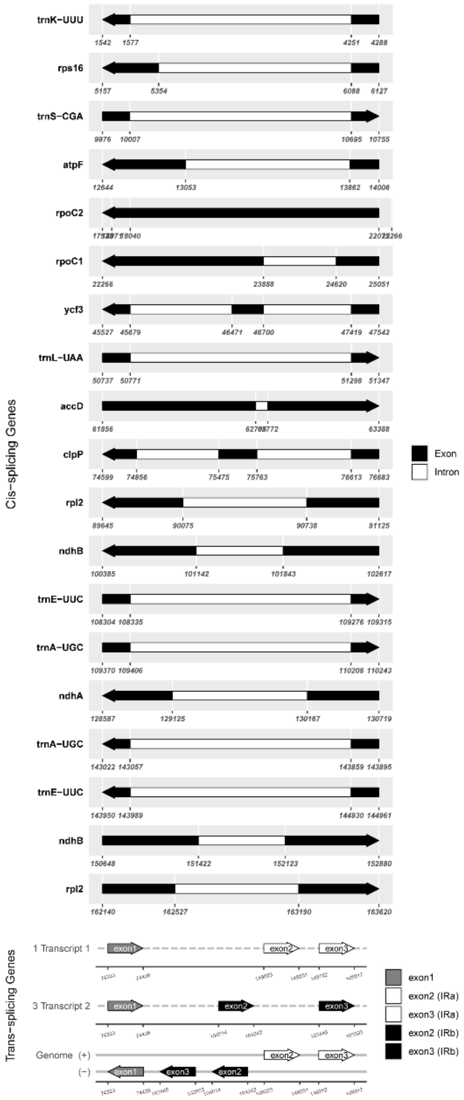

Figure S6. The schematic diagram of position and length of introns and exons for the splitting genes in the plastome of *A. officinarum*. The gene *rps*12 was a trans-splicing gene.

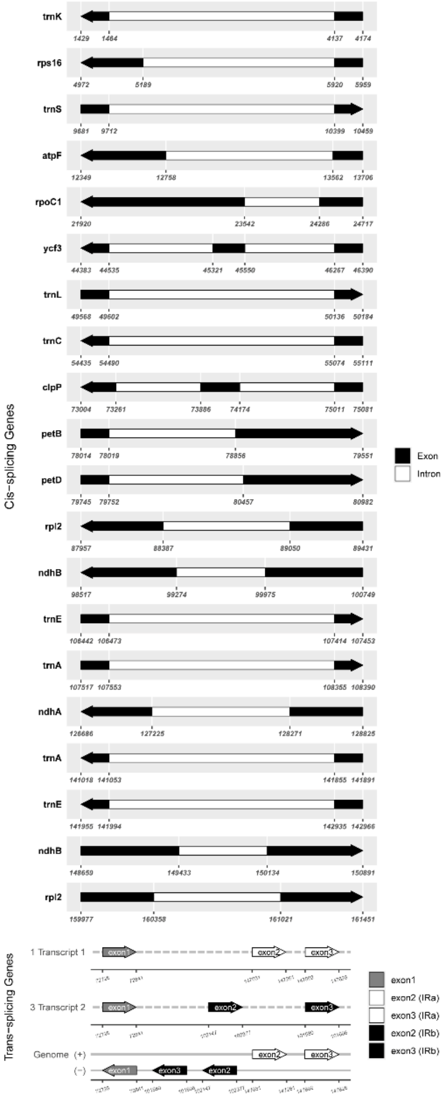

FigureS7. The schematic diagram of position and length of introns and exons for the splitting genes in the plastome of *A. oxyphylla*. The gene *rps*12 was a trans-splicing gene.

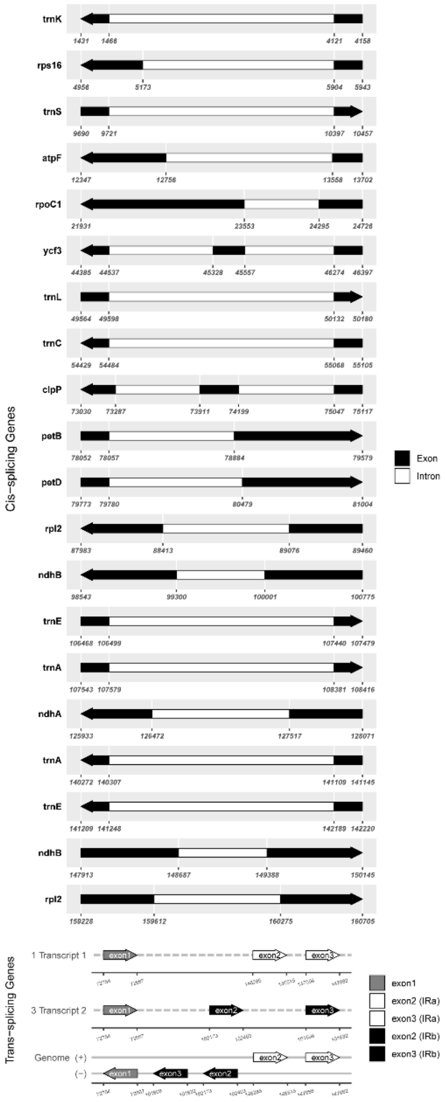

Figure S8. The VCF output for the *A. officinarum*.

The VCF file with all the detected intra-individual polymorphism outputed by the heteroplasmy mode of NOVOPLasty. The allele frequency (AF), depth of coverage (DP) are given as additional info. FR indicates if the polymorphism was detected on the forward and/or reverse strand. LCR will indicate if the polymorphism is situated near a low-complexity region; SNR stands for single nucleotide repeat.

```
##fileformat=VCFv4.0
##fileDate=20210531
##reference=/home/zhangh/my_apps/HybPiper-master/MK940823.fasta
##INFO=<ID=AF,Number=1,Type=Float,Description="Allele Frequency">
##INFO=<ID=DP,Number=1,Type=Integer,Description="Raw Depth">
##INFO=<ID=FR,Number=1,Type=Flag,Description="Detected on the forward(F) and/or reverse(R) strand">
##INFO=<ID=LCR,Number=1,Type=String,Description="Low Complexity Region">
#CHROM  POS  ID  REF  ALT  QUAL FILTER  INFO
  cp    87674    .    A    .    .    AF=;DP=129;FR=R;LCR=SNR
  cp    87680    .    A    .    .    AF=;DP=125;FR=R;LCR=SNR
```

Figure S9. The VCF output for the *A. oxyphylla*.

The VCF file with all the detected intra-individual polymorphism outputed by the heteroplasmy mode of NOVOPLasty. The allele frequency (AF), depth of coverage (DP) are given as additional info. FR indicates if the polymorphism was detected on the forward and/or reverse strand. LCR will indicate if the polymorphism is situated near a low-complexity region; SNR stands for single nucleotide repeat.

```
##fileformat=VCFv4.0
##fileDate=14:13:3606
##reference=/home/zhangh/my_apps/HybPiper-master/MK940824.fasta
##INFO=<ID=AF,Number=1,Type=Float,Description="Allele Frequency">
##INFO=<ID=DP,Number=1,Type=Integer,Description="Raw Depth">
##INFO=<ID=FR,Number=1,Type=Flag,Description="Detected on the forward(F) and/or reverse(R) strand">
##INFO=<ID=LCR,Number=1,Type=String,Description="Low Complexity Region">
#CHROM  POS  ID  REF  ALT  QUAL FILTER  INFO
cp  89024  .  T  G  .  .  AF=0.949;DP=39;FR=R;LCR=SNR
cp  89039  .  T  A,C  .  .  AF=0.976,;DP=41;FR=R;LCR=
cp  89040  .  A  G,T  .  .  AF=0.976,;DP=41;FR=R;LCR=
cp  89195  .  T  A  .  .  AF=0.0511;DP=39;FR=R;LCR=
cp  89197  .  C  A  .  .  AF=0.0511;DP=39;FR=R;LCR=
cp  89327  .  T  G  .  .  AF=0.0447;DP=44;FR=R;LCR=
cp  89457  .  A  C  .  .  AF=0.0761;DP=39;FR=R;LCR=
cp  89630  .  A  C  .  .  AF=0.0403;DP=49;FR=R;LCR=
cp  90064  .  A  T  .  .  AF=0.0447;DP=44;FR=R;LCR=
cp  90409  .  CAATTATA  C  .  .  AF=0.0364;DP=55;FR=R;LCR=
cp  90494  .  G  T  .  .  AF=0.048;DP=41;FR=R;LCR=
cp  90510  .  T  A  .  .  AF=0.0505;DP=39;FR=R;LCR=
cp  90524  .  A  G,T  .  .  AF=0.952,;DP=41;FR=R;LCR=
cp  90525  .
    A,C  .  .  AF=0.977,;DP=43;FR=R;LCR=
cp  91306  .
    C,A  .  .  AF=0.965,;DP=56;FR=R;LCR=
cp  92629  .  G  T  .  .  AF=0.0322;DP=62;FR=R;LCR=
cp  93100  .  T  A  .  .  AF=0.0214;DP=46;FR=R;LCR=
cp  93468  .  T  A  .  .  AF=0.0286;DP=35;FR=R;LCR=
cp  93769  .  C  A  .  .  AF=0.0176;DP=56;FR=R;LCR=
cp  93821  .  G  T  .  .  AF=0.0169;DP=59;FR=R;LCR=
cp  93848  .  A  C  .  .  AF=0.0327;DP=61;FR=R;LCR=
cp  93894  .  A  T  .  .  AF=0.0605;DP=49;FR=R;LCR=
cp  94060  .  C  T  .  .  AF=0.0214;DP=46;FR=R;LCR=
cp  94062  .  C  T  .  .  AF=0.0429;DP=46;FR=R;LCR=
cp  94884  .  G  T  .  .  AF=0.0196;DP=51;FR=F;LCR=
cp  96369  .  T  G  .  .  AF=0.0435;DP=46;FR=F;LCR=
cp  96467  .  A  T  .  .  AF=0.0351;DP=57;FR=F;LCR=
```

```
cp  96534   .  T  A  .  .  AF=0.0358;DP=55;FR=F;LCR=
cp  97616   .  G  A  .  .  AF=0.0402;DP=49;FR=R;LCR=
cp  97916   .  C  A  .  .  AF=0.0428;DP=92;FR=FR;LCR=
cp  98809   .  A  C  .  .  AF=0.0455;DP=44;FR=R;LCR=
cp  98951   .  A  C  .  .  AF=0.0521;DP=38;FR=R;LCR=
cp  100787  .  C  A  .  .  AF=0.0306;DP=65;FR=F;LCR=
cp  100914  .  A  C  .  .  AF=0.0278;DP=36;FR=F;LCR=
cp  101043  .  G  T  .  .  AF=0.0182;DP=54;FR=F;LCR=
cp  101153  .  G  T  .  .  AF=0.0217;DP=46;FR=R;LCR=
cp  101570  .  C  A  .  .  AF=0.0698;DP=43;FR=F;LCR=
cp  101632  .  G  T  .  .  AF=0.0836;DP=47;FR=F;LCR=
cp  101633  .  A  T  .  .  AF=0.0627;DP=47;FR=F;LCR=
cp  101634  .  A  T  .  .  AF=0.0627;DP=47;FR=F;LCR=
cp  101657  .  A  C  .  .  AF=0.0393;DP=50;FR=F;LCR=
cp  101775  .  C  A  .  .  AF=0.0359;DP=55;FR=F;LCR=
cp  102204  .  T  G  .  .  AF=0.0378;DP=52;FR=F;LCR=
cp  102454  .
    C,T  .  .  AF=0.898,;DP=58;FR=F;LCR=
cp  102794  .  T  C  .  .  AF=0.0408;DP=49;FR=F;LCR=
cp  102797  .  A  G  .  .  AF=0.0408;DP=49;FR=F;LCR=
cp  102849  .  C  T  .  .  AF=0.042;DP=47;FR=F;LCR=
cp  102870  .  A  T  .  .  AF=0.044;DP=45;FR=F;LCR=
cp  103038  .  A  G  .  .  AF=0.054;DP=37;FR=F;LCR=
cp  103221  .  G  T  .  .  AF=0.0465;DP=43;FR=F;LCR=
cp  103385  .  G  A  .  .  AF=0.0371;DP=53;FR=F;LCR=
cp  103401  .  C  T  .  .  AF=0.0364;DP=54;FR=F;LCR=
cp  103533  .  C  A  .  .  AF=0.0457;DP=43;FR=F;LCR=
cp  103963  .  C  T  .  .  AF=0.0435;DP=46;FR=F;LCR=
cp  104003  .  C  A  .  .  AF=0.0427;DP=46;FR=F;LCR=
cp  104432  .  A  C  .  .  AF=0.0702;DP=28;FR=F;LCR=
cp  104713  .  T  G  .  .  AF=0.0488;DP=41;FR=F;LCR=
cp  104762  .  G  T  .  .  AF=0.0393;DP=50;FR=F;LCR=
cp  105233  .  A  T  .  .  AF=0.127;DP=55;FR=F;LCR=
```

Figure S10. The VCF output for the *A. galanga*.

The VCF file with all the detected intra-individual polymorphism outputed by the heteroplasmy mode of NOVOPLasty. The allele frequency (AF), depth of coverage (DP) are given as additional info. FR indicates if the polymorphism was detected on the forward and/or reverse strand. LCR will indicate if the polymorphism is situated near a low-complexity region; SNR stands for single nucleotide repeat.

```
##fileformat=VCFv4.0
##fileDate=11:36:2106
##reference=/home/zhangh/my_apps/HybPiper-master/NC_035895.fasta
##INFO=<ID=AF,Number=1,Type=Float,Description="Allele Frequency">
##INFO=<ID=DP,Number=1,Type=Integer,Description="Raw Depth">
##INFO=<ID=FR,Number=1,Type=Flag,Description="Detected on the forward(F) and/or reverse(R) strand">
##INFO=<ID=LCR,Number=1,Type=String,Description="Low Complexity Region">
#CHROM  POS  ID  REF  ALT  QUAL FILTER  INFO
cp  229  .  T  A  .  .  AF=0.0111;DP=90;FR=R;LCR=
cp  234  .  A  C  .  .  AF=0.0404;DP=74;FR=R;LCR=
```

Figure S11. The VCF output for the *A.nigra*.

The VCF file with all the detected intra-individual polymorphism outputed by the heteroplasmy mode of NOVOPLasty. The allele frequency (AF), depth of coverage (DP) are given as additional info. FR indicates if the polymorphism was detected on the forward and/or reverse strand. LCR will indicate if the polymorphism is situated near a low-complexity region; SNR stands for single nucleotide repeat.

```
##fileformat=VCFv4.0
##fileDate=19:46:5906
##reference=/home/zhangh/my_apps/HybPiper-master/MK940825.fasta
```

```
##INFO=<ID=AF,Number=1,Type=Float,Description="Allele Frequency">
##INFO=<ID=DP,Number=1,Type=Integer,Description="Raw Depth">
##INFO=<ID=FR,Number=1,Type=Flag,Description="Detected on the forward(F) and/or reverse(R) strand">
##INFO=<ID=LCR,Number=1,Type=String,Description="Low Complexity Region">
#CHROM	POS	ID	REF	ALT	QUAL	FILTER	INFO
cp	89413	.	A	T	.	.	AF=0.0184;DP=162;FR=R;LCR=SNR
cp	89452	.
	C,A	.	.	AF=0.988,;DP=162;FR=R;LCR=SNR
cp	89783	.	C	A	.	.	AF=0.0311;DP=224;FR=R;LCR=SNR(T)
cp	89832	.	G	T	.	.	AF=0.0135;DP=148;FR=R;LCR=
cp	89833	.	G	T	.	.	AF=0.0128;DP=156;FR=R;LCR=
cp	89900	.	A	C	.	.	AF=0.0185;DP=108;FR=R;LCR=
cp	89990	.	C	A	.	.	AF=0.0167;DP=119;FR=R;LCR=
cp	90217	.	C	A	.	.	AF=0.0129;DP=232;FR=R;LCR=
cp	90316	.	T	A	.	.	AF=0.0133;DP=224;FR=R;LCR=
cp	90780	.	C	A	.	.	AF=0.0173;DP=115;FR=R;LCR=
cp	90833	.	C	A	.	.	AF=0.0185;DP=108;FR=R;LCR=
cp	91245	.	C	T	.	.	AF=0.0217;DP=92;FR=R;LCR=
cp	91535	.	C	A	.	.	AF=0.0156;DP=192;FR=R;LCR=
cp	92306	.	C	A	.	.	AF=0.016;DP=124;FR=R;LCR=
cp	92564	.	G	T	.	.	AF=0.0153;DP=130;FR=R;LCR=
cp	92743	.	C	A	.	.	AF=0.0159;DP=126;FR=R;LCR=
cp	92819	.	C	A	.	.	AF=0.0181;DP=110;FR=R;LCR=
cp	92859	.	A	G	.	.	AF=0.0165;DP=120;FR=R;LCR=
cp	93059	.	A	G	.	.	AF=0.0133;DP=150;FR=R;LCR=
cp	93379	.	C	A	.	.	AF=0.0176;DP=113;FR=R;LCR=
cp	93769	.	G	T	.	.	AF=0.0181;DP=110;FR=R;LCR=
cp	94088	.	G	T	.	.	AF=0.0174;DP=114;FR=R;LCR=SNR
cp	94187	.	G	A	.	.	AF=0.0159;DP=126;FR=R;LCR=
cp	94368	.	G	T	.	.	AF=0.0115;DP=260;FR=R;LCR=
cp	94439	.	C	A	.	.	AF=0.0146;DP=273;FR=R;LCR=
cp	94608	.	C	A	.	.	AF=0.0167;DP=119;FR=R;LCR=SNR
cp	95000	.	C	A	.	.	AF=0.0129;DP=155;FR=R;LCR=
cp	95112	.
		.	.	AF=;DP=126;FR=R;LCR=
cp	96101	.	C	A	.	.	AF=0.016;DP=124;FR=R;LCR=
cp	96495	.	G	C,T	.	.	AF=0.0177,;DP=112;FR=R;LCR=
cp	96569	.	C	A	.	.	AF=0.0157;DP=127;FR=R;LCR=
cp	96609	.	G	T	.	.	AF=0.0156;DP=128;FR=R;LCR=
cp	96675	.	G	T	.	.	AF=0.0164;DP=122;FR=R;LCR=
cp	96876	.	G	A	.	.	AF=0.0155;DP=257;FR=R;LCR=
cp	96943	.	C	A	.	.	AF=0.016;DP=125;FR=R;LCR=
cp	97234	.	G	T	.	.	AF=0.0179;DP=111;FR=R;LCR=
cp	97385	.	G	T	.	.	AF=0.0138;DP=289;FR=R;LCR=
cp	97880	.	G	T	.	.	AF=0.0182;DP=110;FR=R;LCR=
cp	98283	.	G	T	.	.	AF=0.0141;DP=142;FR=R;LCR=
cp	98365	.	G	T	.	.	AF=0.0158;DP=126;FR=R;LCR=
cp	98384	.	G	T,C	.	.	AF=0.0165,;DP=121;FR=R;LCR=
cp	98881	.	G	T	.	.	AF=0.0186;DP=107;FR=R;LCR=
cp	98912	.	G	T	.	.	AF=0.0192;DP=104;FR=R;LCR=
cp	99236	.	G	T	.	.	AF=0.0178;DP=112;FR=R;LCR=
cp	99380	.	C	A	.	.	AF=0.015;DP=133;FR=R;LCR=
cp	99693	.	G	T	.	.	AF=0.017;DP=117;FR=R;LCR=
cp	99698	.	C	A	.	.	AF=0.0183;DP=109;FR=R;LCR=
cp	99927	.	G	T	.	.	AF=0.0152;DP=131;FR=R;LCR=
cp	100269	.	A	G	.	.	AF=0.0113;DP=266;FR=R;LCR=
cp	100705	.	G	T	.	.	AF=0.017;DP=117;FR=R;LCR=
cp	100788	.	C	A	.	.	AF=0.01;DP=298;FR=R;LCR=
cp	100859	.	C	A	.	.	AF=0.0158;DP=126;FR=R;LCR=
cp	101082	.	T	A,C	.	.	AF=0.987,;DP=152;FR=R;LCR=
```

cp	101367	.	C	A	.	.	AF=0.0234;DP=128;FR=R;LCR=
cp	101491	.	C	A	.	.	AF=0.0185;DP=108;FR=R;LCR=
cp	101718	.	G	T	.	.	AF=0.0155;DP=128;FR=R;LCR=
cp	101778	.	C	A	.	.	AF=0.0152;DP=131;FR=R;LCR=
cp	102454	.	G	A	.	.	AF=0.0125;DP=240;FR=R;LCR=
cp	102710	.	G	T	.	.	AF=0.0129;DP=155;FR=R;LCR=
cp	103144	.	T	C	.	.	AF=0.0175;DP=114;FR=R;LCR=
cp	103270	.	C	A	.	.	AF=0.0156;DP=128;FR=R;LCR=
cp	103433	.	A	C	.	.	AF=0.0155;DP=128;FR=R;LCR=
cp	103505	.	C	A	.	.	AF=0.0308;DP=97;FR=R;LCR=SNR(T)
cp	103686	.	CATATTGTAGGGTC	.	.	AF=0.0487;DP=308;FR=R;LCR=	
cp	103993	.	G	T	.	.	AF=0.0113;DP=176;FR=R;LCR=
cp	104347	.	T	G	.	.	AF=0.0465;DP=129;FR=R;LCR=
cp	104484	.	G	T	.	.	AF=0.0177;DP=112;FR=R;LCR=
cp	105072	.					
	G,T	.	.	AF=0.984,;DP=124;FR=R;LCR=			
cp	105391	.	A	G,C	.	.	AF=0.908,;DP=141;FR=R;LCR=
cp	105437	.					
		.	.	AF=;DP=133;FR=R;LCR=			
cp	105853	.					
	G,T	.	.	AF=0.982,;DP=112;FR=R;LCR=			
cp	105854	.	A	C,G	.	.	AF=0.0177,;DP=112;FR=R;LCR=
cp	105889	.	C	A	.	.	AF=0.0326;DP=92;FR=R;LCR=
cp	105912	.	G	C	.	.	AF=0.0326;DP=313;FR=R;LCR=
cp	105928	.	A	C,G	.	.	AF=0.966,;DP=303;FR=R;LCR=
cp	106064	.	G	T	.	.	AF=0.0207;DP=145;FR=R;LCR=
cp	106226	.	C	A	.	.	AF=0.0129;DP=154;FR=R;LCR=
cp	106253	.	G	A	.	.	AF=0.941;DP=136;FR=R;LCR=
cp	106619	.	G	T	.	.	AF=0.0135;DP=147;FR=R;LCR=
cp	106757	.	A	G	.	.	AF=0.0179;DP=111;FR=R;LCR=
cp	107497	.	C	A	.	.	AF=0.016;DP=125;FR=R;LCR=
cp	107550	.	G	C	.	.	AF=0.0166;DP=120;FR=R;LCR=
cp	107819	.	G	T	.	.	AF=0.0177;DP=112;FR=R;LCR=
cp	108374	.	C	A	.	.	AF=0.0144;DP=138;FR=R;LCR=
cp	108429	.	C	A	.	.	AF=0.0171;DP=116;FR=R;LCR=
cp	108809	.	G	T	.	.	AF=0.0129;DP=154;FR=R;LCR=
cp	109167	.	C	A	.	.	AF=0.0147;DP=136;FR=R;LCR=
cp	109188	.	C	A	.	.	AF=0.0173;DP=115;FR=R;LCR=
cp	109459	.	C	A	.	.	AF=0.017;DP=117;FR=R;LCR=
cp	109852	.	C	A	.	.	AF=0.0191;DP=104;FR=R;LCR=
cp	110067	.	C	A	.	.	AF=0.0158;DP=126;FR=R;LCR=
cp	110534	.	A	G,T	.	.	AF=0.985,;DP=132;FR=R;LCR=
cp	111499	.	C	A	.	.	AF=0.0155;DP=128;FR=R;LCR=
cp	111733	.	G	T	.	.	AF=0.0165;DP=120;FR=R;LCR=
cp	111765	.	G	T	.	.	AF=0.018;DP=111;FR=R;LCR=
cp	112099	.	A	G,T	.	.	AF=0.983,;DP=116;FR=R;LCR=
cp	112260	.	G	C,T	.	.	AF=0.984,;DP=126;FR=R;LCR=
cp	112403	.	C	A	.	.	AF=0.016;DP=124;FR=R;LCR=
cp	112442	.	C	A	.	.	AF=0.0176;DP=113;FR=R;LCR=
cp	113139	.	C	A	.	.	AF=0.0157;DP=127;FR=R;LCR=
cp	113146	.	C	A	.	.	AF=0.0174;DP=114;FR=R;LCR=
cp	113157	.	T	G	.	.	AF=0.0257;DP=116;FR=R;LCR=
cp	113194	.	T	G	.	.	AF=0.0264;DP=113;FR=R;LCR=
cp	113195	.	C	A	.	.	AF=0.0264;DP=113;FR=R;LCR=
cp	113274	.	G	C,T	.	.	AF=0.0272,;DP=110;FR=R;LCR=
cp	113281	.	CATTAAAG C	.	.	AF=0.0174;DP=230;FR=R;LCR=	
cp	113323	.	C	G	.	.	AF=0.0296;DP=135;FR=R;LCR=
cp	113330	.	G	T	.	.	AF=0.0142;DP=140;FR=R;LCR=
cp	113780	.	C	A	.	.	AF=0.0151;DP=132;FR=R;LCR=
cp	114159	.	G	T	.	.	AF=0.0156;DP=128;FR=R;LCR=

391	cp	114431	.	G	C	.	.	AF=0.964;DP=277;FR=R;LCR=
392	cp	114548	.	A	G	.	.	AF=0.97;DP=266;FR=R;LCR=SNR(T)
393	cp	114609	.	T	A	.	.	AF=0.978;DP=268;FR=R;LCR=
394	cp	114743	.	G	T	.	.	AF=0.0104;DP=288;FR=R;LCR=
395	cp	115064	.	T	C	.	.	AF=0.0561;DP=160;FR=R;LCR=
396	cp	115071	.	A	C	.	.	AF=0.0518;DP=154;FR=R;LCR=
397	cp	115341	.	T	C,A	.	.	AF=0.99,;DP=193;FR=R;LCR=
398	cp	115358	.	A		.	.	AF=;DP=204;FR=R;LCR=
399	cp	115361	.	T		.	.	AF=;DP=204;FR=R;LCR=
400	cp	115379	.	A	G,T	.	.	AF=0.987,;DP=157;FR=R;LCR=SNR(A)
401	cp	115814	.	C	A	.	.	AF=0.0267;DP=112;FR=R;LCR=
402	cp	115919	.	G	T	.	.	AF=0.017;DP=117;FR=R;LCR=
403	cp	115956	.	G	T	.	.	AF=0.0167;DP=119;FR=R;LCR=
404	cp	116186	.	G	T	.	.	AF=0.0162;DP=123;FR=R;LCR=
405	cp	116406	.	G	T	.	.	AF=0.0146;DP=136;FR=R;LCR=
406	cp	116975	.	C	A	.	.	AF=0.982;DP=111;FR=R;LCR=
407	cp	117009	.	A	G	.	.	AF=0.973;DP=111;FR=R;LCR=
408	cp	117047	.	C	T,G	.	.	AF=0.0171,;DP=116;FR=R;LCR=
409	cp	132064	.	T	TTGATTG	.	.	AF=0.0101;DP=198;FR=R;LCR=
410	cp	132117	.	T	G	.	.	AF=0.971;DP=69;FR=R;LCR=SNR
411	cp	132323	.	G	T	.	.	AF=0.0262;DP=76;FR=R;LCR=SNR(T)
412	cp	132733	.	C	A	.	.	AF=0.018;DP=111;FR=R;LCR=
413	cp	132834	.	T	G,A	.	.	AF=0.988,;DP=167;FR=R;LCR=
414	cp	132836	.	G	C	.	.	AF=0.0108;DP=184;FR=R;LCR=
415	cp	132894	.	AGA	A	.	.	AF=1;DP=195;FR=R;LCR=
416	cp	133034	.	T	TCTGTTTCATCA	.	.	AF=0.976;DP=168;FR=R;LCR=
417	cp	133069	.	A	G,T	.	.	AF=0.988,;DP=258;FR=R;LCR=
418	cp	133077	.	G	T	.	.	AF=0.0123;DP=243;FR=R;LCR=
419	cp	133078	.	A	T	.	.	AF=0.0123;DP=243;FR=R;LCR=
420	cp	133678	.	G	T	.	.	AF=0.0118;DP=255;FR=R;LCR=
421	cp	133893	.	G	A	.	.	AF=0.0165;DP=120;FR=R;LCR=
422	cp	133938	.	A	G	.	.	AF=0.0192;DP=104;FR=R;LCR=
423	cp	134177	.	C	A	.	.	AF=0.0193;DP=155;FR=R;LCR=
424	cp	134440	.	T	C	.	.	AF=0.0152;DP=131;FR=R;LCR=
425	cp	134612	.	G	T	.	.	AF=0.0165;DP=120;FR=R;LCR=
426	cp	134726	.	A	T	.	.	AF=0.0101;DP=297;FR=R;LCR=SNR
427	cp	135016	.	C	A	.	.	AF=0.0186;DP=107;FR=R;LCR=SNR(T)
428	cp	137723	.	G	T	.	.	AF=0.0175;DP=114;FR=R;LCR=
429	cp	137835	.	C	G	.	.	AF=0.0112;DP=355;FR=R;LCR=
430	cp	137882	.	G	T	.	.	AF=0.0163;DP=122;FR=R;LCR=
431	cp	138049	.	C	A	.	.	AF=0.0144;DP=138;FR=R;LCR=
432	cp	138189	.	T	C	.	.	AF=0.016;DP=125;FR=R;LCR=
433	cp	138594	.	C	A	.	.	AF=0.0105;DP=286;FR=R;LCR=
434	cp	138801	.	G	T	.	.	AF=0.0134;DP=148;FR=R;LCR=
435	cp	139170	.	C	A	.	.	AF=0.0168;DP=118;FR=R;LCR=
436	cp	139329	.	G	T	.	.	AF=0.0191;DP=104;FR=R;LCR=
437	cp	139394	.	G	C	.	.	AF=0.0179;DP=111;FR=R;LCR=
438	cp	139966	.	C	A	.	.	AF=0.0196;DP=101;FR=R;LCR=
439	cp	140338	.	G	T	.	.	AF=0.016;DP=124;FR=R;LCR=
440	cp	140537	.	C	A	.	.	AF=0.0162;DP=123;FR=R;LCR=
441	cp	140612	.	C	A	.	.	AF=0.0155;DP=129;FR=R;LCR=
442	cp	141795	.	G	T	.	.	AF=0.0137;DP=146;FR=R;LCR=
443	cp	141797	.	G	T	.	.	AF=0.0134;DP=148;FR=R;LCR=
444	cp	141938	.	G	T	.	.	AF=0.0173;DP=115;FR=R;LCR=
445	cp	142220	.	G	T	.	.	AF=0.0159;DP=125;FR=R;LCR=
446	cp	142596	.	G	T	.	.	AF=0.0173;DP=115;FR=R;LCR=
447	cp	142656	.	C	A	.	.	AF=0.0159;DP=125;FR=R;LCR=
448	cp	142825	.	G	T	.	.	AF=0.0164;DP=121;FR=R;LCR=
449	cp	142896	.	C	A	.	.	AF=0.0179;DP=111;FR=R;LCR=
450	cp	142958	.	C	A	.	.	AF=0.017;DP=117;FR=R;LCR=

451	cp	143331	.	G	T	.	.	AF=0.0133;DP=150;FR=R;LCR=
452	cp	143399	.	C	A	.	.	AF=0.0179;DP=111;FR=R;LCR=
453	cp	143970	.	A	G,T	.	.	AF=0.977,;DP=131;FR=R;LCR=
454	cp	144378	.	G	T,C	.	.	AF=0.0179,;DP=111;FR=R;LCR=
455	cp	145210	.	A	G	.	.	AF=0.0171;DP=116;FR=R;LCR=
456	cp	145633	.	G	T	.	.	AF=0.0161;DP=124;FR=R;LCR=
457	cp	146971	.	C	T	.	.	AF=0.986;DP=142;FR=R;LCR=SNR
458	cp	146994	.	T	TNAA	.	.	AF=0;DP=256;FR=R;LCR=
459	cp	146994	.	T	TNAA	.	.	AF=0;DP=256;FR=R;LCR=
460	cp	147040	.	G	T	.	.	AF=0.0167;DP=119;FR=R;LCR=
461	cp	147080	.	AGATTATAAGAT	A	.	.	AF=0.984;DP=258;FR=R;LCR=
462	cp	147548	.	G	T,C	.	.	AF=0.017,;DP=117;FR=R;LCR=
463	cp	147650	.	G	T	.	.	AF=0.016;DP=125;FR=R;LCR=
464	cp	147685	.	C	A	.	.	AF=0.0154;DP=129;FR=R;LCR=
465	cp	148071	.	G	T	.	.	AF=0.0152;DP=131;FR=R;LCR=
466	cp	148215	.	G	T	.	.	AF=0.983;DP=119;FR=R;LCR=
467	cp	148334	.	G	T	.	.	AF=0.013;DP=307;FR=R;LCR=
468	cp	148385	.	C	A	.	.	AF=0.0179;DP=111;FR=R;LCR=
469	cp	148535	.	C	A	.	.	AF=0.0168;DP=118;FR=R;LCR=
470	cp	148652	.	G	T	.	.	AF=0.0153;DP=130;FR=R;LCR=
471	cp	148936	.	C	A	.	.	AF=0.0174;DP=114;FR=R;LCR=
472	cp	148954	.	C	A	.	.	AF=0.017;DP=117;FR=R;LCR=
473	cp	149262	.	G	T	.	.	AF=0.0132;DP=151;FR=R;LCR=
474	cp	149298	.					
475		G,A	.	.	AF=0.976,;DP=123;FR=R;LCR=			
476	cp	149486	.	TTCAGAAT	T	.	.	AF=0.0121;DP=248;FR=R;LCR=
477	cp	149499	.	A	T	.	.	AF=0.0163;DP=123;FR=R;LCR=
478	cp	149893	.	T	G	.	.	AF=0.0186;DP=107;FR=R;LCR=
479	cp	150167	.	G	T	.	.	AF=0.0149;DP=134;FR=R;LCR=
480	cp	150347	.	G	T	.	.	AF=0.0207;DP=144;FR=R;LCR=
481	cp	150356	.	T	C	.	.	AF=0.0155;DP=128;FR=R;LCR=
482	cp	150415	.	T	C,A	.	.	AF=0.983,;DP=114;FR=R;LCR=
483	cp	150946	.	C	A	.	.	AF=0.0149;DP=134;FR=R;LCR=
484	cp	151122	.	C	A	.	.	AF=0.0139;DP=144;FR=R;LCR=
485	cp	151182	.	C	A	.	.	AF=0.016;DP=124;FR=R;LCR=
486	cp	151517	.	C	A	.	.	AF=0.0181;DP=110;FR=R;LCR=
487	cp	152058	.	C	A	.	.	AF=0.0151;DP=330;FR=R;LCR=SNR
488	cp	152228	.	G	A	.	.	AF=0.0156;DP=128;FR=R;LCR=
489	cp	152233	.	C	T	.	.	AF=0.0163;DP=122;FR=R;LCR=
490	cp	152239	.	G	A,T	.	.	AF=0.0166,;DP=120;FR=R;LCR=
491	cp	152370	.	G	A	.	.	AF=0.0181;DP=110;FR=R;LCR=
492	cp	152685	.	C	A	.	.	AF=0.0148;DP=135;FR=R;LCR=
493	cp	152798	.	A	G	.	.	AF=0.0134;DP=149;FR=R;LCR=
494	cp	152823	.	C	A	.	.	AF=0.015;DP=133;FR=R;LCR=
495	cp	153092	.	C	A	.	.	AF=0.0138;DP=145;FR=R;LCR=
496	cp	153149	.	G	T	.	.	AF=0.0129;DP=154;FR=R;LCR=
497	cp	153202	.	A	G,T	.	.	AF=0.987,;DP=149;FR=R;LCR=
498	cp	153326	.	T	C	.	.	AF=0.0161;DP=123;FR=R;LCR=
499	cp	153737	.	C	A	.	.	AF=0.0131;DP=153;FR=R;LCR=
500	cp	154239	.	G	T	.	.	AF=0.0161;DP=124;FR=R;LCR=
501	cp	154388	.	G	T	.	.	AF=0.0139;DP=144;FR=R;LCR=
502	cp	154429	.	G	A	.	.	AF=0.0138;DP=145;FR=R;LCR=
503	cp	154989	.	G	T	.	.	AF=0.0165;DP=120;FR=R;LCR=
504	cp	155386	.	C	A	.	.	AF=0.0149;DP=134;FR=R;LCR=
505	cp	155387	.	G	T	.	.	AF=0.0151;DP=132;FR=R;LCR=
506	cp	155395	.	G	T	.	.	AF=0.0161;DP=123;FR=R;LCR=
507	cp	155736	.	C	A	.	.	AF=0.0179;DP=111;FR=R;LCR=
508	cp	155808	.	C	A	.	.	AF=0.0139;DP=143;FR=R;LCR=
509	cp	155858	.	G	A	.	.	AF=0.0132;DP=151;FR=R;LCR=
510	cp	155883	.	G	A	.	.	AF=0.013;DP=153;FR=R;LCR=

511	cp	156055	.	C	A,T	.	.	AF=0.0174,;DP=115;FR=R;LCR=
512	cp	156280	.	C	A	.	.	AF=0.0174;DP=114;FR=R;LCR=SNR
513	cp	156893	.	A	G,T	.	.	AF=0.983,;DP=118;FR=R;LCR=
514	cp	156952	.	C	A	.	.	AF=0.015;DP=133;FR=R;LCR=
515	cp	157141	.	G	T	.	.	AF=0.0177;DP=112;FR=R;LCR=
516	cp	157706	.	T	G,C	.	.	AF=0.974,;DP=116;FR=R;LCR=
517	cp	157904	.	T	C,A	.	.	AF=0.986,;DP=294;FR=R;LCR=SNR
518	cp	157943	.	A	T	.	.	AF=0.0168;DP=119;FR=R;LCR=
519	cp	157954	.	C	A	.	.	AF=0.0244;DP=122;FR=R;LCR=
520	cp	157981	.	G	T	.	.	AF=0.015;DP=133;FR=R;LCR=
521	cp	158455	.	A	G,T	.	.	AF=0.98,;DP=148;FR=R;LCR=
522	cp	158772	.	C	A	.	.	AF=0.0149;DP=133;FR=R;LCR=
523	cp	159533	.	G	T	.	.	AF=0.0137;DP=418;FR=F;LCR=
524	cp	159762	.	G	T	.	.	AF=0.0168;DP=119;FR=R;LCR=
525	cp	160254	.	C	T	.	.	AF=0.0155;DP=193;FR=F;LCR=
526	cp	160594	.	G	A	.	.	AF=0.0157;DP=254;FR=F;LCR=
527	cp	160684	.	C	A	.	.	AF=0.0188;DP=106;FR=R;LCR=
528	cp	160713	.	C	A	.	.	AF=0.0188;DP=106;FR=R;LCR=
529	cp	161320	.	G	T	.	.	AF=0.0109;DP=274;FR=F;LCR=
530	cp	161523	.	A	T	.	.	AF=0.0145;DP=207;FR=F;LCR=
531	cp	161561	.	C	A	.	.	AF=0.0104;DP=192;FR=F;LCR=
532	cp	161741	.	G	A	.	.	AF=0.0117;DP=256;FR=F;LCR=
533	cp	162276	.	CC	C	.	.	AF=0.0109;DP=274;FR=R;LCR=SNR
534	cp	162276	.	CC	C	.	.	AF=0.0109;DP=274;FR=R;LCR=SNR
535	cp	162278	.	C		.	.	AF=;DP=3;FR=R;LCR=SNR
536	cp	162278	.	C		.	.	AF=;DP=3;FR=R;LCR=SNR
537	cp	162307	.	T	C	.	.	AF=0.0107;DP=186;FR=F;LCR=SNR
538	cp	162361	.	G	T	.	.	AF=0.0112;DP=179;FR=F;LCR=
539	cp	162365	.	A	T,C	.	.	AF=0.99,;DP=199;FR=F;LCR=
540	cp	162387	.	G	T	.	.	AF=0.0132;DP=151;FR=F;LCR=SNR
541	cp	162426	.	T	A	.	.	AF=0.0208;DP=144;FR=F;LCR=SNR
542	cp	162481	.	A	G	.	.	AF=0.0103;DP=193;FR=F;LCR=

Figure S12. The original and full-length gel electrophoresis results of the amplification of DNA barcodes using designed primers.
This is the original and uncropped gel electrophoresis plot for the cropped image shown in Figure 7. Lane M was the marker of DL1000. The upper lanes from left to right corresponded to products amplified from the individual 1 and individual 2 of *A. galanga* (Alga), *A. hainanensis* (Alha), *A. nigra* (Alni), *A. officinarum* (Alof), and *A. oxyphylla* (Alox) by primer Alpp, respectively. The lower lanes from left to right corresponded to products amplificated from the individual 2 and individual 1 of Alga, Alha, Alni, Alof, and Alox by primer Alpr, respectively.

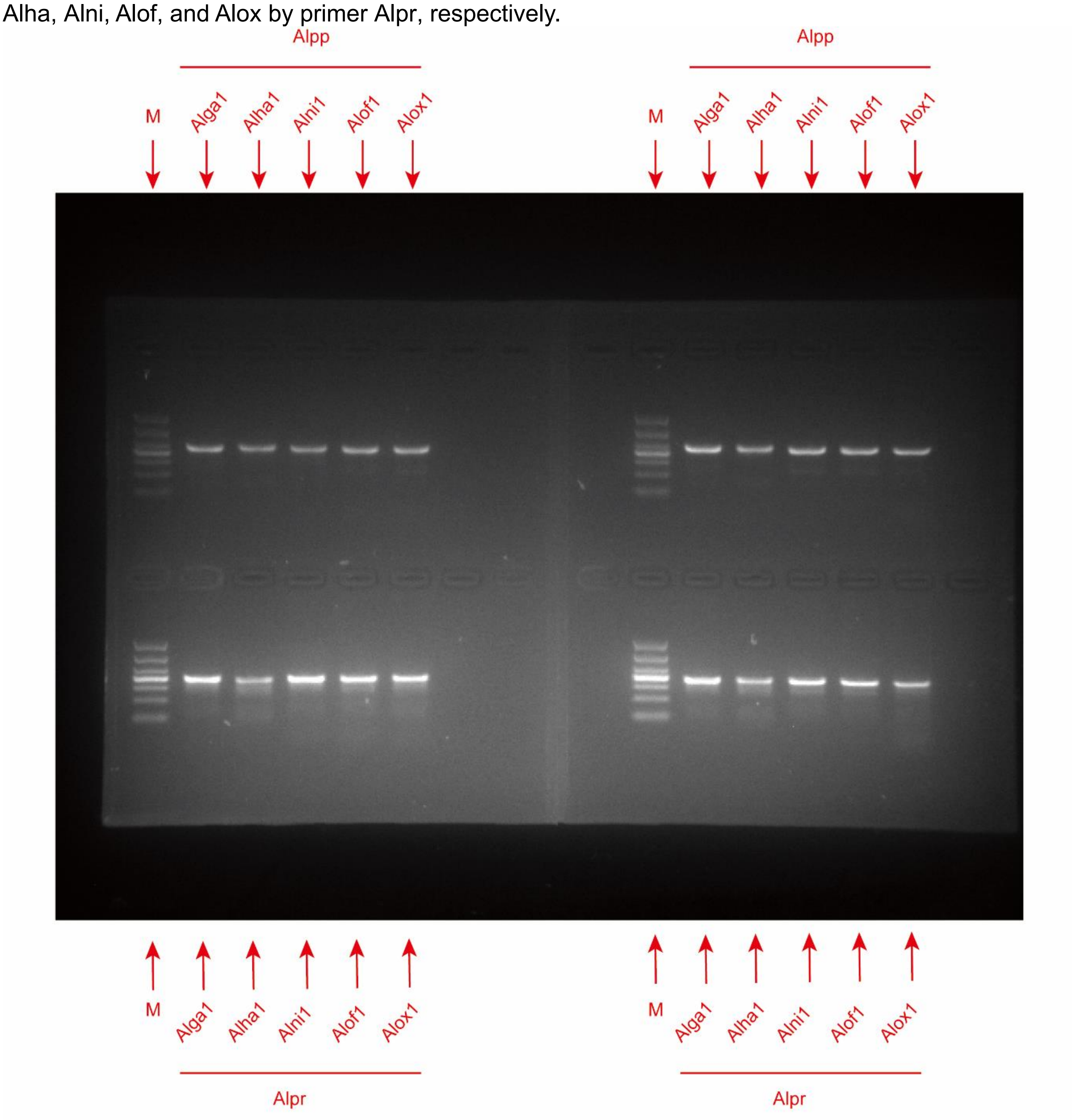

Figure S13. The alignment of amplicons produced by designed Alpp primers.

The ID of each sequence is shown on the left side of each panel. The ID is the concatenation of species name, plant individual id, and primer name. The rightmost number in each column represents the position of the base at that position in the amplicon. The SNPs and Indel are shown in red squares. The nucleotides identical across all plastomes are shaded in black, whereas those conserved in 60% of the sequences are shaded in gray. *Alpinia galanga*: Alga; *A. hainanensis*: Alha; *A. nigra*: Alni; *A. officinarum*: Alof; *A. oxyphylla*: Alox. Arabic numerals represent different individuals.

```
Alga1_Alpp : ---------------------------aaaagtca-acga--tcacacaggt-aatgtag :  29
Alga2_Alpp : ------------------------t--caaaacat-cgaa--tcacacaggtaaatgtag :  31
Alga3_Alpp : ------------------------gcaaagaacga-tcga--atccacaggt-aatgtag :  32
Alga4_Alpp : ------------------------a--cagactaatcgca--tcacacaggtaaatgtag :  32
Alga6_Alpp : -----------------------------gcgaca-atga--atccacaggt-aatgtag :  27
Alga7_Alpp : ------------------------a--cacaggga-acga--atccacaggtaaatgtag :  31
Alga8_Alpp : ------------------------gaaaaggacaa--cga--atccacaggtaaatgtag :  32
Alga0_Alpp : agaacagttggccttcgattattttggatcgatcg-aaat--acacacaggtaaatgtag :  57
Alha1_Alpp : a---------------------------aagacga-acga--taccacaggtaaatgtag :  30
Alha2_Alpp : c---------------------------aagacta-acga--atccacaggtaaatgtag :  30
Alha3_Alpp : caaaacatgcat------------------------cga----tccacaggtaaatgtag :  32
Alha6_Alpp : a----------------------------cagaca-acga--taacacaggt-aatgtag :  28
Alha7_Alpp : ccaaaaggccaa------------------------cgaa--tacaacaggtaaatgtag :  34
Alha8_Alpp : acaagcatcga--------------------------------tccacaggt-aatgtag :  27
Alha0_Alpp : agaacagttggccttcgattattttggatcgatcg-aaat--acacacaggtaaatgtag :  57
Alni1_Alpp : ---------------------------caagccat-cgaa--tcacacaggt-aatgtag :  29
Alni2_Alpp : ------------------------c--aaatacat-cgaa--tcacacaggt-aatgtag :  30
Alni3_Alpp : ------------------------c--acaagact-atcg--atccacaggt-aatgtag :  30
Alni4_Alpp : ------------------------g--aaagacat-cgaa--taccacaggt-aatgtag :  30
Alni6_Alpp : ------------------------a--aaagacca-tcga--tcacacaggt-aatgtag :  30
Alni7_Alpp : ------------------------g--cagaacca-acga--tcacacaggtaaatgtag :  31
Alni8_Alpp : ------------------------ccaatgaaggc-atcg--atccacaggt-aatgtag :  32
Alni0_Alpp : agaacagttggccttcgattattttggatcgatcg-aaat--acacacaggtaaatgtag :  57
Alof1_Alpp : ---------------------------cagaacaa-tcga--tcacacaggtaaatgtag :  30
Alof2_Alpp : ----------------------------aaaggca-acga--taccacaggt-aatgtag :  28
Alof3_Alpp : ---------------------------aaagaaaa-tcga--atccacaggt-aatgtag :  29
Alof4_Alpp : ---------------------------gcatacaa-tcga--taacacaggt-aatgtag :  29
Alof6_Alpp : ---------------------------gaagacca-tcg---atccacaggt-aatgtag :  28
Alof7_Alpp : ---------------------------acagacca-acgaatacacacaggt-aatgtag :  31
Alof8_Alpp : --------------------------gaaagtcca-tcga--taacacaggt-aatgtag :  30
Alof0_Alpp : agaacagttggccttcgattattttggatcgatcg-aaat--acacacaggtaaatgtag :  57
Alox1_Alpp : ---------------------------aagaacaa-acga--atccacaggt-aatgtag :  29
Alox2_Alpp : g-------------------------aaaggcaaa-cgaa--tcacacaggtaaatgtag :  32
Alox3_Alpp : --------------------------gaagaacaa-tcga--tcacacaggt-aatgtag :  30
Alox6_Alpp : g-------------------------aaggaccca-acga--atccacaggt-aatgtag :  31
Alox7_Alpp : --------------------------aaagggcaa-tcga--tcacacaggtaaatgtag :  31
Alox0_Alpp : agaacagttggccttcgattattttggatcgatcg-aaat--acacacaggtaaatgtag :  57

Alga1_Alpp : ccaaaaaaaagaattgggctgctattttgatgtactatttagatatatatctaatctatg :  89
Alga2_Alpp : ccaaaaaaaagaattgggctgctattttgatgtactatttagatatatatctaatctatg :  91
Alga3_Alpp : ccaaaaaaaagaattgggctgctattttgatgtactatttagatatatatctaatctatg :  92
Alga4_Alpp : ccaaaaaaaagaattgggctgctattttgatgtactatttagatatatatctaatctatg :  92
Alga6_Alpp : ccaaaaaaaagaattgggctgctattttgatgtactatttagatatatatctaatctatg :  87
Alga7_Alpp : ccaaaaaaaagaattgggctgctattttgatgtactatttagatatatatctaatctatg :  91
Alga8_Alpp : ccaaaaaaaagaattgggctgctattttgatgtactatttagatatatatctaatctatg :  92
Alga0_Alpp : ccaaaaaaaagaattgggctgctattttgatgtactatttagatatatatctaatctatg : 117
Alha1_Alpp : ccaaaaaaaagaattgggctgctattttgatgtattatttagatatacatctaatccatg :  90
Alha2_Alpp : ccaaaaaaaagaattgggctgctattttgatgtattatttagatatacatctaatccatg :  90
Alha3_Alpp : ccaaaaaaaagaattgggctgctattttgatgtattatttagatatacatctaatccatg :  92
Alha6_Alpp : ccaaaaaaaagaattgggctgctattttgatgtattatttagatatacatctaatccatg :  88
Alha7_Alpp : ccaaaaaaaagaattgggctgctattttgatgtattatttagatatacatctaatccatg :  94
Alha8_Alpp : ccaaaaaaaagaattgggctgctattttgatgtattatttagatatacatctaatccatg :  87
Alha0_Alpp : ccaaaaaaaagaattgggctgctattttgatgtattatttagatatacatctaatccatg : 117
Alni1_Alpp : ccaaaaaaaagaattgggctgctattttgatgtactatttagatatatatctaatctatg :  89
Alni2_Alpp : ccaaaaaaaagaattgggctgctattttgatgtactatttagatatatatctaatctatg :  90
Alni3_Alpp : ccaaaaaaaagaattgggctgctattttgatgtactatttagatatatatctaatctatg :  90
Alni4_Alpp : ccaaaaaaaagaattgggctgctattttgatgtactatttagatatatatctaatctatg :  90
Alni6_Alpp : ccaaaaaaaagaattgggctgctattttgatgtactatttagatatatatctaatctatg :  90
Alni7_Alpp : ccaaaaaaaagaattgggctgctattttgatgtactatttagatatatatctaatctatg :  91
Alni8_Alpp : ccaaaaaaaagaattgggctgctattttgatgtactatttagatatatatctaatctatg :  92
Alni0_Alpp : ccaaaaaaaagaattgggctgctattttgatgtactatttagatatatatctaatctatg : 117
Alof1_Alpp : ccaaaaaaaagaattgggctgctattttgatgtactatttagatatacatctaatccatg :  90
```

```
Alof2_Alpp : ccaaaaaaaagaattgggctgctattttgatgtactatttagatatacatctaatccatg :  88
Alof3_Alpp : ccaaaaaaaagaattgggctgctattttgatgtactatttagatatacatctaatccatg :  89
Alof4_Alpp : ccaaaaaaaagaattgggctgctattttgatgtactatttagatatacatctaatccatg :  89
Alof6_Alpp : ccaaaaaaaagaattgggctgctattttgatgtactatttagatatacatctaatccatg :  88
Alof7_Alpp : ccaaaaaaaagaattgggctgctattttgatgtactatttagatatacatctaatccatg :  91
Alof8_Alpp : ccaaaaaaaagaattgggctgctattttgatgtactatttagatatacatctaatccatg :  90
Alof0_Alpp : ccaaaaaaaagaattgggctgctattttgatgtactatttagatatacatctaatccatg : 117
Alox1_Alpp : ccaaaaaaaagaattgggctgctattttgatgtactatttagatatacatctaatccatg :  89
Alox2_Alpp : ccaaaaaaaagaattgggctgctattttgatgtactatttagatatacatctaatccatg :  92
Alox3_Alpp : ccaaaaaaaagaattgggctgctattttgatgtactatttagatatacatctaatccatg :  90
Alox6_Alpp : ccaaaaaaaagaattgggctgctattttgatgtactatttagatatacatctaatccatg :  91
Alox7_Alpp : ccaaaaaaaagaattgggctgctattttgatgtactatttagatatacatctaatccatg :  91
Alox0_Alpp : ccaaaaaaaagaattgggctgctattttgatgtactatttagatatacatctaatccatg : 117

Alga1_Alpp : tacatacatattgtatattgatctagatatgggc---ttaaatagtacatcaaaatagca : 146
Alga2_Alpp : tacatacatattgtatattgatctagatatgggc---ttaaataggacgtcaaaatagca : 148
Alga3_Alpp : tacatacatattgtatattgatctagatatgggc---ttaaataggacatcaaaatagca : 149
Alga4_Alpp : tacatacatattgtatattgatctagatatgggc---ttaaatagtacatcaaaatagca : 149
Alga6_Alpp : tacatacatattgtatattgatctagatatgtat---ctaaataggacgtcaaaatagca : 144
Alga7_Alpp : tacatacatattgtatattgatctagatatgtat---ctaaataggacatcaaaatagca : 148
Alga8_Alpp : tacatacatattgtatattgatctagatatgggc---ttaaataggacgtcaaaatagca : 149
Alga0_Alpp : tacatacatattgtatattgatctagatatgggc-tttttttta-------------tag : 163
Alha1_Alpp : tacatacatattgtatattgatctagatatgggcttttttttta-------------tag : 137
Alha2_Alpp : tacatacatattgtatattgatctagatatgggcttttttttta-------------tag : 137
Alha3_Alpp : tacatacatattgtatattgatctagatatgggcttttttttta-------------tag : 139
Alha6_Alpp : tacatacatattgtatattgatctagatatgggcttttttttta-------------tag : 135
Alha7_Alpp : tacatacatattgtatattgatctagatatgggcttttttttta-------------tag : 141
Alha8_Alpp : tacatacatattgtatattgatctagatatgggcttttttttta-------------tag : 134
Alha0_Alpp : tacatacatattgtatattgatctagatatgggcttttttttta-------------tag : 164
Alni1_Alpp : tacatacatattatatattgatctagataggggc--tttttttt-------------ata : 134
Alni2_Alpp : tacatacatattatatattgatctagataggggc--tttttttt-------------ata : 135
Alni3_Alpp : tacatacatattatatattgatctagataggggc--tttttttg-------------gta : 135
Alni4_Alpp : tacatacatattatatattgatctagataggggc--tttttttt-------------ata : 135
Alni6_Alpp : tacatacatattatatattgatctagatatgggc---tttttta-------------gca : 134
Alni7_Alpp : tacatacatattatatattgatctagatatgggc---ttttttg-------------ggg : 135
Alni8_Alpp : tacatacatattatatattgatctagatatgggc--tttttttg-------------gta : 137
Alni0_Alpp : tacatacatattatatattgatctagatatgggctttttttt---------------ata : 162
Alof1_Alpp : tacatacatattgtatattgatctagatatgggc---tttttta-------------tag : 134
Alof2_Alpp : tacatacatattgtatattgatctagatatgggc---tttttta-------------tag : 132
Alof3_Alpp : tacatacatattgtatattgatctagatatgggc---tttttta-------------tag : 133
Alof4_Alpp : tacatacatattgtatattgatctagatatgggc---tttttta-------------tag : 133
Alof6_Alpp : tacatacatattgtatattgatctagatatgggc---tttttta-------------tag : 132
Alof7_Alpp : tacatacatattgtatattgatctagatatgggc---tttttta-------------tag : 135
Alof8_Alpp : tacatacatattgtatattgatctagatatgggc---tttttta-------------tag : 134
Alof0_Alpp : tacatacatattgtatattgatctagatatgggc---tttttta-------------tag : 161
Alox1_Alpp : tacatacatattgtatattgatctagatatgggc---tttttta-------------tag : 133
Alox2_Alpp : tacatacatattgtatattgatctagatatgggc---tttttta-------------tag : 136
Alox3_Alpp : tacatacatattgtatattgatctagatatgggc---tttttta-------------tag : 134
Alox6_Alpp : tacatacatattgtatattgatctagatatgggc---tttttta-------------tag : 135
Alox7_Alpp : tacatacatattgtatattgatctagatatgggc---tttttta-------------tag : 135
Alox0_Alpp : tacatacatattgtatattgatctagatatgggc---tttttta-------------tag : 161

Alga1_Alpp : gcccaattctttttttttgggtacatttacctgtgtgtatttcgatcgatccaaaataatc : 206
Alga2_Alpp : gcccaattctttttttttgggtacatttacctgtgtgtatttcgatcgatccaaaataatg : 208
Alga3_Alpp : tcccaattctttttttttgggtacatttacctttgtgtatttcgatcgatccaaaataatg : 209
Alga4_Alpp : tcccaattctttttttttgggtacatttacctttgtgtatttcgatcgatccaaaataatc : 209
Alga6_Alpp : gcccaattctttttttttgggtacatttacctgtgtgtatttcgatcgatccaaaataatg : 204
Alga7_Alpp : gcccaattctttttttttgggtacatttacctgtgtgtatttcgatcgatccaaaataatc : 208
Alga8_Alpp : tcccaattctttttttttgggtacatttacctttgtgtatttcgatcgatccaaaataatg : 209
Alga0_Alpp : gaaaaagcccatatctagatcaatatataatacaactctctatgaaaataatgaatatga : 223
Alha1_Alpp : gaaaaagtctatatctagatcaatatataatacaactctctatgaaaataatgaatatga : 197
Alha2_Alpp : gaaaaagtctatatctagatcaatatataatacaactctctatgaaaataatgaatatga : 197
Alha3_Alpp : gaaaaagtctatatctagatcaatatataatacaactctctatgaaaataatgaatatga : 199
Alha6_Alpp : gaaaaagtctatatctagatcaatatataatacaactctctatgaaaataatgaatatga : 195
Alha7_Alpp : gaaaaagtctatatctagatcaatatataatacaactctctatgaaaataatgaatatga : 201
Alha8_Alpp : gaaaaagtctatatctagatcaatatataatacaactctctatgaaaataatgaatatga : 194
Alha0_Alpp : gaaaaagtctatatctagatcaatatataatacaactctctatgaaaataatgaatatga : 224
Alni1_Alpp : ggaaaagcccatatctaggtcaatatataatgcgagtctccaggaagataatgaatatga : 194
Alni2_Alpp : ggaaaagcccatatcttggtcaatatataatgcgtgtctcccggaagataataaatatga : 195
Alni3_Alpp : ggaaaagcccatatctaggtcaatatataatgcgtgtctccaggaagataataaatatga : 195
Alni4_Alpp : ggaaaagcccatatctaggtcaatatataatgcgagtctccaggaagataatgaatatga : 195
Alni6_Alpp : gcccaattctttttttttggctacatttacgtctgtgtatttcggtgggtccaaaataatc : 194
```

```
Alni7_Alpp : ggaaaagccttgtctaggtcaatatttagtgcgtgtctcccggtgggtaatgaataaaga : 195
Alni8_Alpp : ggaaaagcccttatctagatcaatatttagtgcgtgtctccaggaagataatgaatatga : 197
Alni0_Alpp : ggaaaagcccatatctagatcaatatataatacaactctccatgaaaataatgaatatga : 222
Alof1_Alpp : gaaaaagtctatatctagatcaatatataatacaactctctatgaaaataatgaatatga : 194
Alof2_Alpp : gaaaaagtctatatctagatcaatatataatacaactctctatgaaaataatgaatatga : 192
Alof3_Alpp : gaaaaagtctatatctagatcaatatataatacaactctctatgaaaataatgaatatga : 193
Alof4_Alpp : gaaaaagtctatatctagatcaatatataatacaactctctatgaaaataatgaatatga : 193
Alof6_Alpp : gaaaaagtctatatctagatcaatatataatacaactctctatgaaaataatgaatatga : 192
Alof7_Alpp : gaaaaagtctatatctagatcaatatataatacaactctctatgaaaataatgaatatga : 195
Alof8_Alpp : gaaaaagtctatatctagatcaatatataatacaactctctatgaaaataatgaatatga : 194
Alof0_Alpp : gaaaaagtctatatctagatcaatatataatacaactctctatgaaaataatgaatatga : 221
Alox1_Alpp : gaaaaagtctatatctagatcaatatataatacaactctctatgaaaataatgaatatga : 193
Alox2_Alpp : gaaaaagtctatatctagatcaatatataatacaactctctatgaaaataatgaatatga : 196
Alox3_Alpp : gaaaaagtctatatctagatcaatatataatacaactctctatgaaaataatgaatatga : 194
Alox6_Alpp : gaaaaagtctatatctagatcaatatataatacaactctctatgaaaataatgaatatga : 195
Alox7_Alpp : gaaaaagtctatatctagatcaatatataatacaactctctatgaaaataatgaatatga : 195
Alox0_Alpp : gaaaaagtctatatctagatcaatatataatacaactctctatgaaaataatgaatatga : 221

Alga1_Alpp : gaaggccaactgttcta------------------------------------------- : 223
Alga2_Alpp : gaaggccaactgttcta-----------tataccatactatctaggcttagatagtacta : 257
Alga3_Alpp : gaaggccaactgttcta-----------tataccatactatctaggcttagatagtacta : 258
Alga4_Alpp : gaaggccaactgttcta-----------tataccatactatctaggcttagatagtacta : 258
Alga6_Alpp : gaaggccaactgttcta------------------------------------------- : 221
Alga7_Alpp : gaagaccaactgttcta------------------------------------------- : 225
Alga8_Alpp : gaagaccaactgttcta-----------tataccatactatctaggcttagatagtacta : 258
Alga0_Alpp : taatatatactatataatagtagataactataccatactatctaggcttagatagtacta : 283
Alha1_Alpp : taatatatactatataatagtggataactataccatactatccaggcttagatagtacta : 257
Alha2_Alpp : taatatatactatataatagtggataactataccatactatccaggcttagatagtacta : 257
Alha3_Alpp : taatatatactatataatagtggataactataccatactatccaggcttagatagtacta : 259
Alha6_Alpp : taatatatactatataatagtggataactataccatactatccaggcttagatagtacta : 255
Alha7_Alpp : taatatatactatataatagtggataactataccatactatccaggcttagatagtacta : 261
Alha8_Alpp : taatatatactatataatagtggataactataccatactatccaggcttagatagtacta : 254
Alha0_Alpp : taatatatactatataatagtggataactataccatactatccaggcttagatagtacta : 284
Alni1_Alpp : gaaggtatactgtataatagtagataactataccatactatctaggcttagatagtacta : 254
Alni2_Alpp : gaaggtatactggactatagaagataactataccatactatctaggcttagatagtacta : 255
Alni3_Alpp : gaaggtatactgtataatagtagataactataccatactatctaggcttagatagtacta : 255
Alni4_Alpp : gaaggtatactgtataatagtagataactataccatactatctaggcttagatagtacta : 255
Alni6_Alpp : gaaggccaactgttctatataccgtaccaacaccgtactaacaaggcttacacagaactt : 254
Alni7_Alpp : gaaggtatactgtataatagaagataactataccatactatctaggcttagatagtacta : 255
Alni8_Alpp : gaaggtatactgtataatagtagataactataccatactatctaggcttagatagtacta : 257
Alni0_Alpp : taatatatactatataatagtagataactataccatactatctaggcttagatagtacta : 282
Alof1_Alpp : taatatatacta----------gataactataccatactatctaggcttagatagtac-- : 242
Alof2_Alpp : taatatatacta----------gataactataccatactatctaggcttagatagtac-- : 240
Alof3_Alpp : taatatatacta----------gataactataccatactatctaggcttagatagtac-- : 241
Alof4_Alpp : taatatatacta----------gataactataccatactatctaggcttagatagtac-- : 241
Alof6_Alpp : taatatatacta----------gataactataccatactatctaggcttagatagtac-- : 240
Alof7_Alpp : taatatatacta----------gataactataccatactatctaggcttagatagtac-- : 243
Alof8_Alpp : taatatatacta----------gataactataccatactatctaggcttagatagtac-- : 242
Alof0_Alpp : taatatatacta----------gataactataccatactatctaggcttagatagtac-- : 269
Alox1_Alpp : taatatatacta----------gataactataccatactatctaggcttagatagtac-- : 241
Alox2_Alpp : taatatatacta----------gataactataccatactatctaggcttagatagtac-- : 244
Alox3_Alpp : taatatatacta----------gataactataccatactatctaggcttagatagtac-- : 242
Alox6_Alpp : taatatatacta----------gataactataccatactatctaggcttagatagtac-- : 243
Alox7_Alpp : taatatatacta----------gataactataccatactatctaggcttagatagtac-- : 243
Alox0_Alpp : taatatatacta----------gataactataccatactatctaggcttagatagtac-- : 269

Alga1_Alpp : ------------------------------------------------------------ :   -
Alga2_Alpp : tctcagatacttattatctcagatacttatgattccagccagatcatttctttcgtttaa : 317
Alga3_Alpp : tctcagatacttattatctcagatacttatgattccagccagatcatttctttcgtttaa : 318
Alga4_Alpp : tctcagatacttattatctcagatacttatgattccagccagatcatttctttcgtttaa : 318
Alga6_Alpp : ------------------------------------------------------------ :   -
Alga7_Alpp : ------------------------------------------------------------ :   -
Alga8_Alpp : tctcagatacttattatctcagatacttatgattccagccagatcatttctttcgtttaa : 318
Alga0_Alpp : tctcagatacttattatctcagatacttatgattccagccagatcatttctttcgtttaa : 343
Alha1_Alpp : tctcagatacttattatctcagatatttatgattccagccagatcatttctttcgtttaa : 317
Alha2_Alpp : tctcagatacttattatctcagatatttatgattccagccagatcatttctttcgtttaa : 317
Alha3_Alpp : tctcagatacttattatctcagatatttatgattccagccagatcatttctttcgtttaa : 319
Alha6_Alpp : tctcagatacttattatctcagatatttatgattccagccagatcatttctttcgtttaa : 315
Alha7_Alpp : tctcagatacttattatctcagatatttatgattccagccagatcatttctttcgtttaa : 321
Alha8_Alpp : tctcagatacttattatctcagatatttatgattccagccagatcatttctttcgtttaa : 314
Alha0_Alpp : tctcagatacttattatctcagatatttatgattccagccagatcatttctttcgtttaa : 344
Alni1_Alpp : tctaagatacttattatctcagatacttattattccagccggatcatttctttcgtttaa : 314
```

```
Alni2_Alpp : tctaagatacttattatctcagatacttattattccagccggatcatttctttcgtttaa : 315
Alni3_Alpp : tctaagatacttattatctcagatacttattattccagccggatcatttctttcgtttaa : 315
Alni4_Alpp : tctaagatacttattatctcagatacttattattccagccggatcatttctttcgtttaa : 315
Alni6_Alpp : tctaataccctcattatctcatatacttattattacttccggatcgtttctttctccttt : 314
Alni7_Alpp : tctaagatacttattatctcagatacttattattccagccggatcatttctttcgtttaa : 315
Alni8_Alpp : tctaagatacttattatctcagatacttattattccagccggatcatttctttcgtttaa : 317
Alni0_Alpp : tctaagatacttattatctcagatacttattattccagccggatcatttctttcgtttaa : 342
Alof1_Alpp : --------------tatctcagatacttatgattccagccagatcatttctttcgtttaa : 288
Alof2_Alpp : --------------tatctcagatacttatgattccagccagatcatttctttcgtttaa : 286
Alof3_Alpp : --------------tatctcagatacttatgattccagccagatcatttctttcgtttaa : 287
Alof4_Alpp : --------------tatctcagatacttatgattccagccagatcatttctttcgtttaa : 287
Alof6_Alpp : --------------tatctcagatacttatgattccagccagatcatttctttcgtttaa : 286
Alof7_Alpp : --------------tatctcagatacttatgattccagccagatcatttctttcgtttaa : 289
Alof8_Alpp : --------------tatctcagatacttatgattccagccagatcatttctttcgtttaa : 288
Alof0_Alpp : --------------tatctcagatacttatgattccagccagatcatttctttcgtttaa : 315
Alox1_Alpp : --------------tatctcagatacttatgattccagccagatcatttctttcgtttaa : 287
Alox2_Alpp : --------------tatctcagatacttatgattccagccagatcatttctttcgtttaa : 290
Alox3_Alpp : --------------tatctcagatacttatgattccagccagatcatttctttcgtttaa : 288
Alox6_Alpp : --------------tatctcagatacttatgattccagccagatcatttctttcgtttaa : 289
Alox7_Alpp : --------------tatctcagatacttatgattccagccagatcatttctttcgtttaa : 289
Alox0_Alpp : --------------tatctcagatacttatgattccagccagatcatttctttcgtttaa : 315

Alga1_Alpp : ------------------------------------------------------------ :  -
Alga2_Alpp : gacttaaagt-------------ttgacttcaatcattgataagaactaataattcaagt : 364
Alga3_Alpp : gacttaaagt-------------ttgacttcaatcattgataagaactaataattcaagt : 365
Alga4_Alpp : gacttaaagt-------------ttgacttcaatcattgataagaactaataattcaagt : 365
Alga6_Alpp : ------------------------------------------------------------ :  -
Alga7_Alpp : ------------------------------------------------------------ :  -
Alga8_Alpp : gacttaaagt-------------ttgacttcaatcattgataagaactaataattcaagt : 365
Alga0_Alpp : gacttaaagtttga-------------cttcaatcattgataagaactaataattcaagt : 390
Alha1_Alpp : gacttgaagtttgaatccctttcctttcttcaatcattgataagaactaataattcaagt : 377
Alha2_Alpp : gacttgaagtttgaatccctttcctttcttcaatcattgataagaactaataattcaagt : 377
Alha3_Alpp : gacttgaagtttgaatccctttcctttcttcaatcattgataagaactaataattcaagt : 379
Alha6_Alpp : gacttgaagtttgaatccctttcctttcttcaatcattgataagaactaataattcaagt : 375
Alha7_Alpp : gacttgaagtttgaatccctttcctttcttcaatcattgataagaactaataattcaagt : 381
Alha8_Alpp : gacttgaagtttgaatccctttcctttcttcaatcattgataagaactaataattcaagt : 374
Alha0_Alpp : gacttgaagtttgaatccctttcctttcttcaatcattgataagaactaataattcaagt : 404
Alni1_Alpp : gacttgaagt-------------cttacttcaatcattgataagaactaataattcaagt : 361
Alni2_Alpp : gacttgaagt-------------cttacttcaatcattgataagaactaataattcaagt : 362
Alni3_Alpp : gacttgaagt-------------cttacttcaatcattgataagaactaataattcaagt : 362
Alni4_Alpp : gacttgaagt-------------cttacttcaatcattgataagaactaataattcaagt : 362
Alni6_Alpp : cacttgaagt-------------cttacttcaatcattgataaaaactaataattcaagt : 361
Alni7_Alpp : gacttgaagt-------------cttacttcaatcattgataagaactaataattcaagt : 362
Alni8_Alpp : gacttgaagt-------------cttacttcaatcattgataagaactaataattcaagt : 364
Alni0_Alpp : gacttgaagt-------------cttacttcaatcattgataagaactaataattcaagt : 389
Alof1_Alpp : gacttgaagtttgaatccctttcctttcttcaatcattgataagaactaataattcaagt : 348
Alof2_Alpp : gacttgaagtttgaatccctttcctttcttcaatcattgataagaactaataattcaagt : 346
Alof3_Alpp : gacttgaagtttgaatccctttcctttcttcaatcattgataagaactaataattcaagt : 347
Alof4_Alpp : gacttgaagtttgaatccctttcctttcttcaatcattgataagaactaataattcaagt : 347
Alof6_Alpp : gacttgaagtttgaatccctttcctttcttcaatcattgataagaactaataattcaagt : 346
Alof7_Alpp : gacttgaagtttgaatccctttcctttcttcaatcattgataagaactaataattcaagt : 349
Alof8_Alpp : gacttgaagtttgaatccctttcctttcttcaatcattgataagaactaataattcaagt : 348
Alof0_Alpp : gacttgaagtttgaatccctttcctttcttcaatcattgataagaactaataattcaagt : 375
Alox1_Alpp : gacttgaagtttgaatccctttcctttcttcaatcattgataagaactaataattcaagt : 347
Alox2_Alpp : gacttgaagtttgaatccctttcctttcttcaatcattgataagaactaataattcaagt : 350
Alox3_Alpp : gacttgaagtttgaatccctttcctttcttcaatcattgataagaactaataattcaagt : 348
Alox6_Alpp : gacttgaagtttgaatccctttcctttcttcaatcattgataagaactaataattcaagt : 349
Alox7_Alpp : gacttgaagtttgaatccctttcctttcttcaatcattgataagaactaataattcaagt : 349
Alox0_Alpp : gacttgaagtttgaatccctttcctttcttcaatcattgataagaactaataattcaagt : 375

Alga1_Alpp : ------------------------------------------------------------ :  -
Alga2_Alpp : ttcaatcaaattagtcattttgactgactgtttttacgtagatgataagtaaaaaagcag : 424
Alga3_Alpp : ttcaatcaaattagtcattttgactgactgtttttacgtagatgataagtaaaaaagcag : 425
Alga4_Alpp : ttcaatcaaattagtcattttgactgactgtttttacgtagatgataagtaaaaaagcag : 425
Alga6_Alpp : ------------------------------------------------------------ :  -
Alga7_Alpp : ------------------------------------------------------------ :  -
Alga8_Alpp : ttcaatcaaattagtcattttgactgactgtttttacgtagatgataagtaaaaaagcag : 425
Alga0_Alpp : ttcaatcaaattagtcattttgactgactgtttttacgtagatgataagtaaaaaagcag : 450
Alha1_Alpp : ttcaatcaaattagtcattttgactgactgtttttacgtagatgataagtaaaaaagcag : 437
Alha2_Alpp : ttcaatcaaattagtcattttgactgactgtttttacgtagatgataagtaaaaaagcag : 437
Alha3_Alpp : ttcaatcaaattagtcattttgactgactgtttttacgtagatgataagtaaaaaagcag : 439
Alha6_Alpp : ttcaatcaaattagtcattttgactgactgtttttacgtagatgataagtaaaaaagcag : 435
```

```
Alha7_Alpp : ttcaatcaaattagtcattttgactgactgtttttacgtagatgataagtaaaaaagcag : 441
Alha8_Alpp : ttcaatcaaattagtcattttgactgactgtttttacgtagatgataagtaaaaaagcag : 434
Alha0_Alpp : ttcaatcaaattagtcattttgactgactgtttttacgtagatgataagtaaaaaagcag : 464
Alni1_Alpp : ttcaatcaaattagtcattttgactgactgtttttacgtagatgataagtaaaaaagcag : 421
Alni2_Alpp : ttcaatcaaattagtcattttgactgactgtttttacgtagatgataagtaaaaaagcag : 422
Alni3_Alpp : ttcaatcaaattagtcattttgactgactgtttttacgtagatgataagtaaaaaagcag : 422
Alni4_Alpp : ttcaatcaaattagtcattttgactgactgtttttacgtagatgataagtaaaaaagcag : 422
Alni6_Alpp : ttcaatcgactgactgattttgactgactgattttacaaaaatgataaggaacaaagcag : 421
Alni7_Alpp : ttcaatcaaattagtcattttgactgactgtttttacgtagatgataagtaaaaaagcag : 422
Alni8_Alpp : ttcaatcaaattagtcattttgactgactgtttttacgtagatgataagtaaaaaagcag : 424
Alni0_Alpp : ttcaatcaaattagtcattttgactgactgtttttacgtagatgataagtaaaaaagcag : 449
Alof1_Alpp : ttcaatcaaattagtcattttgactgactgtttttacgtagatgataagtaaaaaagcag : 408
Alof2_Alpp : ttcaatcaaattagtcattttgactgactgtttttacgtagatgataagtaaaaaagcag : 406
Alof3_Alpp : ttcaatcaaattagtcattttgactgactgtttttacgtagatgataagtaaaaaagcag : 407
Alof4_Alpp : ttcaatcaaattagtcattttgactgactgtttttacgtagatgataagtaaaaaagcag : 407
Alof6_Alpp : ttcaatcaaattagtcattttgactgactgtttttacgtagatgataagtaaaaaagcag : 406
Alof7_Alpp : ttcaatcaaattagtcattttgactgactgtttttacgtagatgataagtaaaaaagcag : 409
Alof8_Alpp : ttcaatcaaattagtcattttgactgactgtttttacgtagatgataagtaaaaaagcag : 408
Alof0_Alpp : ttcaatcaaattagtcattttgactgactgtttttacgtagatgataagtaaaaaagcag : 435
Alox1_Alpp : ttcaatcaaattagtcattttgactgactgtttttacgtagatgataagtaaaaaagcag : 407
Alox2_Alpp : ttcaatcaaattagtcattttgactgactgtttttacgtagatgataagtaaaaaagcag : 410
Alox3_Alpp : ttcaatcaaattagtcattttgactgactgtttttacgtagatgataagtaaaaaagcag : 408
Alox6_Alpp : ttcaatcaaattagtcattttgactgactgtttttacgtagatgataagtaaaaaagcag : 409
Alox7_Alpp : ttcaatcaaattagtcattttgactgactgtttttacgtagatgataagtaaaaaagcag : 409
Alox0_Alpp : ttcaatcaaattagtcattttgactgactgtttttacgtagatgataagtaaaaaagcag : 435

Alga1_Alpp : ----------------------------- :  -
Alga2_Alpp : taggaactagaatgaa-cagtgcagta-- : 450
Alga3_Alpp : taggaactagaatgaa-cagtgcagta-- : 451
Alga4_Alpp : taggaactagaatgaa-cagtgcagtac- : 452
Alga6_Alpp : ----------------------------- :  -
Alga7_Alpp : ----------------------------- :  -
Alga8_Alpp : taggaactagaatgaa-cagtgcagta-- : 451
Alga0_Alpp : taggaactagaatgaa-cagtgcagt--- : 475
Alha1_Alpp : taggaactagaatgaa-cagtgcagta-- : 463
Alha2_Alpp : taggaactagaatgaa-cagtgcagta-- : 463
Alha3_Alpp : taggaactagaatgaa-cagtgcagta-- : 465
Alha6_Alpp : taggaactagaatga--cagtgcagta-- : 460
Alha7_Alpp : taggaactagaatgaa-cagtgcagta-- : 467
Alha8_Alpp : taggaactagaatgaa-cagtgcagta-- : 460
Alha0_Alpp : taggaactagaatgaa-cagtgcagt--- : 489
Alni1_Alpp : taggaactagaatgaa-cagtgcagta-- : 447
Alni2_Alpp : taggaactagaatgaa-cagtgcagtaaa : 450
Alni3_Alpp : taggaactagaatgaa-cagtgcagta-- : 448
Alni4_Alpp : taggaactagaatgaa-cagtgcagta-- : 448
Alni6_Alpp : aacgaactagaatgaa-cagtgcagta-- : 447
Alni7_Alpp : taggaactagaatgaa-cagtgcagta-- : 448
Alni8_Alpp : taggaactagaatgaa-cagtgcagta-- : 450
Alni0_Alpp : taggaactagaatgaa-cagtgcagt--- : 474
Alof1_Alpp : taggaactagaatgaa-cagtgcagta-- : 434
Alof2_Alpp : taggaactagaatga--cagtgcagta-- : 431
Alof3_Alpp : taggaactagaatgaa-cagtgcagta-- : 433
Alof4_Alpp : taggaactagaatga--cagtgcagta-- : 432
Alof6_Alpp : taggaactagaatgaa-cagtgcagta-- : 432
Alof7_Alpp : taggaactagaatgaa-cagtgcagta-- : 435
Alof8_Alpp : taggaactagaatgaacaagtgcagta-- : 435
Alof0_Alpp : taggaactagaatgaa-cagtgcagt--- : 460
Alox1_Alpp : taggaactagaatgaa-cagtgcagta-- : 433
Alox2_Alpp : taggaactagaatgaa-cagtgcagta-- : 436
Alox3_Alpp : taggaactagaatga--cagtgcagta-- : 433
Alox6_Alpp : taggaactagaatgaa-cagtgcagta-- : 435
Alox7_Alpp : taggaactagaatgaa-cagtgcagta-- : 435
Alox0_Alpp : taggaactagaatgaa-cagtgcagt--- : 460
```

Figure S14. The alignment of amplicons produced by designed Alpr primers.

The ID of each sequence is shown on the left side of each panel. The ID is the concatenation of species name, plant individual id, and primer name. The rightmost number in each column represents the position of the base at that position in the amplicon. The SNPs and Indel are shown in red squares. The nucleotides identical across all plastomes are shaded in black, whereas those conserved in 60% of the sequences are shaded in gray. *Alpinia galanga*: Alga; *A. hainanensis*: Alha; *A. nigra*: Alni; *A. officinarum*: Alof; *A. oxyphylla*: Alox. Arabic numerals represent different individuals.

```
Alga1_Alpr : gggg------------------------------tgagagagatacttaa :  21
Alga2_Alpr : c-ggg-----------------------------atagagagatactt-a :  19
Alga3_Alpr : g-ggg-----------------------------gaagagagatactt-a :  19
Alga4_Alpr : t-gac-----------------------------gcgaagagatactt-a :  19
Alga6_Alpr : cgggg-----------------------------cgagagagatactt-a :  20
Alga7_Alpr : aggaa--------------------------taaggaggaagatacttaa :  24
Alga8_Alpr : g-gga-----------------------------gagaagagatactt-a :  19
Alga0_Alpr : tagggagggttccgctaaatcatagcatagaaaagaaagaagatacttaa :  50
Alha1_Alpr : gggg--------------------------------tggagagatctt-a :  17
Alha2_Alpr : gggg--------------------------------tcggagatactt-a :  17
Alha3_Alpr : gggg--------------------------------atgagagaactt-a :  17
Alha6_Alpr : agg-----------------------------------agagagtctt-a :  14
Alha7_Alpr : aggg--------------------------------atgagagtctta-a :  17
Alha8_Alpr : cgga----------------------------------ggagagtctt-a :  15
Alha0_Alpr : tagggagggttccgctaaatcatagcataga-----aagaagatacttaa :  45
Alni1_Alpr : gcggt-----------------------------gagaagagatactt-a :  20
Alni3_Alpr : -agga-----------------------------gagaagagatactt-a :  19
Alni3_Alpr : ggggc-----------------------------taggagagatactt-a :  20
Alni4_Alpr : --ggg-----------------------------cgagagagatactt-a :  18
Alni6_Alpr : --ggg-----------------------------ctagagagatactt-a :  18
Alni7_Alpr : tgggc----------------------------gaagaagagatacttaa :  22
Alni8_Alpr : cgggg----------------------------ctgagagagatactt-a :  21
Alni0_Alpr : tagggagggttccgctaaatcatagcatagaaaagaaagaagatacttaa :  50
Alof1_Alpr : ggacg---------------------------------aagagaactt-a :  16
Alof2_Alpr : agatt-------------------------------aaagaggaactt-a :  18
Alof3_Alpr : aggat---------------------------------aagaggtctt-a :  16
Alof4_Alpr : gggac-------------------------------tgaggagaacttaa :  19
Alof5_Alpr : ggggc--------------------------------taggagaactt-a :  17
Alof6_Alpr : aggac--------------------------------gaggagaactt-a :  17
Alof7_Alpr : cgggg---------------------------------tagagaacttaa :  17
Alof8_Alpr : gtgga--------------------------------cgagagaactt-a :  17
Alof0_Alpr : tagggagggttccgctaaatcatagcataga-----aagaagatacttaa :  45
Alox1_Alpr : ggggc--------------------------------gagaagaactt-a :  17
Alox2_Alpr : ggggc--------------------------------gaggagaactt-a :  17
Alox6_Alpr : gtgga---------------------------------tagagatctt-a :  16
Alox7_Alpr : gggaa------------------------------taaggaggaactt-a :  19
Alox0_Alpr : tagggagggttccgctaaatcatagcataga-----aagaagatacttaa :  45

Alga1_Alpr : attaaataaaaataataatttagtggatttaggataggaacaattgatag :  71
Alga2_Alpr : attaaataaaaataataatttagtggatttaggataggaacaattgatag :  69
Alga3_Alpr : attaaataaaaataataatttagtggatttaggataggaacaattgatag :  69
Alga4_Alpr : attaaataaaaataataatttagtggatttaggataggaacaattgatag :  69
Alga6_Alpr : attaaataaaaataataatttagtggatttaggataggaacaattgatag :  70
Alga7_Alpr : attaaataaaaataataatttagtggatttaggataggaacaattgatag :  74
Alga8_Alpr : attaaataaaaataataatttagtggatttaggataggaacaattgatag :  69
Alga0_Alpr : attaaataaaaataataatttagtggatttaggataggaacaattgatag : 100
Alha1_Alpr : a-ttaataaaaataataatttagtggatttaggataggaacaattgatag :  66
Alha2_Alpr : a-ttaataaaaataataatttagtggatttaggataggaacaattgatag :  66
Alha3_Alpr : a-ttaataaaaataataatttagtggatttaggataggaacaattgatag :  66
Alha6_Alpr : a-ttaataaaaataataatttagtggatttaggataggaacaattgatag :  63
Alha7_Alpr : a-ttaataaaaataataatttagtggatttaggataggaacaattgatag :  66
Alha8_Alpr : a-ttaataaaaataataatttagtggatttaggataggaacaattgatag :  64
Alha0_Alpr : attaaataaaaataataatttagtggatttaggataggaacaattgatag :  95
Alni1_Alpr : attaaataaaaataataatttagtggatttaggataggaacaatggatag :  70
Alni3_Alpr : attaaataaaaataataatttagtggatttaggataggaacaatggatag :  69
Alni3_Alpr : attaaataaaaataataatttagtggatttaggataggaacaatggatag :  70
Alni4_Alpr : attaaataaaaataataatttagtggatttaggataggaacaatggatag :  68
Alni6_Alpr : attaaataaaaataataatttagtggatttaggataggaacaatggatag :  68
Alni7_Alpr : attaaataaaaataataatttagtggatttaggataggaacaatggatag :  72
Alni8_Alpr : attaaataaaaataataatttagtggatttaggataggaacaatggatag :  71
```

```
Alni0_Alpr : attaaataaaaataataatttagtggatttaggataggaacaatggatag : 100
Alof1_Alpr : a-ttaataaa----------tagtggatttaggataggaacaattgatag :  55
Alof2_Alpr : a-ttaataaa----------tagtggatttaggataggaacaattgatag :  57
Alof3_Alpr : a-ttaataaa----------tagtggatttaggataggaacaattgatag :  55
Alof4_Alpr : a-ttaataaa----------tagtggatttaggataggaacaattgatag :  58
Alof5_Alpr : a-ttaataaa----------tagtggatttaggataggaacaattgatag :  56
Alof6_Alpr : a-ttaataaa----------tagtggatttaggataggaacaattgatag :  56
Alof7_Alpr : attaaataaa----------tagtggatttaggataggaacaattgatag :  57
Alof8_Alpr : a-ttaataaa----------tagtggatttaggataggaacaattgatag :  56
Alof0_Alpr : attaaataaa----------tagtggatttaggataggaacaattgatag :  85
Alox1_Alpr : a-ttaataaa----------tagtggatttaggataggaacaattgatag :  56
Alox2_Alpr : a-ttaataaa----------tagtggatttaggataggaacaattgatag :  56
Alox6_Alpr : a-ttaataaa----------tagtggatttaggataggaacaattgatag :  55
Alox7_Alpr : a-ttaataaa----------tagtggatttaggataggaacaattgatag :  58
Alox0_Alpr : attaaataaa----------tagtggatttaggataggaacaattgatag :  85

Alga1_Alpr : ttagaaagaaattgtatttcttaattattacttcataaaattattatttt : 121
Alga2_Alpr : ttagaaagaaattgtatttcttaattattacttcataaaattattatttt : 119
Alga3_Alpr : ttagaaagaaattgtatttcttaattattacttcataaaattattatttt : 119
Alga4_Alpr : ttagaaagaaattgtatttcttaattattacttcataaaattattatttt : 119
Alga6_Alpr : ttagaaagaaattgtatttcttaattattacttcataaaattattatttt : 120
Alga7_Alpr : ttagaaagaaattgtatttcttaattattacttcataaaattattatttt : 124
Alga8_Alpr : ttagaaagaaattgtatttcttaattattacttcataaaattattatttt : 119
Alga0_Alpr : ttagaaagaaattgtatttcttaattattacttcataaaattattatttt : 150
Alha1_Alpr : ttagaaagaaattgtatttcttaattattacttcataaaattattatttt : 116
Alha2_Alpr : ttagaaagaaattgtatttcttaattattacttcataaaattattatttt : 116
Alha3_Alpr : ttagaaagaaattgtatttcttaattattacttcataaaattattatttt : 116
Alha6_Alpr : ttagaaagaaattgtatttcttaattattacttcataaaattattatttt : 113
Alha7_Alpr : ttagaaagaaattgtatttcttaattattacttcataaaattattatttt : 116
Alha8_Alpr : ttagaaagaaattgtatttcttaattattacttcataaaattattatttt : 114
Alha0_Alpr : ttagaaagaaattgtatttcttaattattacttcataaaattattatttt : 145
Alni1_Alpr : ttagaaaaaaactgtatttcttaattattacttcataaaattattatttt : 120
Alni3_Alpr : ttagaaaaaaactgtatttcttaattattacttcataaaattattatttt : 119
Alni3_Alpr : ttagaaaaaaactgtatttcttaattattacttcataaaattattatttt : 120
Alni4_Alpr : ttagaaaaaaactgtatttcttaattattacttcataaaattattatttt : 118
Alni6_Alpr : ttagaaaaaaactgtatttcttaattattacttcataaaattattatttt : 118
Alni7_Alpr : ttagaaaaaaactgtatttcttaattattacttcataaaattattatttt : 122
Alni8_Alpr : ttagaaaaaaactgtatttcttaattattacttcataaaattattatttt : 121
Alni0_Alpr : ttagaaaaaaactgtatttcttaattattacttcataaaattattatttt : 150
Alof1_Alpr : ttagaaataaattgtatttcttaattattacttcataaaattattatttt : 105
Alof2_Alpr : ttagaaataaattgtatttcttaattattacttcataaaattattatttt : 107
Alof3_Alpr : ttagaaataaattgtatttcttaattattacttcataaaattattatttt : 105
Alof4_Alpr : ttagaaataaattgtatttcttaattattacttcataaaattattatttt : 108
Alof5_Alpr : ttagaaataaattgtatttcttaattattacttcataaaattattatttt : 106
Alof6_Alpr : ttagaaataaattgtatttcttaattattacttcataaaattattatttt : 106
Alof7_Alpr : ttagaaataaattgtatttcttaattattacttcataaaattattatttt : 107
Alof8_Alpr : ttagaaataaattgtatttcttaattattacttcataaaattattatttt : 106
Alof0_Alpr : ttagaaataaattgtatttcttaattattacttcataaaattattatttt : 135
Alox1_Alpr : ttagaaataaattgtatttcttaattattacttcataaaattattatttt : 106
Alox2_Alpr : ttagaaataaattgtatttcttaattattacttcataaaattattatttt : 106
Alox6_Alpr : ttagaaataaattgtatttcttaattattacttcataaaattattatttt : 105
Alox7_Alpr : ttagaaataaattgtatttcttaattattacttcataaaattattatttt : 108
Alox0_Alpr : ttagaaataaattgtatttcttaattattacttcataaaattattatttt : 135

Alga1_Alpr : attactctatatctatat-------------------------------- : 139
Alga2_Alpr : attactctatatctatat-------------------------------- : 137
Alga3_Alpr : attactctatatctatat-------------------------------- : 137
Alga4_Alpr : attactctatatctatat-------------------------------- : 137
Alga6_Alpr : attactctatatctatat-------------------------------- : 138
Alga7_Alpr : attactctatatctatat-------------------------------- : 142
Alga8_Alpr : attactctatatctatat-------------------------------- : 137
Alga0_Alpr : attactctatatctatat-------------------------------- : 168
Alha1_Alpr : attactctatatctatat-------------------------------- : 134
Alha2_Alpr : attactctatatctatat-------------------------------- : 134
Alha3_Alpr : attactctatatctatat-------------------------------- : 134
Alha6_Alpr : attactctatatctatat-------------------------------- : 131
Alha7_Alpr : attactctatatctatat-------------------------------- : 134
Alha8_Alpr : attactctatatctatat-------------------------------- : 132
Alha0_Alpr : attactctatatctatat-------------------------------- : 163
Alni1_Alpr : attactctatatctatat-------------------------------- : 138
Alni3_Alpr : attactctatatctatat-------------------------------- : 137
Alni3_Alpr : attactctatatctatat-------------------------------- : 138
```

```
Alni4_Alpr : attactctatatctatat-------------------------------- : 136
Alni6_Alpr : attactctatatctatat-------------------------------- : 136
Alni7_Alpr : attactctatatctatat-------------------------------- : 140
Alni8_Alpr : attactctatatctatat-------------------------------- : 139
Alni0_Alpr : attactctatatctatatatctataaaatataaaggtaaaatttttactc : 200
Alof1_Alpr : attactctatatctatat-------------------------------- : 123
Alof2_Alpr : attactctatatctatat-------------------------------- : 125
Alof3_Alpr : attactctatatctatat-------------------------------- : 123
Alof4_Alpr : attactctatatctatat-------------------------------- : 126
Alof5_Alpr : attactctatatctatat-------------------------------- : 124
Alof6_Alpr : attactctatatctatat-------------------------------- : 124
Alof7_Alpr : attactctatatctatat-------------------------------- : 125
Alof8_Alpr : attactctatatctatat-------------------------------- : 124
Alof0_Alpr : attactctatatctatat-------------------------------- : 153
Alox1_Alpr : attactctatatctatat-------------------------------- : 124
Alox2_Alpr : attactctatatctatat-------------------------------- : 124
Alox6_Alpr : attactctatatctatat-------------------------------- : 123
Alox7_Alpr : attactctatatctatat-------------------------------- : 126
Alox0_Alpr : attactctatatctatat-------------------------------- : 153

Alga1_Alpr : ---------atctataaaatatcaaagtaaaatttttacttaa------- : 173
Alga2_Alpr : ---------atctataaaatatcaaagtaaaatttttacttaa------- : 171
Alga3_Alpr : ---------atctataaaatatcaaagtaaaatttttacttaa------- : 171
Alga4_Alpr : ---------atctataaaatatcaaagtaaaatttttacttaa------- : 171
Alga6_Alpr : ---------atctataaaatatcaaagtaaaatttttacttaa------- : 172
Alga7_Alpr : ---------atctataaaatatcaaagtaaaatttttacttaa------- : 176
Alga8_Alpr : ---------atctataaaatatcaaagtaaaatttttacttaa------- : 171
Alga0_Alpr : ---------atctataaaatatcaaagtaaaatttttacttaa------- : 202
Alha1_Alpr : ---------atctataaaatataaaagtaaaatttttacttaa------- : 168
Alha2_Alpr : ---------atctataaaatataaaagtaaaatttttacttaa------- : 168
Alha3_Alpr : ---------atctataaaatataaaagtaaaatttttacttaa------- : 168
Alha6_Alpr : ---------atctataaaatataaaagtaaaatttttacttaa------- : 165
Alha7_Alpr : ---------atctataaaatataaaagtaaaatttttacttaa------- : 168
Alha8_Alpr : ---------atctataaaatataaaagtaaaatttttacttaa------- : 166
Alha0_Alpr : ---------atctataaaatataaaagtaaaatttttacttaa------- : 197
Alni1_Alpr : ---------atctataaaatataaaggtaaaatttttacttaa------- : 172
Alni3_Alpr : ---------atctataaaatataaaggtaaaatttttacttaa------- : 171
Alni3_Alpr : ---------atctataaaatataaaggtaaaatttttacttaa------- : 172
Alni4_Alpr : ---------atctataaaatataaaggtaaaatttttacttaa------- : 170
Alni6_Alpr : ---------atctataaaatataaaggtaaaatttttacttaa------- : 170
Alni7_Alpr : ---------atctataaaatataaaggtaaaatttttacttaa------- : 174
Alni8_Alpr : ---------atctataaaatataaaggtaaaatttttacttaa------- : 173
Alni0_Alpr : taaatcttaatctataaaatataaaggtaaaatttttacttaa------- : 243
Alof1_Alpr : ---------atctataaaatataaaagtaaaatttttacttaatcttaat : 164
Alof2_Alpr : ---------atctataaaatataaaagtaaaatttttacttaatcttaat : 166
Alof3_Alpr : ---------atctataaaatataaaagtaaaatttttacttaatcttaat : 164
Alof4_Alpr : ---------atctataaaatataaaagtaaaatttttacttaatcttaat : 167
Alof5_Alpr : ---------atctataaaatataaaagtaaaatttttacttaatcttaat : 165
Alof6_Alpr : ---------atctataaaatataaaagtaaaatttttacttaatcttaat : 165
Alof7_Alpr : ---------atctataaaatataaaagtaaaatttttacttaatcttaat : 166
Alof8_Alpr : ---------atctataaaatataaaagtaaaatttttacttaatcttaat : 165
Alof0_Alpr : ---------atctataaaatataaaagtaaaatttttacttaatcttaat : 194
Alox1_Alpr : ---------atctataaaatataaaagtaaaatttttacttaa------- : 158
Alox2_Alpr : ---------atctataaaatataaaagtaaaatttttacttaa------- : 158
Alox6_Alpr : ---------atctataaaatataaaagtaaaatttttacttaa------- : 157
Alox7_Alpr : ---------atctataaaatataaaagtaaaatttttacttaa------- : 160
Alox0_Alpr : ---------atctataaaatataaaagtaaaatttttacttaa------- : 187

Alga1_Alpr : -tctta-----attacattaat---------------------attaatt : 196
Alga2_Alpr : -tctta-----attacattaat---------------------attaatt : 194
Alga3_Alpr : -tctta-----attacattaat---------------------attaatt : 194
Alga4_Alpr : -tctta-----attacattaat---------------------attaatt : 194
Alga6_Alpr : -tctta-----attacattaat---------------------attaatt : 195
Alga7_Alpr : -tctta-----attacattaat---------------------attaatt : 199
Alga8_Alpr : -tctta-----attacattaat---------------------attaatt : 194
Alga0_Alpr : -tctta-----attacattaattttattatcttaattacattaattaatt : 246
Alha1_Alpr : -tctta-----attacattaa----------------------------t : 184
Alha2_Alpr : -tctta-----attacattaa----------------------------t : 184
Alha3_Alpr : -tctta-----attacattaa----------------------------t : 184
Alha6_Alpr : -tctta-----attacattaa----------------------------t : 181
Alha7_Alpr : -tctta-----attacattaa----------------------------t : 184
Alha8_Alpr : -tctta-----attacattaa----------------------------t : 182
```

```
Alha0_Alpr : -tctta-----attacattaa----------------------------t : 213
Alni1_Alpr : -tcttaattacattacattaat---------------------attaatt : 200
Alni3_Alpr : -tcttaattacattacattaat---------------------attaatt : 199
Alni3_Alpr : -tcttaattacattacattaat---------------------attaatt : 200
Alni4_Alpr : -tcttaattacattacattaat---------------------attaatt : 198
Alni6_Alpr : -tcttaattacattacattaat---------------------attaatt : 198
Alni7_Alpr : -tcttaattacattacattaat---------------------attaatt : 202
Alni8_Alpr : -tcttaattacattacattaat---------------------attaatt : 201
Alni0_Alpr : -tcttaattacattacattaat---------------------attaatt : 271
Alof1_Alpr : ttcttaattacattacattaa----------------------------t : 186
Alof2_Alpr : ttcttaattacgttacattaa----------------------------t : 188
Alof3_Alpr : ttcttaattacattacattaa----------------------------t : 186
Alof4_Alpr : ttcttaattacattacattaa----------------------------t : 189
Alof5_Alpr : ttcttaattacattacattaa----------------------------t : 187
Alof6_Alpr : ttcttaattacattacattaa----------------------------t : 187
Alof7_Alpr : ttcttaattacattacattaa----------------------------t : 188
Alof8_Alpr : ttcttaattacattacattaa----------------------------t : 187
Alof0_Alpr : ttcttaattacattacattaa----------------------------t : 216
Alox1_Alpr : -tcttaattacattacattaat---------------------attaatt : 186
Alox2_Alpr : -tcttaattacattacattaat---------------------attaatt : 186
Alox6_Alpr : -tcttaattacattacattaat---------------------attaatt : 185
Alox7_Alpr : -tcttaattacattacattaat---------------------attaatt : 188
Alox0_Alpr : -tcttaattacattacattaat---------------------attaatt : 215

Alga1_Alpr : attaattaagaattt----aat------aaataagaatttaatgataatg : 236
Alga2_Alpr : attaattaagaattt----aat------aaataagaatttaatgataatg : 234
Alga3_Alpr : attaattaagaattt----aat------aaataagaatttaatgataatg : 234
Alga4_Alpr : attaattaagaattt----aat------aaataagaatttaatgataatg : 234
Alga6_Alpr : attaattaagaattt----aat------aaataagaatttaatgataatg : 235
Alga7_Alpr : attaattaagaattt----aat------aaataagaatttaatgataatg : 239
Alga8_Alpr : attaattaagaattt----aat------aaataagaatttaatgataatg : 234
Alga0_Alpr : attaattaagaatttaataaat------aaataagaatttaatgataatg : 290
Alha1_Alpr : attaattattaattt----aat------aaataagaatt------tcatg : 218
Alha2_Alpr : attaattattaattt----aat------aaataagaatt------tcatg : 218
Alha3_Alpr : attaattattaattt----aat------aaataagaatt------tcatg : 218
Alha6_Alpr : attaattattaattt----aat------aaataagaatt------tcatg : 215
Alha7_Alpr : attaattattaattt----aat------aaataagaatt------tcatg : 218
Alha8_Alpr : attaattattaattt----aat------aaataagaatt------tcatg : 216
Alha0_Alpr : attaattattaattt----aat------aaataagaatt------tcatg : 247
Alni1_Alpr : attaattaagaattt----aat------aaataagaatt------taatg : 234
Alni3_Alpr : attaattaagaattt----aat------aaataagaatt------taatg : 233
Alni3_Alpr : attaattaagaattt----aat------aaataagaatt------taatg : 234
Alni4_Alpr : attaattaagaattt----aat------aaataagaatt------taatg : 232
Alni6_Alpr : attaattaagaattt----aat------aaataagaatt------taatg : 232
Alni7_Alpr : attaattaagaattt----aat------aaataagaatt------taatg : 236
Alni8_Alpr : attaattaagaattt----aat------aaataagaatt------taatg : 235
Alni0_Alpr : attaattaagaattt----aat------aaataagaatt------taatg : 305
Alof1_Alpr : attaattaataattt----aataaataaaaataagaatt------tcatg : 226
Alof2_Alpr : attaattaataattt----aataaataaaaataagaatt------tcatg : 228
Alof3_Alpr : attaattaataattt----aataaataaaaataagaatt------tcatg : 226
Alof4_Alpr : attaattaataattt----aataaataaaaataagaatt------tcatg : 229
Alof5_Alpr : attaattaataattt----aataaataaaaataagaatt------tcatg : 227
Alof6_Alpr : attaattaataattt----aataaataaaaataagaatt------tcatg : 227
Alof7_Alpr : attaattaataattt----aataaataaaaataagaatt------tcatg : 228
Alof8_Alpr : attaattaataattt----aataaataaaaataagaatt------tcatg : 227
Alof0_Alpr : attaattaataattt----aataaataaaaataagaatt------tcatg : 256
Alox1_Alpr : attaattaataattt----aataaataaaaataagaatt------tcatg : 226
Alox2_Alpr : attaattaataattt----aataaataaaaataagaatt------tcatg : 226
Alox6_Alpr : attaattaataattt----aataaataaaaataagaatt------tcatg : 225
Alox7_Alpr : attaattaataattt----aataaataaaaataagaatt------tcatg : 228
Alox0_Alpr : attaattaataattt----aataaataaaaataagaatt------tcatg : 255

Alga1_Alpr : ataattgcaacgaaattttataaaattctatttctagttagtcactttt : 286
Alga2_Alpr : ataattgcaacgaaattttataaaattctatttctagttagtcactttt : 284
Alga3_Alpr : ataattgcaacgaaattttataaaattctatttctagttagtcactttt : 284
Alga4_Alpr : ataattgcaacgaaattttataaaattctatttctagttagtcactttt : 284
Alga6_Alpr : ataattgcaacgaaattttataaaattctatttctagttagtcactttt : 285
Alga7_Alpr : ataattgcaacgaaattttataaaattctatttctagttagtcactttt : 289
Alga8_Alpr : ataattgcaacgaaattttataaaattctatttctagttagtcactttt : 284
Alga0_Alpr : ataattgcaacgaaattttataaaattctatttctagttagtcactttt : 340
Alha1_Alpr : ataattgcaacgaaattttagaaaattctatttctagttagtcactttt : 268
Alha2_Alpr : ataattgcaacgaaattttagaaaattctatttctagttagtcactttt : 268
```

```
Alha3_Alpr : ataattgcaacgaaattttagaaaattctatttctagttagtcactttt : 268
Alha6_Alpr : ataattgcaacgaaattttagaaaattctatttctagttagtcactttt : 265
Alha7_Alpr : ataattgcaacgaaattttagaaaattctatttctagttagtcactttt : 268
Alha8_Alpr : ataattgcaacgaaattttagaaaattctatttctagttagtcactttt : 266
Alha0_Alpr : ataattgcaacgaaattttagaaaattctatttctagttagtcactttt : 297
Alni1_Alpr : ataattgcaacgaaattttagaaaattatatttctagttagtcactttt : 284
Alni3_Alpr : ataattgcaacgaaattttagaaaattatatttctagttagtcactttt : 283
Alni3_Alpr : ataattgcaacgaaattttagaaaattatatttctagttagtcactttt : 284
Alni4_Alpr : ataattgcaacgaaattttagaaaattatatttctagttagtcactttt : 282
Alni6_Alpr : ataattgcaacgaaattttagaaaattatatttctagttagtcactttt : 282
Alni7_Alpr : ataattgcaacgaaattttagaaaattatatttctagttagtcactttt : 286
Alni8_Alpr : ataattgcaacgaaattttagaaaattatatttctagttagtcactttt : 285
Alni0_Alpr : ataattgcaacgaaattttagaaaattatatttctagttagtcactttt : 355
Alof1_Alpr : ataattgcaacgaaattttagaaaattctatttctagttagtcactttt : 276
Alof2_Alpr : ataattgcaacgaaattttagaaaattctatttctagttagtcactttt : 278
Alof3_Alpr : ataattgcaacgaaattttagaaaattctatttctagttagtcactttt : 276
Alof4_Alpr : ataattgcaacgaaattttagaaaattctatttctagttagtcactttt : 279
Alof5_Alpr : ataattgcaacgaaattttagaaaattctatttctagttagtcactttt : 277
Alof6_Alpr : ataattgcaacgaaattttagaaaattctatttctagttagtcactttt : 277
Alof7_Alpr : ataattgcaacgaaattttagaaaattctatttctagttagtcactttt : 278
Alof8_Alpr : ataattgcaacgaaattttagaaaattctatttctagttagtcactttt : 277
Alof0_Alpr : ataattgcaacgaaattttagaaaattctatttctagttagtcactttt : 306
Alox1_Alpr : ataattgcaacgaaattttagaaaattctatttctagttagtcactttt : 276
Alox2_Alpr : ataattgcaacgaaattttagaaaattctatttctagttagtcactttt : 276
Alox6_Alpr : ataattgcaacgaaattttagaaaattctatttctagttagtcactttt : 275
Alox7_Alpr : ataattgcaacgaaattttagaaaattctatttctagttagtcactttt : 278
Alox0_Alpr : ataattgcaacgaaattttagaaaattctatttctagttagtcactttt : 305

Alga1_Alpr : atttaatttattttgttttcttcttcttcagctcagatcgaaaatataa : 336
Alga2_Alpr : atttaatttattttgttttcttcttcttcagctcagatcgaaaatataa : 334
Alga3_Alpr : atttaatttattttgttttcttcttcttcagctcagatcgaaaatataa : 334
Alga4_Alpr : atttaatttattttgttttcttcttcttcagctcagatcgaaaatataa : 334
Alga6_Alpr : atttaatttattttgttttcttcttcttcagctcagatcgaaaatataa : 335
Alga7_Alpr : atttaatttattttgttttcttcttcttcagctcagatcgaaaatataa : 339
Alga8_Alpr : atttaatttattttgttttcttcttcttcagctcagatcgaaaatataa : 334
Alga0_Alpr : atttaatttattttgttttcttcttcttcagctcagatcgaaaatataa : 390
Alha1_Alpr : atttcatttattttgttttcttcttcttcagctcagatcgaaaatataa : 318
Alha2_Alpr : atttcatttattttgttttcttcttcttcagctcagatcgaaaatataa : 318
Alha3_Alpr : atttcatttattttgttttcttcttcttcagctcagatcgaaaatataa : 318
Alha6_Alpr : atttcatttattttgttttcttcttcttcagctcagatcgaaaatataa : 315
Alha7_Alpr : atttcatttattttgttttcttcttcttcagctcagatcgaaaatataa : 318
Alha8_Alpr : atttcatttattttgttttcttcttcttcagctcagatcgaaaatataa : 316
Alha0_Alpr : atttcatttattttgttttcttcttcttcagctcagatcgaaaatataa : 347
Alni1_Alpr : atttaatttattttgttttcttcttcttcagctcagatcgaaaatataa : 334
Alni3_Alpr : atttaatttattttgttttcttcttcttcagctcagatcgaaaatataa : 333
Alni3_Alpr : atttaatttattttgttttcttcttcttcagctcagatcgaaaatataa : 334
Alni4_Alpr : atttaatttattttgttttcttcttcttcagctcagatcgaaaatataa : 332
Alni6_Alpr : atttaatttattttgttttcttcttcttcagctcagatcgaaaatataa : 332
Alni7_Alpr : atttaatttattttgttttcttcttcttcagctcagatcgaaaatataa : 336
Alni8_Alpr : atttaatttattttgttttcttcttcttcagctcagatcgaaaatataa : 335
Alni0_Alpr : atttaatttattttgttttcttcttcttcagctcagatcgaaaatataa : 405
Alof1_Alpr : atttcatttattttgttttcttcttcttcagctcagatcgaaaatataa : 326
Alof2_Alpr : atttcatttattttgttttcttcttcttcagctcagatcgaaaatataa : 328
Alof3_Alpr : atttcatttattttgttttcttcttcttcagctcagatcgaaaatataa : 326
Alof4_Alpr : atttcatttattttgttttcttcttcttcagctcagatcgaaaatataa : 329
Alof5_Alpr : atttcatttattttgttttcttcttcttcagctcagatcgaaaatataa : 327
Alof6_Alpr : atttcatttattttgttttcttcttcttcagctcagatcgaaaatataa : 327
Alof7_Alpr : atttcatttattttgttttcttcttcttcagctcagatcgaaaatataa : 328
Alof8_Alpr : atttcatttattttgttttcttcttcttcagctcagatcgaaaatataa : 327
Alof0_Alpr : atttcatttattttgttttcttcttcttcagctcagatcgaaaatataa : 356
Alox1_Alpr : atttcatttattttgttttcttcttcttcagctcagatcgaaaatataa : 326
Alox2_Alpr : atttcatttattttggtttcttcttcttcagctcagatcgaaaatataa : 326
Alox6_Alpr : atttcatttattttgttttcttcttcttcagctcagatcgaaaatataa : 325
Alox7_Alpr : atttcatttattttgttttcttcttcttcagctcagatcgaaaatataa : 328
Alox0_Alpr : atttcatttattttgttttcttcttcttcagctcagatcgaaaatataa : 355

Alga1_Alpr : gagttaagccgatacaaaat--gaaaaggggtttatggctaaggg-taa : 383
Alga2_Alpr : gagttaagccgatacaaaat--gaaaaggggtttatggtaaaggg-taa : 381
Alga3_Alpr : gagttaagccgatacaaaat--gaaaaggggtttatggctaaagggtaa : 382
Alga4_Alpr : gagttaagccgatacaaaat--gaaaaggggtttatggctaaggg-taa : 381
Alga6_Alpr : gagttaagccgatacaaaat--gaaaaggggtttatggtaaaggg-taa : 382
Alga7_Alpr : gagttaagccgatacaaaat--gaaaaggggtttatggctaaggg-taa : 386
```

```
Alga8_Alpr : gagttaagccgatacaaaat--gaaaagggggtttatggt-aaggg-taa : 380
Alga0_Alpr : gagttaagccgatacaaaat--gaaaagggggtttatggctaaggg-taa : 437
Alha1_Alpr : gagttaagccgatacaaaat--ttaaagggggtttatggctaaggg-taa : 365
Alha2_Alpr : ga------------------------------------------------ : 320
Alha3_Alpr : gagttaagccgatacaaaat--ttaaagggggtttatggctaaggg-taa : 365
Alha6_Alpr : gagttaagccgatacaaaat--ttaaagggggtttatggctaagggtaa : 363
Alha7_Alpr : gagttaagccgatacaaaat--ttaaagggggtttatggctaaggg-taa : 365
Alha8_Alpr : gagttaagccgatacaaaat--ttaaagggggtttatggctaaggg-taa : 363
Alha0_Alpr : gagttaagccgatacaaaat--ttaaagggggtttatggctaaggg-taa : 394
Alni1_Alpr : gagttaagccgatacaaaataaaaaaagggggtttatggctaaggg-taa : 383
Alni3_Alpr : gagttaagccgatacaaaataaaaaaagggggtttatggtaaaggg-taa : 382
Alni3_Alpr : gagttaagccgatacaaaataaaaaaagggggtttatggctaaggg-taa : 383
Alni4_Alpr : gagttaagccgatacaaaataaaaaaagggggtttatggctaaggg-taa : 381
Alni6_Alpr : gagttaagccgatacaaaataaaaaaagggggtttatggtaaaggg-taa : 381
Alni7_Alpr : gagttaagccgatacaaaataaaaaaagggggtttatggctaaagg-gta : 385
Alni8_Alpr : gagttaagccgatacaaaataaaaaaagggggtttatggctaaggg-taa : 384
Alni0_Alpr : gagttaagccgatacaaaataaaaaaagggggtttatggctaaggg-taa : 454
Alof1_Alpr : gagttaagccgatacaaaat--taaaagggggtttatggctaaggg-taa : 373
Alof2_Alpr : gagttaagccgatacaaaat--taaaagggggtttatggctaagggtaa : 376
Alof3_Alpr : gagttaagccgatacaaaat--taaaagggggtttatggctaaggg-taa : 373
Alof4_Alpr : gagttaagccgatacaaaat--taaaagggggtttatggctaaggg-taa : 376
Alof5_Alpr : gagttaagccgatacaaaat--taaaagggggtttatggcgaaggg-taa : 374
Alof6_Alpr : gagttaagccgatacaaaat--taaaagggggtttatggctaaggg-taa : 374
Alof7_Alpr : gagttaagccgatacaaaat--taaaagggggtttatggtaaaggg-taa : 375
Alof8_Alpr : gagttaagccgatacaaaat--taaaagggggtttatggctaaggg-taa : 374
Alof0_Alpr : gagttaagccgatacaaaat--taaaagggggtttatggctaaggg-taa : 403
Alox1_Alpr : gagttaagccgatacaaaat--ttaaagggggtttatggctaaggg-taa : 373
Alox2_Alpr : gagttaagccgatacaaaat--ttaaagggggtttatggctaaggg-taa : 373
Alox6_Alpr : gagttaagccgatacaaaat--ttaaagggggtttatggctaaggg-taa : 372
Alox7_Alpr : gagttaagccgatacaaaat--ttaaagggggtttatggctaaggg-taa : 375
Alox0_Alpr : gagttaagccgatacaaaat--ttaaagggggtttatggctaaggg-taa : 402

Alga1_Alpr : ga------------------------------------------------ : 385
Alga2_Alpr : ga------------------------------------------------ : 383
Alga3_Alpr : ga------------------------------------------------ : 384
Alga4_Alpr : ga------------------------------------------------ : 383
Alga6_Alpr : ga------------------------------------------------ : 384
Alga7_Alpr : gagtgctctttcttcccctgctcgttgtgggggagcggagggataaaact : 436
Alga8_Alpr : ga------------------------------------------------ : 382
Alga0_Alpr : g------------------------------------------------- : 438
Alha1_Alpr : ga------------------------------------------------ : 367
Alha2_Alpr : -------------------------------------------------- :   -
Alha3_Alpr : gatttaggggaaccctccctaact-------------------------- : 389
Alha6_Alpr : gaagtggggatggtttgggtt----------------------------- : 384
Alha7_Alpr : gatttaggggaaccctccctaatg-------------------------- : 389
Alha8_Alpr : ga------------------------------------------------ : 365
Alha0_Alpr : g------------------------------------------------- : 395
Alni1_Alpr : ga------------------------------------------------ : 385
Alni3_Alpr : ga------------------------------------------------ : 384
Alni3_Alpr : ga------------------------------------------------ : 385
Alni4_Alpr : ga------------------------------------------------ : 383
Alni6_Alpr : gatttagcggaaccctccctaa---------------------------- : 403
Alni7_Alpr : agatgagcggaccctccctaatc--------------------------- : 408
Alni8_Alpr : gatttagcggaacccctccctaa--------------------------- : 407
Alni0_Alpr : g------------------------------------------------- : 455
Alof1_Alpr : g------------------------------------------------- : 374
Alof2_Alpr : ga------------------------------------------------ : 378
Alof3_Alpr : gatttgggggaccctttgc------------------------------- : 392
Alof4_Alpr : ga------------------------------------------------ : 378
Alof5_Alpr : ga------------------------------------------------ : 376
Alof6_Alpr : gattcggggaccctgtaccga----------------------------- : 395
Alof7_Alpr : ga------------------------------------------------ : 377
Alof8_Alpr : ga------------------------------------------------ : 376
Alof0_Alpr : g------------------------------------------------- : 404
Alox1_Alpr : ga------------------------------------------------ : 375
Alox2_Alpr : ga------------------------------------------------ : 375
Alox6_Alpr : gatttagcggaaccctccctaa---------------------------- : 394
Alox7_Alpr : ga------------------------------------------------ : 377
Alox0_Alpr : g------------------------------------------------- : 403

Alga1_Alpr : ------------------------------------------ :   -
Alga2_Alpr : ------------------------------------------ :   -
```

```
Alga3_Alpr : ---------------------------------------- :   -
Alga4_Alpr : ---------------------------------------- :   -
Alga6_Alpr : ---------------------------------------- :   -
Alga7_Alpr : attattttatttaataaattatcccctttcttttttattc : 477
Alga8_Alpr : ---------------------------------------- :   -
Alga0_Alpr : ---------------------------------------- :   -
Alha1_Alpr : ---------------------------------------- :   -
Alha2_Alpr : ---------------------------------------- :   -
Alha3_Alpr : ---------------------------------------- :   -
Alha6_Alpr : ---------------------------------------- :   -
Alha7_Alpr : ---------------------------------------- :   -
Alha8_Alpr : ---------------------------------------- :   -
Alha0_Alpr : ---------------------------------------- :   -
Alni1_Alpr : ---------------------------------------- :   -
Alni3_Alpr : ---------------------------------------- :   -
Alni3_Alpr : ---------------------------------------- :   -
Alni4_Alpr : ---------------------------------------- :   -
Alni6_Alpr : ---------------------------------------- :   -
Alni7_Alpr : ---------------------------------------- :   -
Alni8_Alpr : ---------------------------------------- :   -
Alni0_Alpr : ---------------------------------------- :   -
Alof1_Alpr : ---------------------------------------- :   -
Alof2_Alpr : ---------------------------------------- :   -
Alof3_Alpr : ---------------------------------------- :   -
Alof4_Alpr : ---------------------------------------- :   -
Alof5_Alpr : ---------------------------------------- :   -
Alof6_Alpr : ---------------------------------------- :   -
Alof7_Alpr : ---------------------------------------- :   -
Alof8_Alpr : ---------------------------------------- :   -
Alof0_Alpr : ---------------------------------------- :   -
Alox1_Alpr : ---------------------------------------- :   -
Alox2_Alpr : ---------------------------------------- :   -
Alox6_Alpr : ---------------------------------------- :   -
Alox7_Alpr : ---------------------------------------- :   -
Alox0_Alpr : ---------------------------------------- :   -
```

Figure S15. The alignment of amplicons in 10 *Alpinia* plastomes produced by designed Alpp primers in silico.

The ID of each sequence is shown on the left side of each panel. The ID is the accession numbers of species in GenBank (Table S24). The rightmost number in each column represents the position of the base at that position in the amplicon. The SNPs and Indel are shown in red squares. The nucleotides identical across all plastomes are shaded in black, whereas those conserved in 60% of the sequences are shaded in gray.

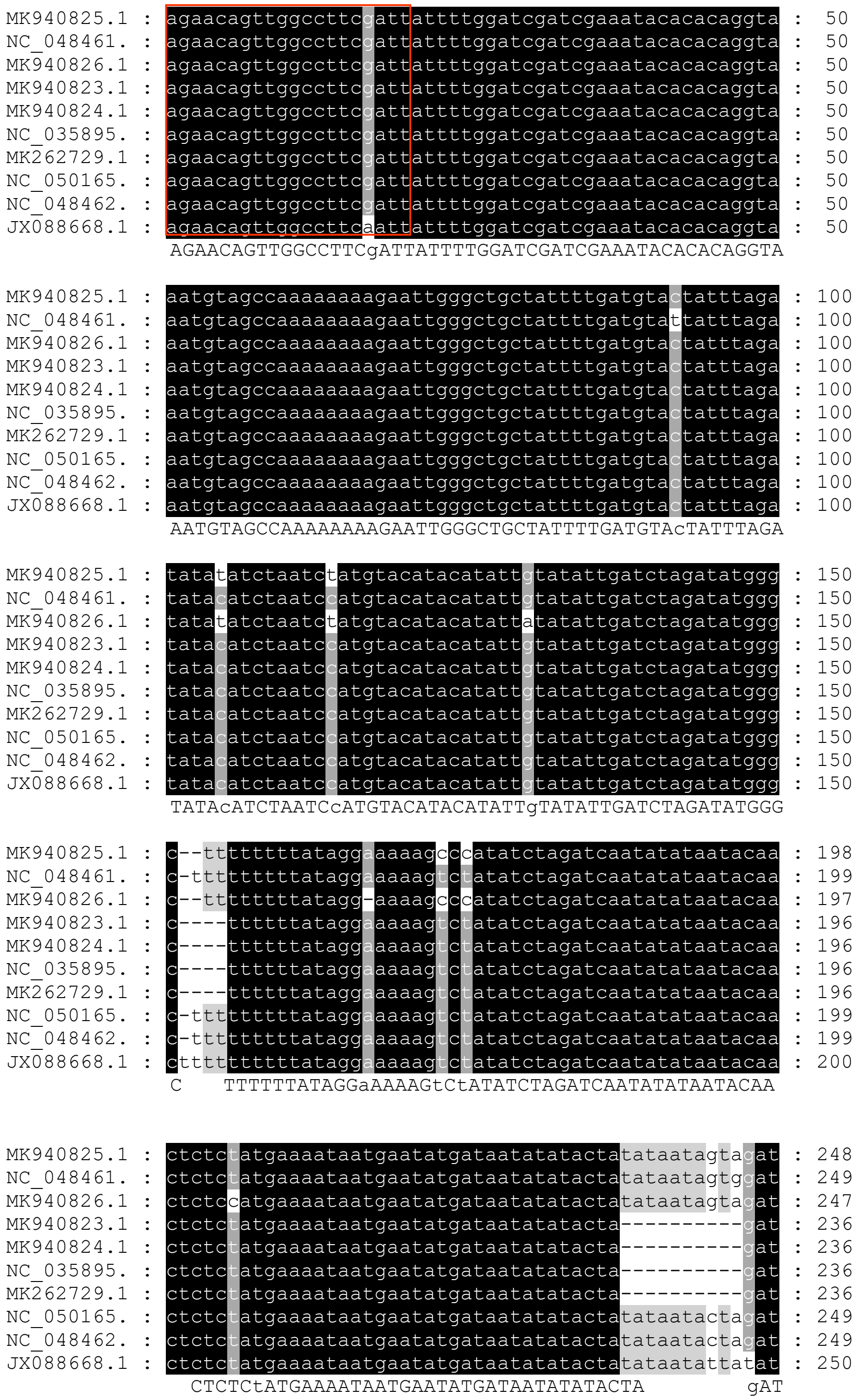

```
MK940825.1 : aactataccatactatctaggcttagatagtactatctcagatacttatt : 298
NC_048461. : aactataccatactatccaggcttagatagtactatctcagatacttatt : 299
MK940826.1 : aactataccatactatctaggcttagatagtactatctaagatacttatt : 297
MK940823.1 : aactataccatactatctaggcttagatagtactatctcagatac----- : 281
MK940824.1 : aactataccatactatctaggcttagatagtactatctcagatac----- : 281
NC_035895. : aactataccatactatctaggcttagatagtactatctcagatac----- : 281
MK262729.1 : aactataccatactatctaggcttagatagtactatctcagatac----- : 281
NC_050165. : aactataccatactatctaggcttagatagtactatctcagatac----- : 294
NC_048462. : aactataccatactatctaggcttagatagtactatctcagatac----- : 294
JX088668.1 : aactataccatactatccaggcttagatagtactatctcagatacttatt : 300
             AACTATACCATACTATCtAGGCTTAGATAGTACTATCTcAGATAC

MK940825.1 : atctcagatacttatgattccagccagatcatttctttcgtttaagactt : 348
NC_048461. : atctcagatatttatgattccagccagatcatttctttcgtttaagactt : 349
MK940826.1 : atctcagatacttattattccagccggatcatttctttcgtttaagactt : 347
MK940823.1 : -----------ttatgattccagccagatcatttctttcgtttaagactt : 320
MK940824.1 : -----------ttatgattccagccagatcatttctttcgtttaagactt : 320
NC_035895. : -----------ttatgattccagccagatcatttctttcgtttaagactt : 320
MK262729.1 : -----------ttatgattccagccagatcatttctttcgtttaagactt : 320
NC_050165. : -----------ttatgattccagccagatcatttctttcgtttaagactt : 333
NC_048462. : -----------ttatgattccagccagatcatttctttcgtttaagactt : 333
JX088668.1 : atctcagatatttatgattccagccagatcatttctttcgtttaagactt : 350
                        TTATgATTCCAGCCaGATCATTTCTTTCGTTTAAGACTT

MK940825.1 : aaagtttga-------------cttcaatcattgataagaactaataatt : 385
NC_048461. : gaagtttgaatccctttcctttcttcaatcattgataagaactaataatt : 399
MK940826.1 : gaagtctta-------------cttcaatcattgataagaactaataatt : 384
MK940823.1 : gaagtttgaatccctttcctttcttcaatcattgataagaactaataatt : 370
MK940824.1 : gaagtttgaatccctttcctttcttcaatcattgataagaactaataatt : 370
NC_035895. : gaagtttgaatccctttcctttcttcaatcattgataagaactaataatt : 370
MK262729.1 : gaagtttgaatccctttcctttcttcaatcattgataagaactaataatt : 370
NC_050165. : gaagtttgaatccctttcctttcttcaatcattgataagaactaataatt : 383
NC_048462. : gaagtttgaatccctttcctttcttcaatcattgataagaactaataatt : 383
JX088668.1 : gaagtttgaatccctttcctttcttcaatcattgataagaactaataatt : 400
             gAAGTtTgAatccctttcctttCTTCAATCATTGATAAGAACTAATAATT

MK940825.1 : caagtttcaatcaaattagtcattttgactgactgtttttacgtagatga : 435
NC_048461. : caagtttcaatcaaattagtcattttgactgactgtttttacgtagatga : 449
MK940826.1 : caagtttcaatcaaattagtcattttgactgactgtttttacgtagatga : 434
MK940823.1 : caagtttcaatcaaattagtcattttgactgactgtttttacgtagatga : 420
MK940824.1 : caagtttcaatcaaattagtcattttgactgactgtttttacgtagatga : 420
NC_035895. : caagtttcaatcaaattagtcattttgactgactgtttttacgtagatga : 420
MK262729.1 : caagtttcaatcaaattagtcattttgactgactgtttttacgtagatga : 420
NC_050165. : caagtttcaatcaaattagtcattttgactgactgtttttacgtagatga : 433
NC_048462. : caagtttcaatcaaattagtcattttgactgactgtttttacgtagatga : 433
JX088668.1 : caagtttcaatcaaattagtcattttgactgactgtttttacgtagatga : 450
          CAAGTTTCAATCAAATTAGTCATTTTGACTGACTGTTTTTACGTAGATGA

MK940825.1 : taagtaaaaaagcagtaggaactagaatgaacagtgcagt : 475
NC_048461. : taagtaaaaaagcagtaggaactagaatgaacagtgcagt : 489
MK940826.1 : taagtaaaaaagcagtaggaactagaatgaacagtgcagt : 474
MK940823.1 : taagtaaaaaagcagtaggaactagaatgaacagtgcagt : 460
MK940824.1 : taagtaaaaaagcagtaggaactagaatgaacagtgcagt : 460
NC_035895. : taagtaaaaaagcagtaggaactagaatgaacagtgcagt : 460
MK262729.1 : taagtaaaaaagcagtaggaactagaatgaacagtgcagt : 460
NC_050165. : taagtaaaaaagcagtaggaactagaatgaacagtgcagt : 473
NC_048462. : taagtaaaaaagcagtaggaactagaatgaacagtgcagt : 473
JX088668.1 : taagtaaaaaagcagtaggaactagaatgaacagtgcagt : 490
             TAAGTAAAAAAGCAGTAGGAACTAGAATGAACAGTGCAGT
```

Figure S16. The alignment of amplicons in 10 *Alpinia* plastomes produced by designed Alpr primers in silico.

The ID of each sequence is shown on the left side of each panel. The ID is the accession numbers of species in NCBI (Table S24). The rightmost number in each column represents the position of the base at that position in the amplicon. The SNPs and Indel are shown in red squares. The nucleotides identical across all plastomes are shaded in black, whereas those conserved in 60% of the sequences are shaded in gray.

```
MK940825.1 : tagggagggttccgctaaatcatagcatagaaaagaaagaagatacttaa :  50
NC_048461. : tagggagggttccgctaaatcatagcat-----agaaagaagatacttaa :  45
MK940826.1 : tagggagggttccgctaaatcatagcatagaaaagaaagaagatacttaa :  50
MK940823.1 : tagggagggttccgctaaatcatagcat-----agaaagaagatacttaa :  45
MK940824.1 : tagggagggttccgctaaatcatagcat-----agaaagaagatacttaa :  45
NC_035895. : tagggagggttccgctaaatcatagcat-----agaaagaagatacttaa :  45
MK262729.1 : tagggagggttccgctaaatcatagcat-----agaaagaagatacttaa :  45
NC_050165. : tagggagggttccgctaaatcatagcat-----agaaagaagatacttaa :  45
NC_048462. : tagggagggttccgctaaatcatagcat-----agaaagaagatacttaa :  45
JX088668.1 : tagggagggttccgctaaatcatagcat-----agaaagaagatacttaa :  45
              TAGGGAGGGTTCCGCTAAATCATAGCAT     AGAAAGAAGATACTTAA

MK940825.1 : attaaataaaaataataatttagtggatttaggataggaacaatttgatag : 100
NC_048461. : attaaataaaaataataatttagtggatttaggataggaacaatttgatag :  95
MK940826.1 : attaaataaaaataataatttagtggatttaggataggaacaatggatag : 100
MK940823.1 : attaaataaa----------tagtggatttaggataggaacaatttgatag :  85
MK940824.1 : attaaataaa----------tagtggatttaggataggaacaatttgatag :  85
NC_035895. : attaaataaa----------tagtggatttaggataggaacaatttgatag :  85
MK262729.1 : attaaataaa----------tagtggatttaggataggaacaatttgatag :  85
NC_050165. : attaaataaaaataataatttagtggatttaggatgggaacaatttgatag :  95
NC_048462. : attaaataaaaataataatttagtggatttaggatgggaacaatttgatag :  95
JX088668.1 : attaaataaaaataataatttagtggatttaggataggaacaatttgatag :  95
              ATTAAATAAA          TAGTGGATTTAGGATaGGAACAATtGATAG

MK940825.1 : ttagaaagaaattgtatttcttaattattacttcataaaattattatttt : 150
NC_048461. : ttagaaagaaattgtatttcttaattattacttcataaaattattatttt : 145
MK940826.1 : ttagaaaaaaactgtatttcttaattattacttcataaaattattatttt : 150
MK940823.1 : ttagaaataaattgtatttcttaattattacttcataaaattattatttt : 135
MK940824.1 : ttagaaataaattgtatttcttaattattacttcataaaattattatttt : 135
NC_035895. : ttagaaataaattgtatttcttaattattacttcataaaattattatttt : 135
MK262729.1 : ttagaaataaattgtatttcttaattattacttcataaaattattatttt : 135
NC_050165. : ttagaaataaattgtatttcttaattattacttcataaaattattatttt : 145
NC_048462. : ttagaaataaattgtatttcttaattattacttcataaaattattatttt : 145
JX088668.1 : ttagaaataaattgtatttcttaattattacttcataaaattattatttt : 145
              TTAGAAA AAAtTGTATTTCTTAATTATTACTTCATAAAATTATTATTTT

MK940825.1 : attactctatatctatat-------------------------------- : 168
NC_048461. : attactctatatctatat-------------------------------- : 163
MK940826.1 : attactctatatctatatatctataaaatataaaggtaaaatttttactc : 200
MK940823.1 : attactctatatctatat-------------------------------- : 153
MK940824.1 : attactctatatctatat-------------------------------- : 153
NC_035895. : attactctatatctatat-------------------------------- : 153
MK262729.1 : attactctatatctatat-------------------------------- : 153
NC_050165. : attactc--------tat-------------------------------- : 155
NC_048462. : attactc--------tat-------------------------------- : 155
JX088668.1 : attactctatatctatat-------------------------------- : 163
              ATTACTCtatatctaTAT

MK940825.1 : ---------atctataaaatatcaaagtaaaatttttacttaa------- : 202
NC_048461. : ---------atctataaaatataaaagtaaaatttttacttaa------- : 197
MK940826.1 : taaatcttaatctataaaatataaaggtaaaatttttacttaa------- : 243
MK940823.1 : ---------atctataaaatataaaagtaaaatttttacttaatcttaat : 194
MK940824.1 : ---------atctataaaatataaaagtaaaatttttacttaa------- : 187
NC_035895. : ---------atctataaaatataaaagtaaaatttttacttaa------- : 187
MK262729.1 : ---------atctataaaatataaaagtaaaatttttacttaa------- : 187
NC_050165. : ---------atctataaaatataaaagtaaaatttttacttaa------- : 189
NC_048462. : ---------atctataaaatataaaagtaaaatttttacttaa------- : 189
JX088668.1 : ---------atctataaaatataaaagtaaaatttttacttaa------- : 197
                       ATCTATAAAATATaAAaGTAAAATTTTTACTTAA
```

```
MK940825.1 : -tcttaattacattaattttattatcttaattacattaa--ttaattatt : 249
NC_048461. : -tctta-----------------------attacattaa-------tatt : 216
MK940826.1 : -tcttaattac------------------attacattaatattaattatt : 274
MK940823.1 : ttcttaattac------------------attacattaa-------tatt : 219
MK940824.1 : -tcttaattac------------------attacattaatattaattatt : 218
NC_035895. : -tcttaattac------------------attacattaatattaattatt : 218
MK262729.1 : -tcttaattac------------------attacattaatattaattatt : 218
NC_050165. : -tcttaattac------------------attacattaa-------tatt : 213
NC_048462. : -tcttaattac------------------attacattaa-------tatt : 213
JX088668.1 : -tctta-----------------------attacattaa-------tatt : 216
              TCTTAattac                  ATTACATTAA       TATT


MK940825.1 : aattaagaatttaataaat--aaataagaatttaatgataatgataattg : 297
NC_048461. : aattattaatttaat------aaataagaatt------tcatgataattg : 254
MK940826.1 : aattaagaatttaat------aaataagaatt------taatgataattg : 312
MK940823.1 : aattaataatttaataaataaaaataagaatt------tcatgataattg : 263
MK940824.1 : aattaataatttaataaataaaaataagaatt------tcatgataattg : 262
NC_035895. : aattaataatttaataaataaaaataagaatt------tcatgataattg : 262
MK262729.1 : aattaataatttaataaataaaaataagaatt------tcatgataattg : 262
NC_050165. : aattaataatttaat------aaataagaatt------tcatgataattg : 251
NC_048462. : aattaataatttaat------aaataagaatt------tcatgataattg : 251
JX088668.1 : aattattaatttaat------aaataagaatt------tcatgataattg : 254
             AATTAatAATTTAAT      AAATAAGAATT      TcATGATAATTG


MK940825.1 : caacgaaatttttataaaattctatttctagttagtcacttttatttaat : 347
NC_048461. : caacgaaatttttagaaaattctatttctagttagtcacttttatttcat : 304
MK940826.1 : caacgaaatttttagaaaattatatttctagttagtcacttttatttaat : 362
MK940823.1 : caacgaaatttttagaaaattctatttctagttagtcacttttatttcat : 313
MK940824.1 : caacgaaatttttagaaaattctatttctagttagtcacttttatttcat : 312
NC_035895. : caacgaaatttttagaaaattctatttctagttagtcacttttatttcat : 312
MK262729.1 : caacgaaatttttagaaaattctatttctagttagtcacttttatttcat : 312
NC_050165. : caacgaaatttttagaaaattctatttctagttagtcacttttatttcat : 301
NC_048462. : caacgaaatttttagaaaattctatttctagttagtcacttttatttcat : 301
JX088668.1 : caacgaaatttttagaaaattctatttctagttagtcacttttatttcat : 304
             CAACGAAATTTTTAgAAAATTcTATTTCTAGTTAGTCACTTTTATTTcAT


MK940825.1 : ttatttttgttttcttcttcttcagctcagatcgaaaatataagagttaa : 397
NC_048461. : ttatttttgttttcttcttcttcagctcagatcgaaaatataagagttaa : 354
MK940826.1 : ttatttttgttttcttcttcttcagctcagatcgaaaatataagagttaa : 412
MK940823.1 : ttatttttgttttcttcttcttcagctcagatcgaaaatataagagttaa : 363
MK940824.1 : ttatttttgttttcttcttcttcagctcagatcgaaaatataagagttaa : 362
NC_035895. : ttatttttgttttcttcttcttcagctcagatcgaaaatataagagttaa : 362
MK262729.1 : ttatttttgttttcttcttcttcagctcagatcgaaaatataagagttaa : 362
NC_050165. : ttatttttgttttcttcttcttcagctcagatcgaaaatataagagttaa : 351
NC_048462. : ttatttttgttttcttcttcttaagctcagatcgaaaatataagagttaa : 351
JX088668.1 : ttatttttgttttcttcttcttcagctcagatcgaaaatataagagttaa : 354
             TTATTTTTGTTTTCTTCTTCTTcAGCTCAGATCGAAAATATAAGAGTTAA


MK940825.1 : gccgatacaaaat--gaaaagggggtttatggctaagggtaag : 438
NC_048461. : gccgatacaaaat--ttaaagggggtttatggctaagggtaag : 395
MK940826.1 : gccgatacaaaataaaaaaagggggtttatggctaagggtaag : 455
MK940823.1 : gccgatacaaaat--taaaagggggtttatggctaagggtaag : 404
MK940824.1 : gccgatacaaaat--ttaaagggggtttatggctaagggtaag : 403
NC_035895. : gccgatacaaaat--ttaaagggggtttatggctaagggtaag : 403
MK262729.1 : gccgatacaaaat--ttaaagggggtttatggctaagggtaag : 403
NC_050165. : gccgatacaaaat--ttaaagggggtttatggctaagggtaag : 392
NC_048462. : gccgatacaaaat--ttaaagggggtttatggctaagggtaag : 392
JX088668.1 : gccgatacaaaat--ttaaagggggtttatggctaagggtaag : 395
             GCCGATACAAAAT  t AAAGGGGGTTTATGGCTAAGGGTAAG
```
